# Thermodynamic System Drift in Protein Evolution

**DOI:** 10.1371/journal.pbio.1001994

**Published:** 2014-11-11

**Authors:** Kathryn M. Hart, Michael J. Harms, Bryan H. Schmidt, Carolyn Elya, Joseph W. Thornton, Susan Marqusee

**Affiliations:** 1 Department of Chemistry, University of California, Berkeley, Berkeley, California, United States of America; 2 Institute for Quantitative Biosciences (QB3), University of California, Berkeley, Berkeley, California, United States of America; 3 Institute of Molecular Biology, University of Oregon, Eugene, Oregon, United States of America; 4 Department of Molecular & Cell Biology, University of California, Berkeley, Berkeley, California, United States of America; 5 Department of Human Genetics and Department of Ecology and Evolution, University of Chicago, Chicago, Illinois, United States of America; Brandeis University, United States of America

## Abstract

Proteins from thermophiles are generally more thermostable than their mesophilic homologs, but little is known about the evolutionary process driving these differences. Here we attempt to understand how the diverse thermostabilities of bacterial ribonuclease H1 (RNH) proteins evolved. RNH proteins from *Thermus thermophilus* (ttRNH) and *Escherichia coli* (ecRNH) share similar structures but differ in melting temperature (*T_m_*) by 20°C. ttRNH's greater stability is caused in part by the presence of residual structure in the unfolded state, which results in a low heat capacity of unfolding (*ΔC_p_*) relative to ecRNH. We first characterized RNH proteins from a variety of extant bacteria and found that *T_m_* correlates with the species' growth temperatures, consistent with environmental selection for stability. We then used ancestral sequence reconstruction to statistically infer evolutionary intermediates along lineages leading to ecRNH and ttRNH from their common ancestor, which existed approximately 3 billion years ago. Finally, we synthesized and experimentally characterized these intermediates. The shared ancestor has a melting temperature between those of ttRNH and ecRNH; the *T_m_*s of intermediate ancestors along the ttRNH lineage increased gradually over time, while the ecRNH lineage exhibited an abrupt drop in *T_m_* followed by relatively little change. To determine whether the underlying mechanisms for thermostability correlate with the changes in *T_m_*, we measured the thermodynamic basis for stabilization—*ΔC_p_* and other thermodynamic parameters—for each of the ancestors. We observed that, while the *T_m_* changes smoothly, the mechanistic basis for stability fluctuates over evolutionary time. Thus, even while overall stability appears to be strongly driven by selection, the proteins explored a wide variety of mechanisms of stabilization, a phenomenon we call “thermodynamic system drift.” This suggests that even on lineages with strong selection to increase stability, proteins have wide latitude to explore sequence space, generating biophysical diversity and potentially opening new evolutionary pathways.

## Abstract 2

Tracking the evolution of thermostability in resurrected ancestors of a heat-tolerant extremophile protein and its less heat tolerant *Escherichia coli* homologue shows how thermostability has probably explored different mechanisms of protein stabilization over evolutionary time.

## Author Summary

The biophysical properties of proteins must adjust to accommodate environmental temperatures because of the narrow range over which any given protein sequence can remain folded and functional. We compared the evolution of homologous bacterial enzymes (ribonucleases H1) from two lineages: one from *Escherichia coli*, which live at moderate temperatures, the other from *Thermus thermophilus*, which live at extremely high temperatures. Our aim was to investigate how these structurally homologous proteins can have such different thermostabilities, unfolding at temperatures that are 20°C apart. We used bioinformatics to reconstruct the sequences of ancestral proteins along each lineage, synthesized the proteins in the lab, and experimentally traced the evolution of ribonuclease H1 stability. While thermostability appears to have been strongly shaped by selection, the biophysical mechanisms used to tune protein stability appear to have varied throughout evolutionary history; this suggests that proteins have wide latitude to explore different mechanisms of stabilization, generating biophysical diversity and opening up new evolutionary pathways.

## Introduction

Protein thermostability is almost certainly tuned by natural selection. The fold of a protein is sensitive to denaturation at high temperatures: above the melting temperature (*T_m_*) proteins lose structure, function, and become prone to aggregation. In laboratory evolution experiments, selection for growth at elevated temperatures leads to increases in *T_m_* [1]. In natural systems, proteins from thermophilic organisms tend to have higher *T_m_*s than homologs from their mesophilic counterparts [2]. Finally, there are good theoretical reasons to believe that natural selection, but not neutral drift, can lead to a sustained increase in *T_m_*: because random amino acid substitutions tend to decrease protein stability, the final *T_m_* of a protein is expected to be the result of a balance between selection to maintain adequate stability and mutational pressure that drives stability downward [3].

Given the functional importance of thermostability, as well as its utility in protein engineering, many studies have characterized the mechanisms by which stability is achieved [4],[5]. Detailed comparisons of mesophilic and thermophilic homologs have revealed many differences that increase *T_m_*, such as novel interactions in the folded state and residual structure in the unfolded state. These underlying biophysical differences, and the sequence differences that encode them, are usually interpreted as the direct product of selection during adaption to high-temperature environments [5],[6]. Such narratives regarding natural selection, however, are essentially “just-so” stories with little or no empirical justification [7]: many of the mechanistic differences between proteins could reflect pleiotropic association with other properties, neutral drift, or other evolutionary processes.

The extent to which *T_m_* and the mechanisms that determine it are driven by the same selective pressures can be assessed by measuring the co-variation of these protein properties over evolutionary time. If they correlate, the simplest explanation is that the same selective pressure shapes both the *T_m_* and the mechanism of stabilization simultaneously. If, in contrast, the *T_m_* and mechanism of stabilization vary independently, then selection for stability need not imply selection for mechanism. This would, in turn, imply that proteins can explore alternate stabilization mechanisms by neutral drift or selection for some other phenotype while maintaining stability.

A strategy to measure this correlation is to trace and compare the historical evolution of thermostability in mesophilic and thermophilic homologs of the same protein. Such ancient evolutionary trajectories and trends can be studied using the technique of ancestral sequence reconstruction (ASR), which uses the sequences and phylogenetic relationships of modern proteins to statistically infer the sequences of ancient proteins [8]–[13]. The ancient proteins can then be synthesized and experimentally characterized, providing a set of “vertical” comparisons along an evolutionary trajectory, rather than purely “horizontal” comparisons between modern homologs [14].

In this study, we traced the evolutionary and mechanistic origins of the thermodynamic differences between mesophilic and thermophilic ribonuclease H1 (RNH) proteins. RNH is a nonspecific endonuclease that degrades RNA within RNA:DNA hybrids [15]. It exhibits a broad taxonomic distribution and is highly amenable to phylogenetic investigation. Further, the energetic similarities and differences between the mesophile *E. coli* RNH (ecRNH) and its homolog from the thermophile *T. thermophilus* (ttRNH) have been studied extensively [16]–[19]. Here, we measured the stability of a wide variety of extant RNH proteins and demonstrated that their melting temperatures and global stabilities correlate with their organismal growth temperatures, consistent with selection to maintain sufficient stability at the organisms' growth temperatures. We then reconstructed ancestral proteins along the evolutionary lineages that connect ecRNH and ttRNH. We found that, while *T_m_* exhibits a smooth trend along both lineages, the thermodynamic underpinnings of stability fluctuate across a large range of values. These results imply that the evolutionary processes that shape thermostability and the mechanism by which stability is achieved are uncoupled, even within a protein family.

## Results

### The Stabilities of Extant RNH Proteins Correlate with Environmental Temperature

**Figure 1:**
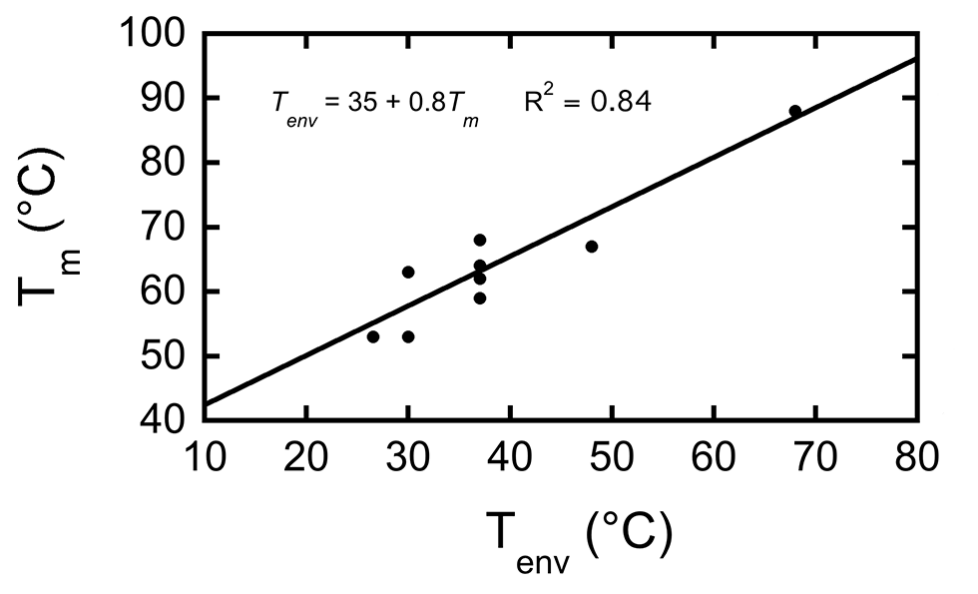
Organismal growth temperatures correlate with thermostability in extant RNH proteins. There is a strong correlation between the melting temperature of RNH and its host organism's growth temperature, suggesting thermostability is product of environmental selection. See also Table S1.

To characterize the relationship between RNH thermostability and environmental temperature (*T_env_*), we measured the energetics of ten modern RNH proteins from organisms with a wide variety of optimal growth temperatures [20],[21]. Growth temperatures were culled from the literature [22],[23], with the exception of two mesophiles for which laboratory culture temperatures were used [24],[25]. We determined the proteins' melting temperatures by carrying out thermally induced denaturation studies monitoring the circular dichroism (CD) signal at 222 nm (see Materials and Methods) (Table S1). We observe a strong correlation (R^2^ = 0.84) between *T_env_* and *T_m_* (Figure 1; Table S1), despite uncertainty in the exact values of environmental growth temperature. Overall, for every 10°C increase in growth temperature, the *T_m_* increases by about 7–8°C. This trend remains even after removing the most thermostable protein, which is from *T. thermophilus* (Figure 1).

We also measured the global stabilities (*ΔG_unf_*)—the free energy of unfolding, which determines the ratio of unfolded and folded molecules at a given temperature—for a subset of these proteins over a range of temperatures. Unfolding free energies were measured by GdmCl-induced denaturation monitored by the change in CD signal at 222 nm (Table S1) and fit using a two-state linear extrapolation model. We found that *ΔG_unf_* was similar for all proteins at their environmental temperatures, ranging from 4.6–8.1 kcal mol^−1^ (average 6.28±1.5 kcal mol^−1^). Together, these results suggest that RNH stability has evolved to accommodate diverse environmental temperatures across bacterial taxa.

### Ancestral Sequence Reconstruction along Two RNH Lineages

**Figure 2:**
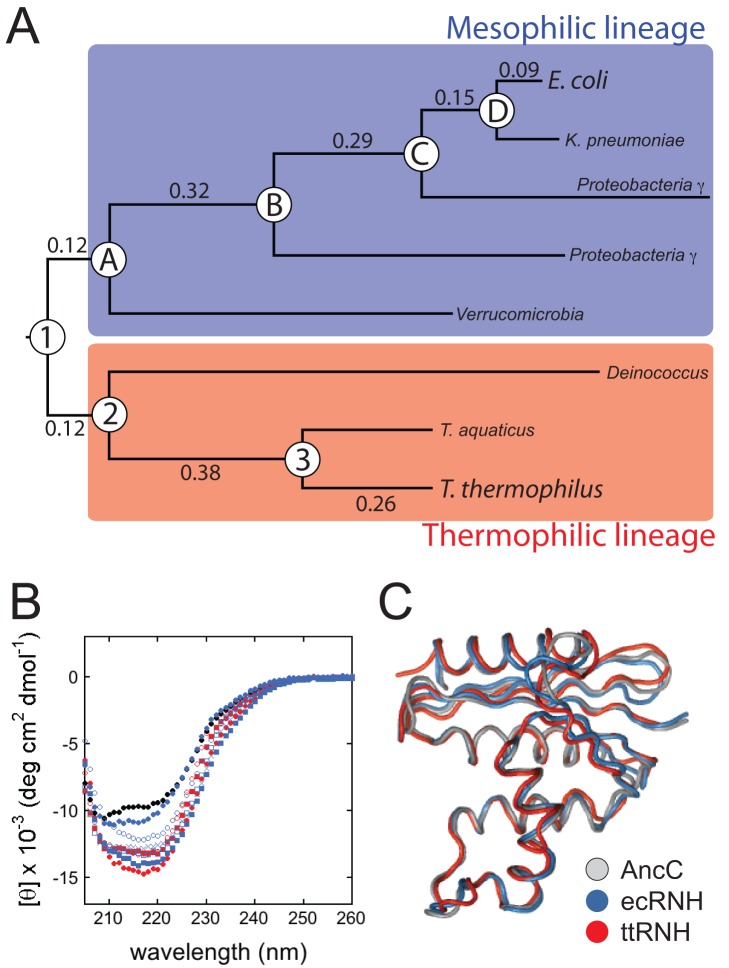
Reconstructed ancestral RNH proteins are functionally and structurally similar to their extant descendants. Structural characterization of ancestral RNH proteins indicates they adopt the canonical RNH fold and closely resemble their extant descendants. (A) Simplified phylogram of the RNH tree indicating positions of resurrected ancestors. Branches on the tree are labeled with their lengths measured in average number of substitutions per site (see Figure S2). (B) CD spectra of ecRNH (blue solid circles), ttRNH (red solid circles), Anc1 (black solid circles), ancestors from the mesophilic lineage (AncA, blue Xs; AncB, blue open squares; AncC, blue solid squares; AncD, blue open circles), and ancestors from the thermophilic lineage (Anc2, red solid squares; Anc3, red open circles) at 25°C in 20 mM NaOAc (pH 5.5), 50 mM KCl, and 1 mM TCEP (see Dataset S2). (C) Superposition of AncC (grey; PDB ID: 4LY7) with ecRNH (blue; PDB ID: 2RN2) and ttRNH (red; PDB ID: 1RIL) for residues 3-145. See also Table S3.

We then used ASR to trace the divergence of the mesophilic ecRNH and thermophilic ttRNH from their common ancestor. Using 409 representative bacterial RNH protein sequences (Figure S1), we inferred the best-fit substitution model and maximum likelihood phylogeny. We then reconstructed the amino acid sequence at seven ancestral nodes along two lineages starting from their most recent common ancestor (Figure 2A). Anc1 represents the most recent common ancestor of ecRNH and ttRNH and is estimated to have existed approximately 3 billion years ago [26]. The other resurrected sequences are evolutionary intermediates at successive phylogenetic nodes along the lineage from Anc1 to ecRNH (AncA through AncD) and from Anc1 to ttRNH (Anc2 and Anc3). Intermediate nodes were chosen for their strong statistical support and for being spaced similarly along the two lineages: Anc2 and AncA each share 92% identity with Anc1, whereas Anc3 and AncB are 77% and 70% identical to Anc1, respectively (Figure S2). We performed the ASR using this unrooted bacterial phylogeny. To order the ancestral nodes in time, we rooted the tree by adding 45 archaeal RNH sequences to the bacterial alignment. The archaeal sequences formed a well-supported clade, providing an unambiguous root. The relative relationships of ecRNH, ttRNH, and their ancestors remained unchanged upon the addition of the archaeal sequences (Figure S2A).

The inferred ancestral sequences are well supported, with mean posterior probabilities per site ranging from 0.86 to 0.98 (Table S2). Most of the ambiguously reconstructed sites are in the C-terminus, which is poorly conserved among extant RNH proteins, varies in length, is largely unstructured or missing in crystal structures [27],[28], and can be deleted from ecRNH without affecting function *in vivo* [29]. Most sites outside the C-terminus were reconstructed without ambiguity, and the handful of plausible alternative reconstructions (defined as those having posterior probability >0.3) were virtually all chemically similar to the maximum likelihood reconstruction.

As with all ASR studies, the reconstructed ancestral sequences are statistical approximations rather than certainties: the total posterior probability that the ancestral sequences are precisely correct ranged from 2.6×10^−9^ to 9.8×10^−3^. As discussed below, we addressed this uncertainty in two ways. First, we directly tested the impact of statistical uncertainty on our estimation of *T_m_* by experimentally characterizing ten alternative reconstructions for the deepest node, Anc1. Second, by resurrecting numerous sequences along two diverging lineages and focusing on broad trends rather than the properties of a single ancestor, we minimized the likelihood that statistical error would account for our overall observations.

### Resurrected RNH Proteins Are Folded and Functional

Our first goal was to verify that the reconstructed ancestral proteins behaved like members of the RNH family by characterizing their structural and functional properties. Using far-UV CD, we found that all ancestors are folded at 25°C and exhibit secondary structure consistent with extant RNHs (Figure 2B). We determined the crystal structure of one representative ancestor, AncC, at 1.3 Å resolution (Table S3) and found that it adopts the canonical RNH fold, superimposing with the ecRNH structure with average C_α_ RMSD of 0.8 Å and with the ttRNH structure with an average C_α_ RMSD of 1.3 Å (Figure 2C). Finally, we assayed the reconstructed ancestral proteins for their ability to degrade RNA:DNA hybrids using a simple hyperchromic assay to follow RNA cleavage and nucleotide release [30],[31]: all ancestors were active at 25°C, demonstrating that they are all functional ribonucleases H (Figure S3A).

### *T. thermophilus* and *E. coli* Lineages Exhibit Opposite Trends in Thermostability

**Figure 3:**
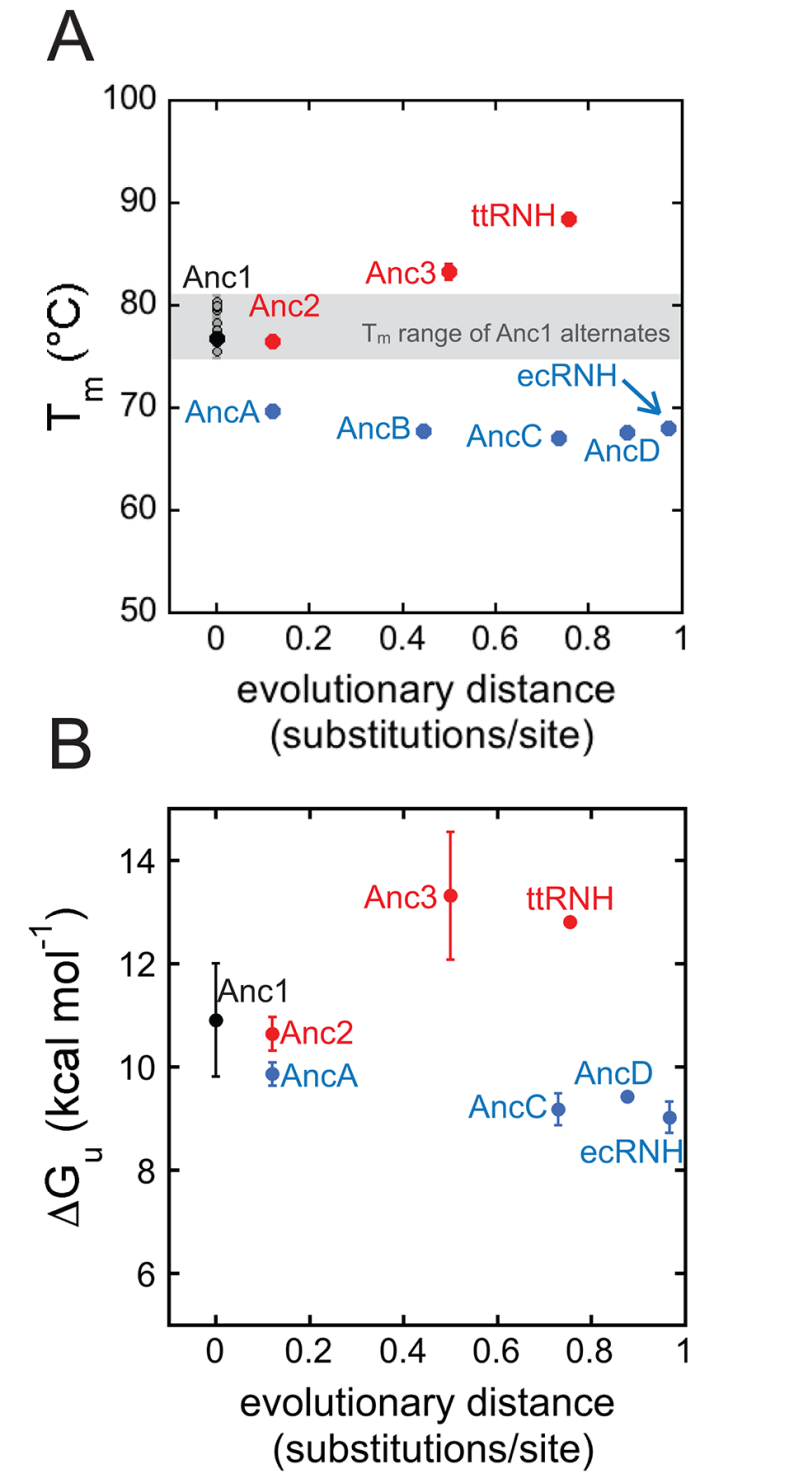
Thermophilic and mesophilic lineages exhibit opposite stability trends. Starting with the shared ancestor, Anc1, stabilities increase along the thermophilic lineage and decrease along the mesophilic lineage. (A) Melting temperature of the maximum likelihood ancestors and the ten alternate reconstructions of Anc1 as a function of evolutionary distance from the last common ancestor, Anc1. Distances are calculated as the sum of the branch lengths connecting Anc1 to the protein of interest. The grey region defines the range of *T_m_*s measured for the Anc1 alternates, which appear individually as grey data points. The error bars are one standard deviation. (B) Average *ΔG*s at 25°C as a function of evolutionary distance from Anc1. Error bars are one standard deviation. See also Table S1 and Dataset S2.

Trends in protein stability over the two evolutionary lineages were determined by measuring the *T_m_*s of the ancestral proteins (Figure S3B). Anc1 has a *T_m_* of 77°C, which is intermediate between ecRNH (68°C) and ttRNH (88°C). Starting from Anc1 and proceeding toward the extant ecRNH and ttRNH, the thermophilic and mesophilic lineages exhibit opposite trends (Figure 3A). *T_m_*s increase along the thermophilic branch and decrease along the mesophilic branch, with ttRNH showing the highest *T_m_* and ecRNH showing second lowest *T_m_*. The temperature-induced unfolding of ancestors along the mesophilic lineage becomes irreversible starting with AncB and continuing through AncC, AncD, and ecRNH. For these proteins, the unfolding curve does not reflect a strictly equilibrium process, so the midpoint of the unfolding curve is therefore only an apparent *T_m_* (Table S1).

To verify that these trends were robust to uncertainty in the ASR, we experimentally characterized ten alternate reconstructions of Anc1. We generated these sequences computationally by randomly sampling at each site in the sequence an amino acid from the posterior distribution of states at that site, excluding implausible states with posterior probabilities <0.2. The resampled ancestors differed from each other and from the maximum likelihood sequence by two to 11 amino acids, with a mean of seven differences. We then synthesized coding DNAs, expressed the proteins, and measured the *T_m_* of each resampled ancestor. The *T_m_* for the ten alternate versions of Anc1 ranged from 75.6°C to 80.5°C with a mean of 78.2°C, comparable to the *T_m_* for the maximum likelihood Anc1 of 76.8°C (Table S1). The range of the phylogenetically plausible *T_m_*s for Anc1 (±1.6°C standard deviation [SD]) is much smaller than the overall changes in *T_m_* along the ecRNH (−9°C) and ttRNH lineages (+12°C) (Figure 3A), indicating that the overall trends in stability are robust to uncertainty in the sequence of Anc1.

### Trends in *ΔG_unf_* at 25°C Mirror Changes in Thermostability

To supplement the thermal melting data, we carried out GdmCl-induced denaturation studies to determine the *ΔG_unf_* at 25°C (Figure S3C; Table S4). All ancestors unfolded reversibly upon chemical denaturation and are well described using the two-state assumption, except AncB, which deviates from two-state behavior and was removed from all further analyses. The trends in *ΔG_unf_* mirror the trend in *T_m_*: *ΔG_unf_* increases by 1.9 kcal mol^−1^ along the thermophilic lineage and decreases by the same amount on the mesophilic lineage (Figure 3B).

### Analysis of the Global Stability Curve Reveals the Mechanism of Stabilization

Our previous work revealed that *ΔC_p_* of ttRNH is lower than that of ecRNH (1.9 versus 2.9 kcal mol^−1^ K^−1^) because it retains residual structure in the unfolded state [32],[33]; this difference contributes 10.7°C to the observed difference in *T_m_* between the two proteins. This stabilization mechanism might be adaptive, allowing the protein to achieve a high *T_m_* while maintaining conformational flexibility and a moderate *ΔG* at its growth temperature; residual structure in the unfolded state could also help minimize aggregation under unfolding conditions [5],[17],[33]. We therefore hypothesized that the evolution of residual structure in the unfolded state was the mechanism by which adaptive thermostability evolved along the thermophilic lineage.

To test this hypothesis and elucidate the mechanisms by which stability was tuned along these lineages, we determined the global protein stability curve for each ancestor (Figure S4). This curve, a plot of *ΔG_unf_* against temperature, can be determined by performing chemical denaturation experiments across a wide range of temperatures. It exhibits a characteristic parabolic shape that is described by the Gibbs-Helmholtz equation:
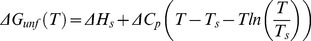
(1)


**Figure 4:**
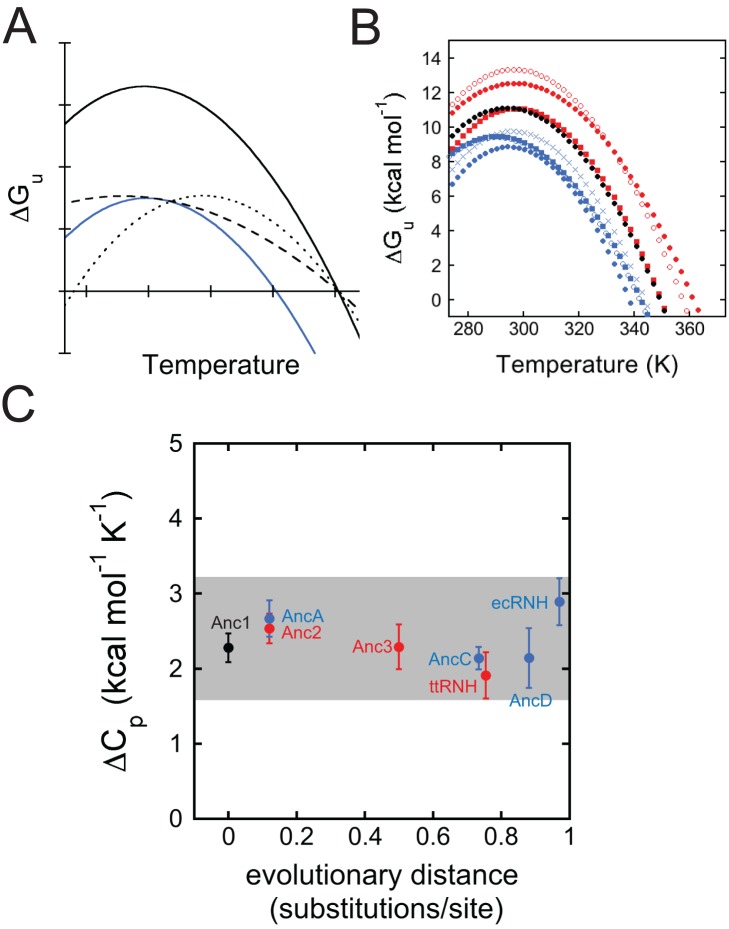
All possible mechanisms for manipulating *T_m_* are represented in RNH's evolutionary history. (A) Thermodynamic strategies for increasing *T_m_*. Relative to the reference state (blue line), the stability curve can be upshifted (solid black line), broadened (dashed line), or right-shifted (dotted line). (B) Superimposed stability curve fits for ecRNH (blue solid circles), ttRNH (red solid circles), Anc1 (black solid circles), ancestors from the mesophilic lineage (AncA, blue Xs; AncC, blue solid squares; AncD blue open circles), and ancestors from the thermophilic lineage (Anc2, red solid squares; Anc3, red open circles). (C) *ΔC_p_* as function of evolutionary distance from Anc1. See also Dataset S2.

This equation defines the temperature-dependent relationship between the global stability of a protein (*ΔG_unf_*) and three key thermodynamic parameters: the temperature of maximum stability (*T_s_*), the change in enthalpy upon unfolding at *T_s_* (*ΔH_s_*), and the change in heat capacity upon unfolding (*ΔC_p_*). The *T_m_*, or thermal melting temperature, is the right-most *x*-intercept of this curve. By fitting the parameters of the equation to our data, we can extract detailed thermodynamic and mechanistic information about each ancestor (Figure 4B; Table S5), including evidence for residual structure in the denatured state. Then, by looking at trends in these parameters over evolutionary time, we can identify changes that led to the evolution of thermostability.

Modification of each of the three thermodynamic parameters corresponds to a specific mechanism of stabilization and can be understood graphically as altering the features of the curve to increase the *T_m_* (Figure 4A). First, increasing *ΔH_s_* causes the curve to move upwards along the *ΔG* axis; this is easily achieved by adding favorable interactions in the folded state. Such changes in enthalpy are a common basis for change in stability upon mutation, as small changes in side-chain functional groups can result in large changes in native-state interactions. Second, depressing *ΔC_p_* broadens the stability curve, increasing *ΔG* at temperatures above or below *T*_s_. *ΔC_p_* depends largely on the difference in protein-water interactions between the unfolded and folded states; a variant that creates residual structure in the unfolded state will decrease the difference in protein-water interactions between the unfolded and folded states and thereby decrease *ΔC_p_*. Third, increasing *T_s_* moves the curve to the right along the *x-*axis. *T_s_* is relatively insensitive to mutations and is therefore not considered a common mechanism of stabilization [34].

### Different Ancestors Use Different Mechanisms of Stabilization

All of the ancestors have *ΔC_p_*s falling within a narrow range (Figure 4C). To test the hypothesis that changes in the heat capacity of unfolding caused the gradual increase of *T_m_* along the thermophile lineage, we carried out two different analyses. First, we analyzed the results from our fits of the stability curve for each ancestor (Figure S4; Table S5) to quantify the relative contribution of *ΔC_p_*, *T_s_*, and *ΔH_s_* to changes in *T_m_* relative to Anc1. We assessed the change in *T_m_* predicted when the measured parameters for each extant or ancestral RNH protein were individually substituted into the Gibbs-Helmholtz equation with all other parameters from Anc1. This analysis uses Anc1 as a reference state and assumes that the effects of each parameter can be assessed independently.

**Figure 5:**
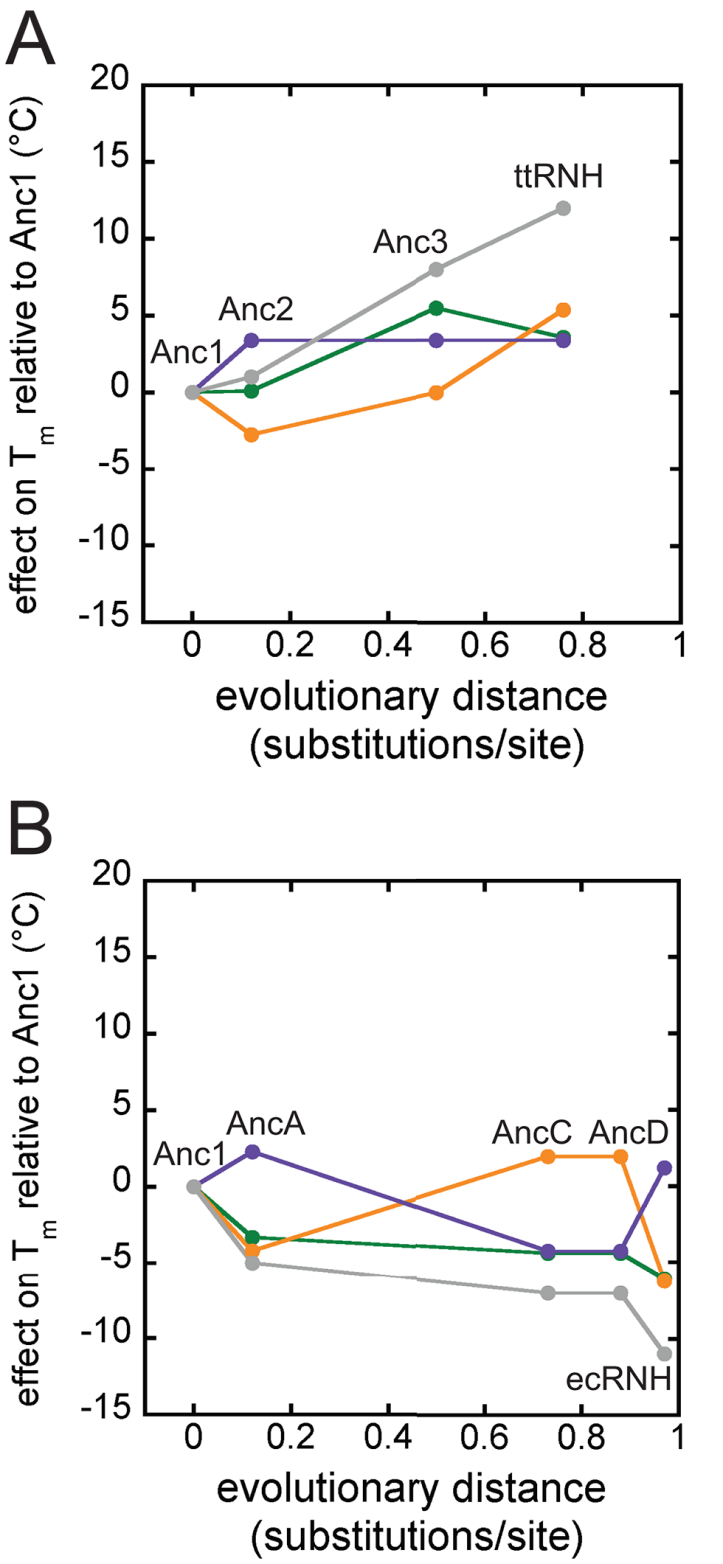
No single parameter dominates changes in thermostability along the RNH lineages. Figure shows the change in *T_m_* and contribution of each thermodynamic parameter to *T_m_* for the maximum likelihood evolutionary path. (A) How each thermodynamic parameter contributes to changes in *T_m_* along the thermophilic lineage. Total changes in *T_m_* (grey) represent the sum of contributions from *ΔC_p_* (orange), *ΔH_s_* (green), and *ΔT_s_* (purple). (B) How each thermodynamic parameter contributes to changes in *T_m_* along the mesophilic lineage. Colors are as in (A). See also Dataset S2.

Our hypothesis predicts that *ΔC_p_* should decline consistently along the thermophilic lineage from Anc1 to ttRNH in a fashion correlated with the evolutionary increase in *T_m_*. As expected, *ΔC_p_* of ttRNH is lower than that of Anc1 (2.28±0.2 and 1.91±0.3 kcal mol^−1^ K^−1^, respectively). The elevated *T_m_* of ttRNH, however, is caused not only by changes in *ΔC_p_* but also by substantial changes in *T_s_* and *ΔH_s_*; whereas changes in *ΔC_p_* caused an increase in *T_m_* of 5.4°C, changes in *ΔH_s_* and *T_s_* caused additional increases of 3.6°C and 3.4°C, respectively (Figure 5A).

Contrary to our prediction, however, the observed trends in *T_m_* are not mirrored by trends in *ΔC_p_* (Figure 5A), and the underpinnings of the *T_m_* of each ancestor are different. The *T_m_* of ttRNH is 12°C above Anc1's because of contributions from *ΔC_p_* (+5.4°C), *ΔH_s_* (+3.6°C), and *T_s_* (+3.4°C). In contrast, although the *T_m_* of Anc3 is 8°C higher than Anc1, *ΔC_p_* makes no contribution at all to its elevated thermostability, which is instead driven by *ΔH_s_* (+5.5°C) and *T_s_* (+3.5°C). Anc2 exhibits yet another pattern: its *T_m_* is 1°C higher than Anc1 due to a strong contribution by *T_s_* (3.7°C), which offsets a destabilizing effect of *ΔC_p_* (−2.7°C), while *ΔH_s_* makes virtually no contribution. Thus, although *T_m_* consistently increases along the thermophilic lineage, our analysis suggests that different stabilizing mechanisms are utilized between each ancestor.

The mechanisms altering thermostability fluctuate along the mesophilic lineage, as well (Figure 5B). The *T_m_* of the modern mesophilic ecRNH is 11°C lower than that of Anc1. This reflects major unfavorable contributions from *ΔH_s_* (−6.1°C) and *ΔC_p_* (−6.2°C), with a minor stabilizing effect by *T_s_* (+1.1°C). As with the thermophilic lineage, however, the contributions of changes in *ΔC_p_*, *ΔH_s_*, and *T_s_* fluctuate over evolutionary time. AncC and AncD have nearly identical thermostabilities, with *T_m_*s ∼7°C lower than Anc1 because of unfavorable changes in *ΔH_s_* (−4.4°C) and *T_s_* (−4.3°C) and a favorable change in *ΔC_p_* (+1.2°C). In contrast, the *T_m_* of AncA is 5°C lower than Anc1, but *ΔC_p_* (−4.2°C) and *T_s_* (+1.9°C) have now switched roles, with *ΔH_s_* (−3.3°C) continuing to be destabilizing.

**Figure 6:**
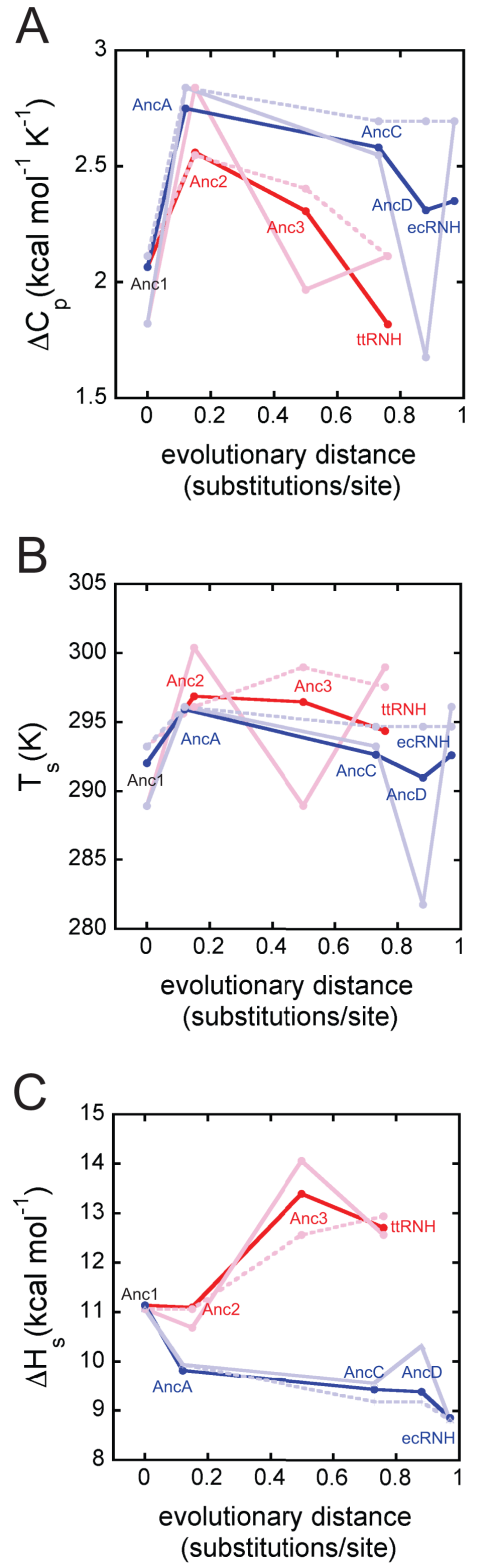
The thermodynamic underpinnings of RNH *T_m_* fluctuated over evolutionary time. Estimated *ΔC_p_* (A), *T_s_* (B), and *ΔH_s_* (C) over the evolution of ttRNH (red) and ecRNH (blue). Maximum likelihood path is shown in bold. The shortest (dashed line) and longest (solid line) plausible alternate trajectories (95% confidence interval) are shown in faded blue and pink. See also Dataset S2.

We statistically analyzed whether the apparent fluctuations in the mechanistic underpinnings of stability over evolutionary time were robust to uncertainty in the estimates of the parameters of the Gibbs-Helmholtz equation. We used a bootstrap and refitting approach to identify for each ancestor a cloud of plausible parameter values that are consistent with the experimental data (Figures S6 and S7). We found that the thermodynamic underpinnings of the ancestral proteins' stabilities are indeed distinct from each other (Figure S7; Table S6). We then asked about the possible evolutionary trajectories along phylogenetic lineages given uncertainty in the parameter estimates. We discretized the parameter space for each ancestral protein and calculated from the refitted bootstrap parameter estimates the probability that the ancestral protein was in each region of this parameter space (see Materials and Methods). We then calculated the probability of each possible pathway through this space that could be taken by lineages of successive ancestors on the phylogenetic tree, given the bootstrap-derived probability that each ancestor's parameters were in each parameter region. We found that, along both lineages, the most probable path was circuitous, with values of *T_s_* and *ΔC_p_* in particular fluctuating across the evolutionary intervals (Figure 6A and 6B). This stands in contrast with the value of *T_m_*, which increases smoothly on the thermophile lineage and, after an early drop, remains essentially constant along the mesophilic lineage (Figure 3A). We also investigated the distribution of alternative possible paths (Figure S8). Paths within the 95% confidence interval had lengths between 75% and 230% of the length of the most probable path. Even the shortest plausible path, at the very edge of the confidence interval, still exhibited dramatic fluctuations over evolutionary time (Figure 6).

Thus, our observed experimental data and the bootstrapped refitting both indicate that the mechanistic underpinnings of stability did not follow the smooth evolutionary trajectories followed by stability itself but instead fluctuated dramatically during the evolution of thermophilic and mesophilic RNH proteins. The parameter that appears to be most consistently associated with changes in *T_m_* along both lineages is *ΔH_s_*, with the noted exception of ttRNH (Figure 6C). Changes in *ΔH_s_* can be achieved simply by manipulating side-chain interactions in the folded state. This is the most commonly observed mechanism in thermophilic proteins [5] and is the one most often exploited for engineering protein stability [35]. Despite this general correlation, however, it is clear that no singular mechanism dominates along either lineage.

## Discussion

### Current Mechanisms for Thermostability Differ from Historical Ones

In this study, we used ASR to study the evolution of thermostability of RNH enzymes along mesophilic (ecRNH) and thermophilic (ttRNH) lineages. We observed that, while *T_m_* changed smoothly over both lineages, the mechanism of stabilization followed a more tortuous path. This was particularly surprising for *ΔC_p_*, which is the most striking thermodynamic difference between ecRNH and ttRNH, accounting for a full 10.7°C difference in *T_m_*. As such, we hypothesized that a depressed *ΔC_p_* would be the primary mechanism of stabilization. The fluctuations observed in the mechanistic underpinnings of function along both lineages indicate that this parameter, and the changes in residual unfolded structure that produce it, are not driven by the same selective pressures imposed on *T_m_*. Further evidence for the de-coupling of *ΔC_p_* and *T_m_* comes from the modern RNH homolog from *Chlorobium tepidum*, which has a mesophile-like *T_m_* of 66.5°C and a thermophile-like *ΔC_p_* of 1.7 kcal mol^−1^ K^−1^ [21].

### Implications for Inferences of Ancient Environmental Temperature

Our findings concerning trends in *T_m_*s bear on recent efforts to infer long-term changes in global environmental temperature based on studies of resurrected ancestral proteins. We observed that the RNH of the ancestor of thermophilic and mesophilic bacteria had a *T_m_* intermediate between those of extant members of the two groups, with a gradual progression to higher *T_m_*s along the thermophilic lineage and an initially abrupt evolution to lower *T_m_* s on the mesophilic lineage followed by little long-term change. These results differ from studies of several other proteins, which observed monotonic increases in *T_m_* as one goes back in time and interpreted this result as evidence for an ancient, global trend from higher to lower environmental temperatures [8]–[10],[12],[13]. Our oldest ancestor, Anc1, has a *T_m_* 10°C higher than extant mesophiles and 10°C lower than extant thermophiles, in contrast to similarly aged thioredoxins and β-lactamases, which were found to have *T_m_*s 25–30°C higher than both their mesophilic and thermophilic descendants [12],[13]. Our findings are more similar to those of a study showing that ancestral EF-Tu proteins have thermostabilities between those of extant thermophiles and mesophiles, a result interpreted as providing evidence for a relatively hot ancient global environment [9]. By tracing changes in *T_m_* along multiple taxonomic lineages, we found patterns that are inconsistent with any gradual, long-term trend in global environmental temperatures: for example, Anc2, which is estimated to have existed approximately 2 billion years ago [26], has a melting temperature only 2°C higher than that of present-day mesophilic ecRNH. Taken together, our observations suggest that particular proteins in particular lineages undergo their own paths to accommodate the local environments they colonize and the functions they perform. This view is consistent with the wide variety of temperature niches populated by both ancient and modern bacteria. It is also consistent with recent findings that thermophilicity evolved in parallel numerous times over a period of just 3 million years in a family of enzymes involved in leucine biosynthesis [11]. We therefore suggest that the tracking the *T_m_* of any individual protein or lineage is an unreliable way to estimate long-term trends in global environmental temperatures.

### RNH Exhibits Thermodynamic Systems Drift

Our work reveals that RNH thermostability is evolving in a regime analogous to “developmental systems drift” (DSD). In DSD, a developmental process or outcome is conserved by selection, but the underlying genetic or molecular mechanisms shift during evolution [36]. By analogy, RNH exhibits thermodynamic systems drift, in which the *T_m_* is under selection, but the mechanisms by which it is achieved vary over evolutionary time. “Drift,” in this usage, does not refer to the evolutionary process of neutral drift, but rather to changes in mechanism uncorrelated with changes in phenotype.

Although our data do not directly reveal the evolutionary forces that shape the mechanism of stabilization, the most parsimonious explanation of our observations is a neutral evolutionary process. A protein's stability must be above a given threshold to maintain protein function and prevent the accumulation of misfolded protein [37],[38], but there is little evidence that selection can “sense” the underlying mechanism of stabilization, implying that neutral evolution would be free to alter stabilization mechanisms while maintaining the required stability. Further, invoking selection to explain the fluctuations we observed in the thermodynamic parameters that determine stability requires that the each ancestor experienced a unique selective regime and that this regime was different from that driving stability itself (Figure 1). We believe that this scenario, although formally possible, is very unlikely.

Thermodynamic systems drift has some important implications. First, it highlights the pitfalls of proposing an adaptive trajectory based on comparisons of modern proteins. Almost all studies of thermostability have compared mesophilic and thermophilic homologs [5]; our work indicates that mechanistic differences between any two such proteins—even those that strongly correlate with stability—may reflect exploration of alternate stabilization mechanisms rather than the initial adaptive stabilization mechanism. A second implication is that studying multiple thermophilic homologs—or an evolutionary lineage leading to a thermophilic protein—has the capacity to reveal multiple methods of stabilization for a given protein, thus providing insight for engineers trying to identify stabilizing mutations by studying protein diversity. Finally, thermodynamic systems drift implies that evolving proteins can efficiently explore sequence space, even when selection establishes a threshold for stability [39]. As proteins explore sequence space, their evolutionary potential changes, opening and closing pathways to new functions or properties [39]–[42].

## Materials and Methods

### Ancestral Sequence Reconstruction

Bacterial and archaeal RNH sequences were identified by BLAST against the NCBI non-redundant protein database using RNH from *E. coli* and *T. thermophilus* sequences as seed sequences [43],[44]. Redundant sequences were removed using cdhit 4.6 [45]. In total, 409 sequences were kept for further analysis. Sequences were aligned using MUSCLE 3.8.31, followed by manual refinement using Mesquite 2.75 (Maddison and Maddison). Alignment quality was verified by checking for alignment of three universally conserved acidic residues that compose the RNH active site (Figure S1). The final alignment is available in Dataset S1. The maximum likelihood phylogenetic tree was constructed using the JTT+Γ_8_ substitution model—identified by ProtTest [46] —and SPR moves as implemented in PhyML 3.0 [47],[48]. Branch supports were estimated using the approximate likelihood ratio test [49]. Maximum likelihood ancestral RNH sequences were reconstructed with the maximum likelihood topology, branch lengths, and phylogenetic model using PAML 3.14 [50],[51].

### Expression and Purification

Genes encoding the ancestral proteins were codon optimized for expression in *E. coli* and synthesized by GENEART. The genes were subcloned using NdeI and HindIII restriction sites into the multiple cloning site of a pET27 vector (Life Technologies). Other site-specific variants were constructed via site-directed mutagenesis and verified by sequencing.

Plasmids were transformed into BL21(DE3) pLysS cells for expression. Cells were induced with 1 mM IPTG at OD = 0.6 and grown at 37°C for 3 hours before harvesting. Cells were lysed in buffer via sonication. All ancestors expressed predominantly in the soluble fraction, though some partitioned into inclusion bodies as well. Only solubly expressed proteins were purified for further analysis. Lysate was purified first over a HiTrap Heparin column (GE Healthcare) at pH 8. Peak fractions were pooled and diluted 2-fold with doubly deionized water. Then the pH of the solution was adjusted to 5.5 using dilute NaOAc, and the sample was purified over a HiTrap S column (GE Healthcare). Protein was then concentrated and dialyzed against either ammonium bicarbonate for subsequent freeze-drying and storage or appropriate buffer conditions for immediate use. Each protein's purity and molecular weight were confirmed by SDS-PAGE and electrospray mass spectrometry. Protein concentrations were determined in Edelhoch buffer using extinction coefficients calculated based on the number of tryptophan and tyrosine residues [52].

### Circular dichroism Spectroscopy

CD spectra were collected on an AVIV 410 spectrophotometer using protein samples at 0.5 mg/ml (20 mM NaOAc [pH 5.5], 50 mM KCl, and 1 mM TCEP) in a 0.1 cm quartz cuvette at 25°C. Data points were collected from 250–200 nm at 1-nm intervals, and each data point represents signal averaged over 5 seconds.

### Crystallization and Structure Determination of AncC

Crystals were grown at 18°C in hanging drop format by mixing 1 µl protein solution with 1 µl well solution containing 20% PEG 3350, 20–50 mM Li_2_SO_4_, 1 mM TCEP, and 100 mM Bis-tris (pH 6.5). For harvesting, crystals were transferred for 1 minute to well solution containing 10% glycerol for cryoprotection, and then looped and flash frozen in liquid nitrogen. Data were collected at Beamline 8.3.1 (wavelength 1.1159 Å) under a cryo-stream at the Advanced Light Source (ALS) at Lawrence Berkeley National Laboratory, and integrated using HKL2000 [53]. Initial phases were calculated by molecular replacement (MR) using PHASER [54]. The search model was the extant RNH from *E. coli* (PDB ID: 2RN2). Building of the model was carried out in COOT [55], followed by a refinement strategy using PHENIX [56] that consisted of an initial round of rigid-body refinement, followed by individual-atom positional and anisotropic ADP refinement including hydrogens. Structure validation was assisted by MolProbity [57], and figures were rendered using PyMOL [58]. The structure was deposited in the Protein Data Bank (PDB ID: 4LY7).

### Activity Assay

RNH activity was assayed in 50 mM NaCl, 10 mM Tris (pH 8.0), 10 mM MgCl_2_, 1 mM TCEP at 25°C. Substrate concentration is given in internucletotide bonds, due to the heterogeneous nature of the substrate, using ε_260_ = 8250 M^−1^ cm^−1^ and 330 g/mol for the average nucleotide molecular weight. Substrate was prepared by mixing equal parts dT_20_ oligomers (IDT) and poly-rA (Sigma), heating to 95°C for 5 minutes, then slowly cooling to room temperature for 1 hour before storing at 4°C. The reaction was initiated by the addition of enzyme and monitored at 260 nm using a Cary UV spectrophotometer. Increasing absorbance at 260 nm indicates the release of nucleotides as they are hydrolyzed.

### Denaturant-Induced and Thermal Denaturation

Thermal and chemical denaturation melts were performed by monitoring the CD signal at 222. CD melts were carried out in a 1-cm pathlength cuvette (50 µg/ml protein, 20 mM NaOAc [pH 5.5], 50 mM KCl, and 1 mM TCEP). Samples were prepared individually, equilibrated overnight or longer for temperatures below 25°C, and allowed to stir in the instrument for 1–2 minutes before data collection. For higher temperatures, proteins were equilibrated overnight only if solubility was not compromised. Otherwise, shorter equilibration times were used, typically 2–3 hours for melts performed manually and 5–15 minutes for melts performed using an automated titrator. Data from titration experiments were only used if the measured *C_m_* was within 0.1 M of the value collected manually at the same temperature.

To measure CD signal at 222 nm as a function of temperature, samples were allowed to equilibrate for five minutes at each temperature and data were collected every 3°C. Spectra were taken at 25°C before and after the thermal melt to test for reversibility. Reversibility was defined as recovery of 80% of CD signal at 222 nm. Temperature melts were fit to a two-state model using the Gibbs-Helmholtz relationship (Equation 1).

### Denaturation and Stability Curve Data Analysis

To generate stability curves, global stabilities derived from GdmCl-induced denaturation melts were plotted as a function of temperature. *T_m_*s extracted from thermal denaturation experiments were used as single points at *ΔG* = 0 for ttRNH, AncA, Anc1, Anc2, and Anc3, which all unfold reversibly. Only data collected at temperatures above 15°C were included in fits due to deviation from two-state behavior at lower temperatures (Figure S5). Data were fit to the Gibbs-Helmholtz equation to extract all thermodynamic parameters (Equation 1).

To characterize the uncertainty in our estimates of the thermodynamic parameters, we generated 10,000 bootstrap pseudo-datasets for each protein—sampling three times with replacement from the measured *ΔG* values at each temperature—and then refit our model to each dataset (Figure S6). This generated a distribution of parameter values consistent with the experimental measurements taken for each protein. To characterize uncertainty in the trends that parameters followed along phylogenetic lineages, we sampled possible pathways between the bootstrapped parameter values of successive ancestors along the tree and then characterized the lengths and probabilities of these pathways. We first placed all three parameters on the same scale by normalizing their values to the interval (0, 1). Next, we discretized the parameter space into a 15×15×15 grid, reducing the 10,000 unique parameter estimates for each protein to ∼35 unique estimates. We calculated the relative probability of each grid cell, given our experimental data, as the proportion of all pseudo-datasets with parameter estimates in that cell. Finally, we exhaustively sampled trajectories among successive ancestral proteins through this parameter space. For each trajectory, we calculated the path length (the sum of Euclidean distances between successive ancestor's parameter values) and its probability (determined by multiplying the probabilities of each bin in the path). Statistical analyses were done in the R 3.1.0 statistical environment [59]. Fittings was done using the L-BFGS-B algorithm as implemented in the optim function [60].

## Supporting Information

Figure S1 **WebLogo representation of the RNH multiple sequence alignment** [61] **.** Conservation is reflected by the overall height of the stack at each position. Height of individual letters within the stack indicates the relative frequency of a residue at the position. Numbering and secondary structure elements are based on ecRNH. Active site residues are starred. See also Dataset S1.
(TIF)

Figure S2 **RNH phylogenetic tree and sequence comparisons.** (A) Rooting does not change the relative relationships between ecRNH, ttRNH, and the ancestors. ASR was performed using an unrooted tree built from an alignment of 409 RNH sequences. An additional 45 archaeal RNH sequences were used to create the rooted tree, which allows ordering of the ancestors in time. Branch length reflects sequence distance, as indicated by the scale bar, in average number of substitutions per position. Resurrected nodes are starred (see Table S2). Branch supports for the trees are labeled. (B) Alignment of ancestors with ecRNH and ttRNH. Secondary structure elements are based on ecRNH. (C) Sequence identity matrix for ancestors, ecRNH, and ttRNH. Ancestors that are analogously spaced along the thermophilic and mesophilic lineages appear in the same color. See also Datasets S3 and S4.
(TIF)

Figure S3 **Measuring activity and stability of ancestors, ecRNH, and ttRNH.** (A) Activity at 25°C in 10 mM Tris (pH 8), 50 mM NaCl, 10 mM MgCl_2_, 1 mM TCEP, and 16.7 µg/ml poly-rA:dT_20_ substrate for ecRNH (blue solid circles), ttRNH (red solid circles), Anc1 (black solid circles), ancestors from the mesophilic lineage (AncA, blue Xs; AncB, blue open squares; AncC, blue solid squares; AncD blue open circles), and ancestors from the thermophilic lineage (Anc2, red solid squares; Anc3, red open circles). (B) Thermal denaturation as probed by CD signal at 222 nm. (C) Chemical denaturation at 25°C as monitored by CD at 222 nm. See also Dataset S2.
(TIF)

Figure S4 **Stability curves.** (A) Anc1, (B) Anc2, (C) Anc3, (D) AncA, (E) AncC, (F) AncD, (G) ecRNH, (H) ttRNH. Average *ΔG* values measured at 15°C or higher were used for the fits, and errors are standard deviations from fits of replicate experiments. See also Dataset S2.
(TIF)

Figure S5 **Deviation from two-state behavior at low temperatures.** (A) *ΔG* values of ecRNH unexpectedly plateau below 15°C. Data at 5°C and 10°C reflect the averages of 12 and seven independent experiments, respectively. The displayed fit does not include data below 15°C. (B) Stability curve fit from the cysteine-free variant ecRNH C13A/C63A/C133A (black curve) superimposed with data from single cysteine variants (ecRNH C13A/C133A, orange closed circles; ecRNH C13A/C63A, orange open circles; ecRNH C133A/C63A, orange Xs). Asymmetry in ecRNH stability data is due to C63. See also **Dataset S2**.
(TIF)

Figure S6 **Global fits to bootstrap samples of stability versus temperature curves of different RNH proteins.** Points show *ΔG_unf_* values measured for each protein as a function of temperature. Lines show a random sample of 100 re-fits of these data generated by bootstrap sampling. Colors denote different proteins: Anc1 (black), Anc2 (wheat), Anc3 (orange), ttRNH (red), AncA (light blue), AncC (dark green), AncD (slate), and ecRNH (blue). Arrows show the maximum likelihood trajectory through this space, starting from Anc1 and going to ttRNH (red path) or starting from Anc1 and going to ecRNH (blue).
(TIF)

Figure S7 **No smooth pathway exists through possible ancestral parameter space.** Plots projection of 3D plot of *ΔC_p_* versus *T_s_* versus *ΔH_s_* for each protein. Points show fit parameters extracted from bootstrap replicates. Colors denote different proteins: Anc1 (black), Anc2 (wheat), Anc3 (orange), ttRNH (red), AncA (light blue), AncC (dark green), AncD (slate), and ecRNH (blue). Arrows show the maximum likelihood trajectory through this space, starting from Anc1 and going to ttRNH (red path) or starting from Anc1 and going to ecRNH (blue).
(TIF)

Figure S8 **Distribution of all possible path lengths reveals that 95% of possible paths are nearly as long as the ML path.** Histograms of possible path lengths weighted by path likelihoods, extracted from explicit enumeration of pathways through the parameter space. Path lengths are normalized to the maximum likelihood path. Red and blue curves denote the thermophilic and mesophilic lineages, respectively. Dashed lines indicate 95% cutoff.
(TIF)

Table S1 **Growth temperatures,** ***T_m_*** **s and** ***ΔG*** **s at** ***T_env_*** **for extant and ancestral RNH proteins.** ^*^Extracted from stability curve fits for two-state proteins. ^†^Errors reported are standard deviations from replicate experiments. ^‡^Taken from reference [20]. ^§^Taken from reference [21]. ^¶^Not determined.
(DOCX)

Table S2 **Statistical support for resurrected ancestors.**
(DOCX)

Table S3 **Data collection and refinement statistics for AncC.** ^*^Values in parentheses are for highest-resolution shell.
(DOCX)

Table S4 ***ΔG*** **s and** ***m*** **-values at 25°C.** *Errors reported are standard deviations from replicate experiments.
(DOCX)

Table S5 **Thermodynamic parameters from stability curve fits.** * Errors from fit. † Extracted from thermal melt fit. ‡ Extracted from stability curve fit.
(DOCX)

Table S6 **Individual proteins populate discrete regions of parameter space.**
(DOCX)

Dataset S1 **RNH sequence alignment.**
(DOCX)

Dataset S2 **Numerical data underlying main text and supplemental figures.**
(XLSX)

Dataset S3 **Phylogenetic tree file used to generate the unrooted tree in Figure S2.** Tree files are in the standard “newick” ascii text format and can be opened via a wide variety of freely available and commercial tree-viewing programs. Two free programs are FigTree (http://tree.bio.ed.ac.uk/software/figtree/) and archaeopteryx (https://sites.google.com/site/cmzmasek/home/software/archaeopteryx).
(TXT)

Dataset S4 **Phylogenetic tree file used to generate the rooted tree in Figure S2.** Tree files are in the standard “newick” ascii text format and can be opened via a wide variety of freely available and commercial tree-viewing programs. Two free programs are FigTree (http://tree.bio.ed.ac.uk/software/figtree/) and archaeopteryx (https://sites.google.com/site/cmzmasek/home/software/archaeopteryx).
(TXT)

## Abbreviations

ASR: ancestral sequence reconstruction
CD: circular dichroism
ecRNH: *E. coli* ribonuclease H1
RNH: ribonuclease H1
ttRNH: *T. thermophilus* ribonuclease H1
