# Correction: Thermodynamic System Drift in Protein Evolution

**DOI:** 10.1371/journal.pbio.1002091

**Published:** 2015-02-18

**Authors:** The PLOS Biology Staff

At the time of publication, the ancestral sequence data from this paper had not been deposited. The ancestral sequences shown in S2B Fig. are now available in GenBank (KP271037—KP271043). An easily accessible sequence alignment file for S2B Fig. is now also available (S1 File).

## Supporting Information

S1 File RNase H reconstructed DNA sequences. (TXT)
