# Supplementary figures and images for: Thermodynamic System Drift in Protein Evolution
Source: PLoS Biol. 2014 Nov 11;12(11):e1001994. doi: 10.1371/journal.pbio.1001994 (PMC4227636; doi:10.1371/journal.pbio.1001994)

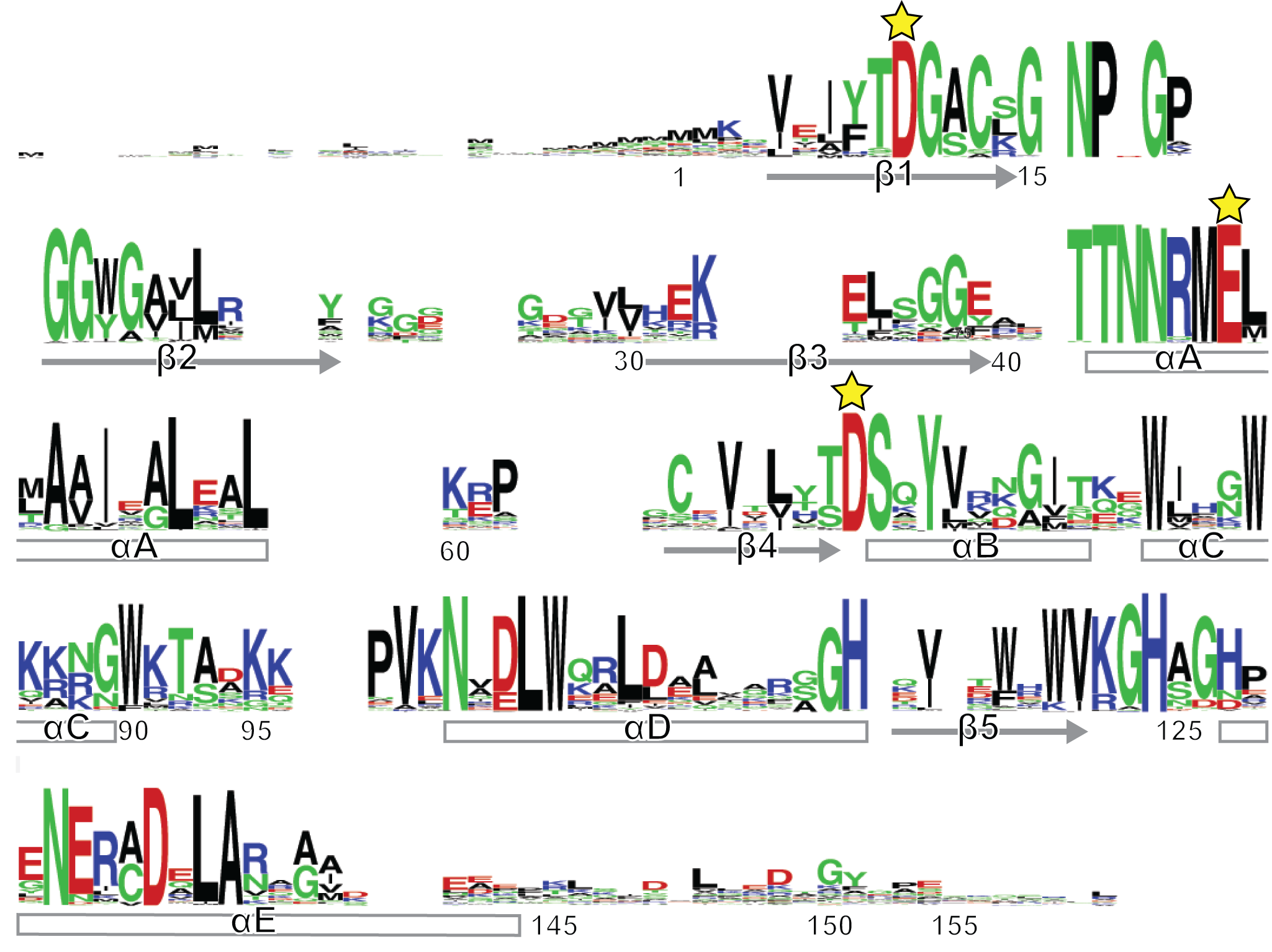

Supplement: Figure S1 — WebLogo representation of the RNH multiple sequence alignment [61] . Conservation is reflected by the overall height of the stack at each position. Height of individual letters within the stack indicates the relative frequency of a residue at the position. Numbering and secondary structure elements are based on ecRNH. Active site residues are starred. See also Dataset S1. (TIF) [file pbio.1001994.s001.tif]

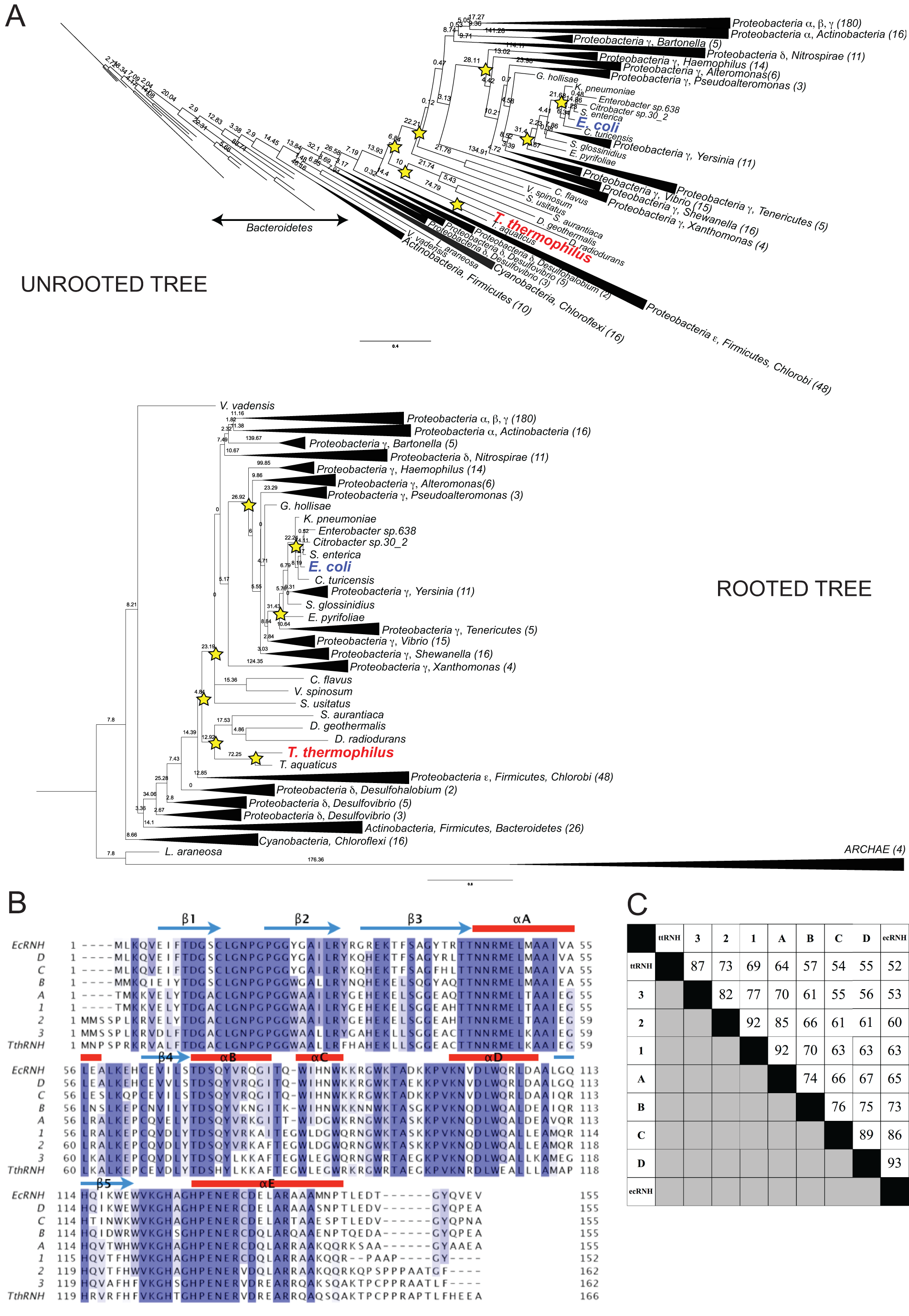

Supplement: Figure S2 — RNH phylogenetic tree and sequence comparisons. (A) Rooting does not change the relative relationships between ecRNH, ttRNH, and the ancestors. ASR was performed using an unrooted tree built from an alignment of 409 RNH sequences. An additional 45 archaeal RNH sequences were used to create the rooted tree, which allows ordering of the ancestors in time. Branch length reflects sequence distance, as indicated by the scale bar, in average number of substitutions per position. Resurrected nodes are starred (see Table S2). Branch supports for the trees are labeled. (B) Alignment of ancestors with ecRNH and ttRNH. Secondary structure elements are based on ecRNH. (C) Sequence identity matrix for ancestors, ecRNH, and ttRNH. Ancestors that are analogously spaced along the thermophilic and mesophilic lineages appear in the same color. See also Datasets S3 and S4. (TIF) [file pbio.1001994.s002.tif]

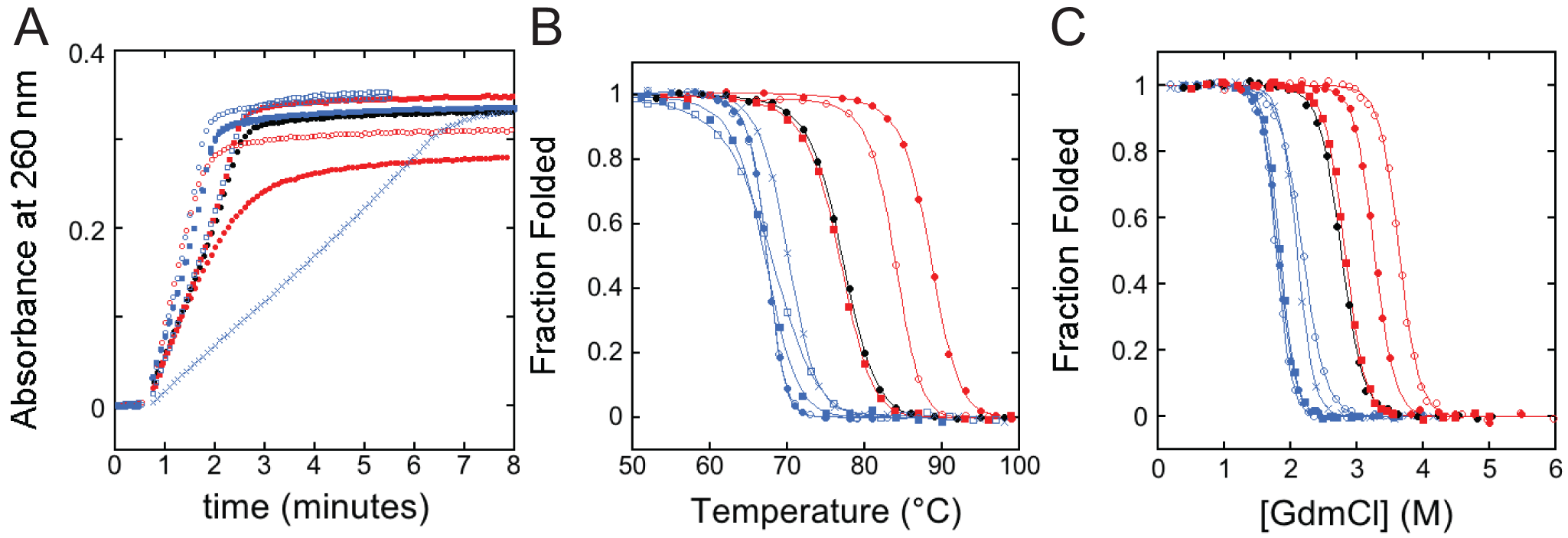

Supplement: Figure S3 — Measuring activity and stability of ancestors, ecRNH, and ttRNH. (A) Activity at 25°C in 10 mM Tris (pH 8), 50 mM NaCl, 10 mM MgCl2, 1 mM TCEP, and 16.7 µg/ml poly-rA:dT20 substrate for ecRNH (blue solid circles), ttRNH (red solid circles), Anc1 (black solid circles), ancestors from the mesophilic lineage (AncA, blue Xs; AncB, blue open squares; AncC, blue solid squares; AncD blue open circles), and ancestors from the thermophilic lineage (Anc2, red solid squares; Anc3, red open circles). (B) Thermal denaturation as probed by CD signal at 222 nm. (C) Chemical denaturation at 25°C as monitored by CD at 222 nm. See also Dataset S2. (TIF) [file pbio.1001994.s003.tif]

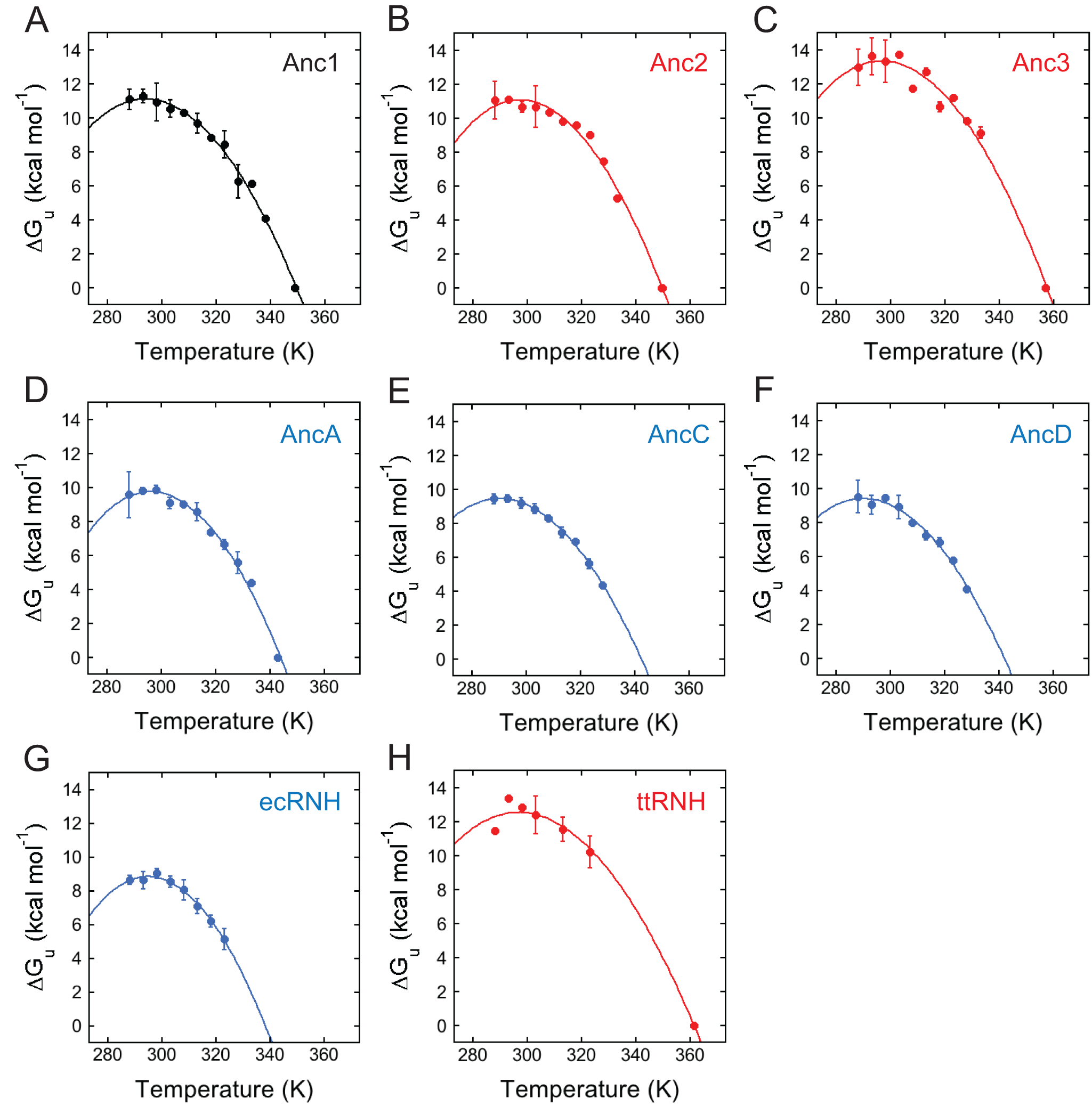

Supplement: Figure S4 — Stability curves. (A) Anc1, (B) Anc2, (C) Anc3, (D) AncA, (E) AncC, (F) AncD, (G) ecRNH, (H) ttRNH. Average ΔG values measured at 15°C or higher were used for the fits, and errors are standard deviations from fits of replicate experiments. See also Dataset S2. (TIF) [file pbio.1001994.s004.tif]

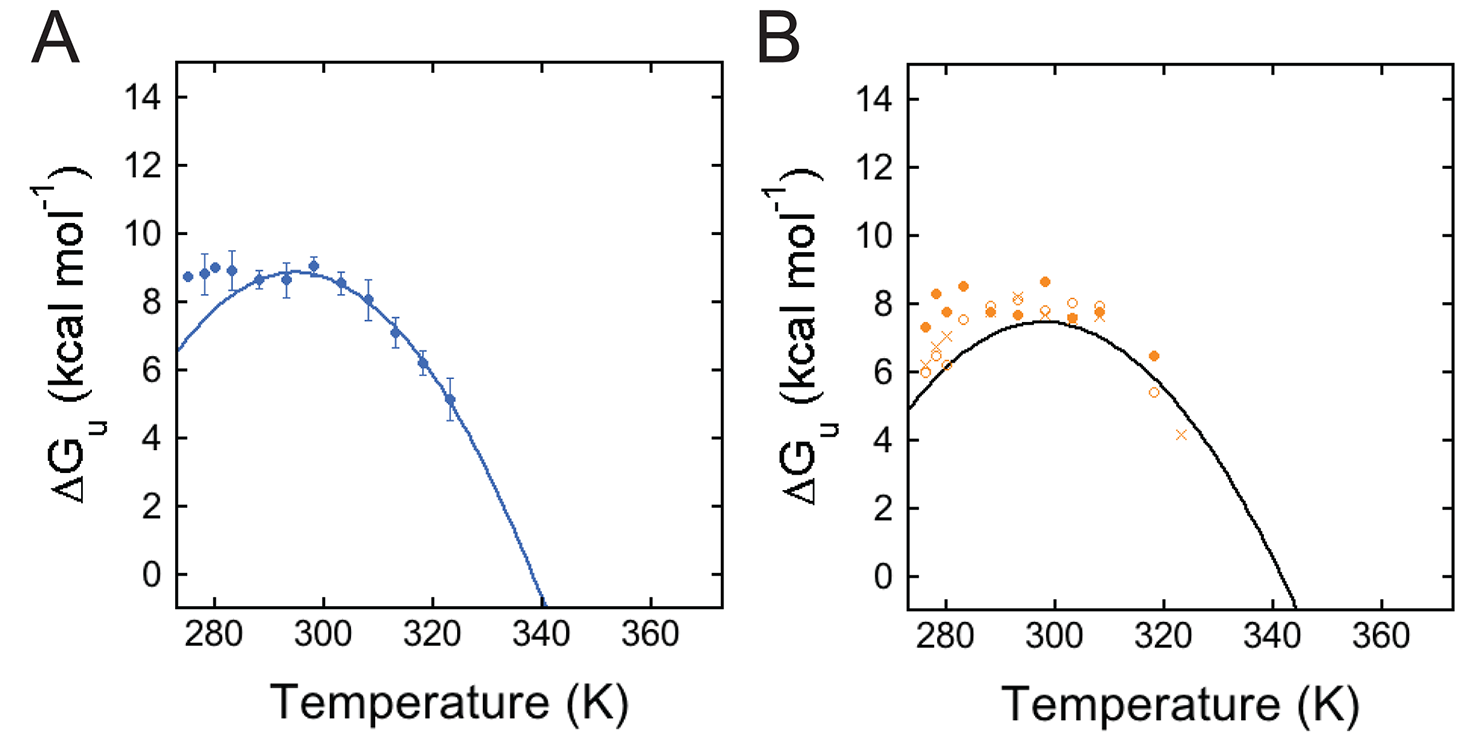

Supplement: Figure S5 — Deviation from two-state behavior at low temperatures. (A) ΔG values of ecRNH unexpectedly plateau below 15°C. Data at 5°C and 10°C reflect the averages of 12 and seven independent experiments, respectively. The displayed fit does not include data below 15°C. (B) Stability curve fit from the cysteine-free variant ecRNH C13A/C63A/C133A (black curve) superimposed with data from single cysteine variants (ecRNH C13A/C133A, orange closed circles; ecRNH C13A/C63A, orange open circles; ecRNH C133A/C63A, orange Xs). Asymmetry in ecRNH stability data is due to C63. See also Dataset S2. (TIF) [file pbio.1001994.s005.tif]

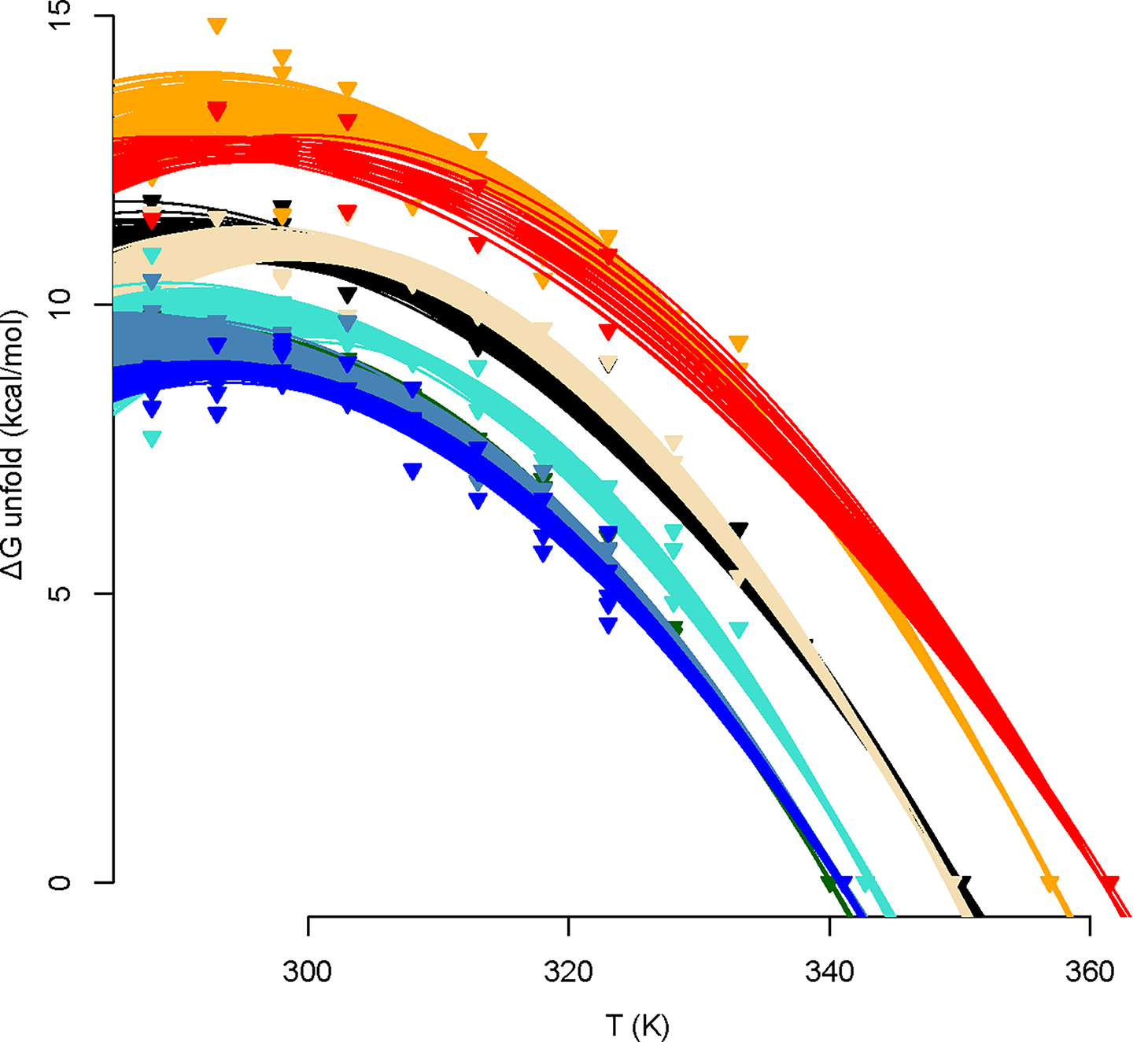

Supplement: Figure S6 — Global fits to bootstrap samples of stability versus temperature curves of different RNH proteins. Points show ΔGunf values measured for each protein as a function of temperature. Lines show a random sample of 100 re-fits of these data generated by bootstrap sampling. Colors denote different proteins: Anc1 (black), Anc2 (wheat), Anc3 (orange), ttRNH (red), AncA (light blue), AncC (dark green), AncD (slate), and ecRNH (blue). Arrows show the maximum likelihood trajectory through this space, starting from Anc1 and going to ttRNH (red path) or starting from Anc1 and going to ecRNH (blue). (TIF) [file pbio.1001994.s006.tif]

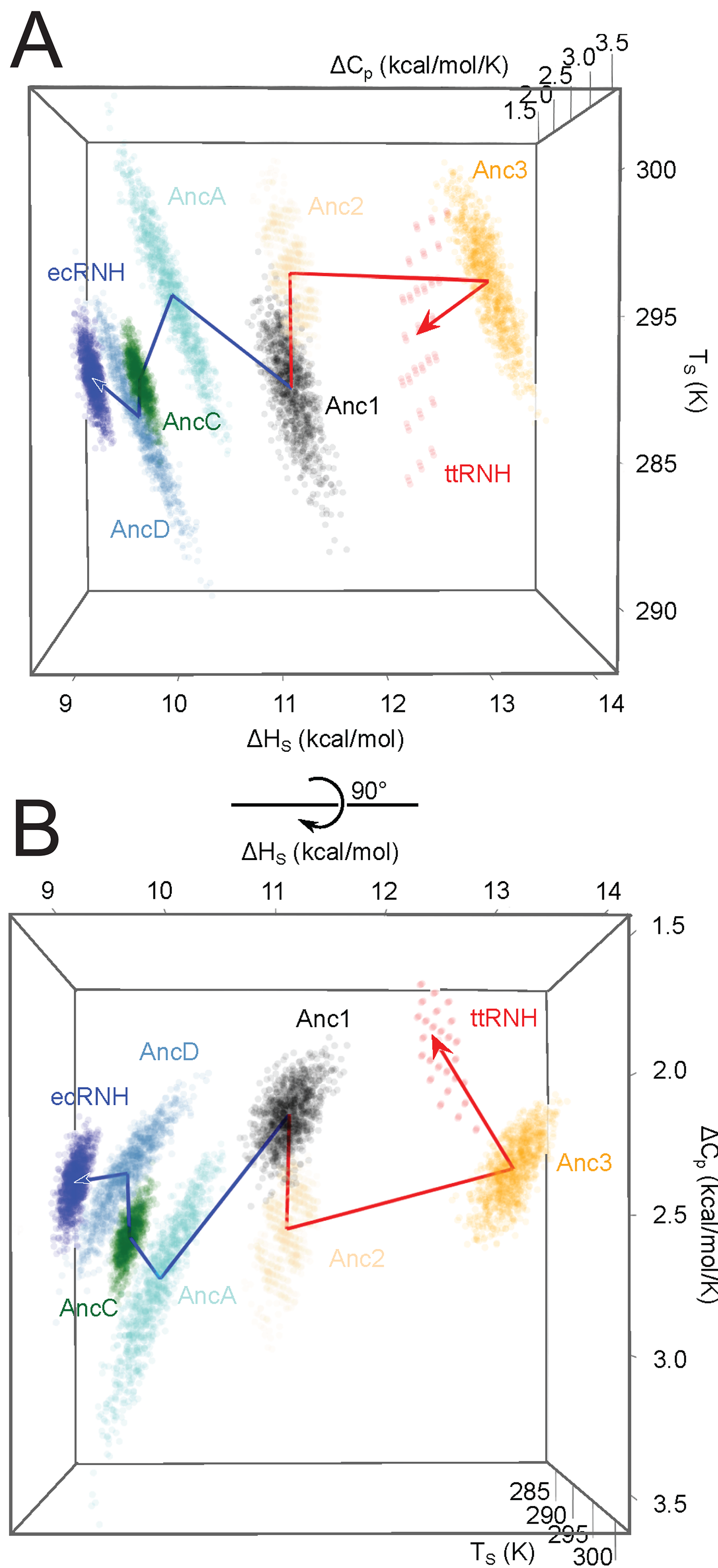

Supplement: Figure S7 — No smooth pathway exists through possible ancestral parameter space. Plots projection of 3D plot of ΔCp versus Ts versus ΔHs for each protein. Points show fit parameters extracted from bootstrap replicates. Colors denote different proteins: Anc1 (black), Anc2 (wheat), Anc3 (orange), ttRNH (red), AncA (light blue), AncC (dark green), AncD (slate), and ecRNH (blue). Arrows show the maximum likelihood trajectory through this space, starting from Anc1 and going to ttRNH (red path) or starting from Anc1 and going to ecRNH (blue). (TIF) [file pbio.1001994.s007.tif]

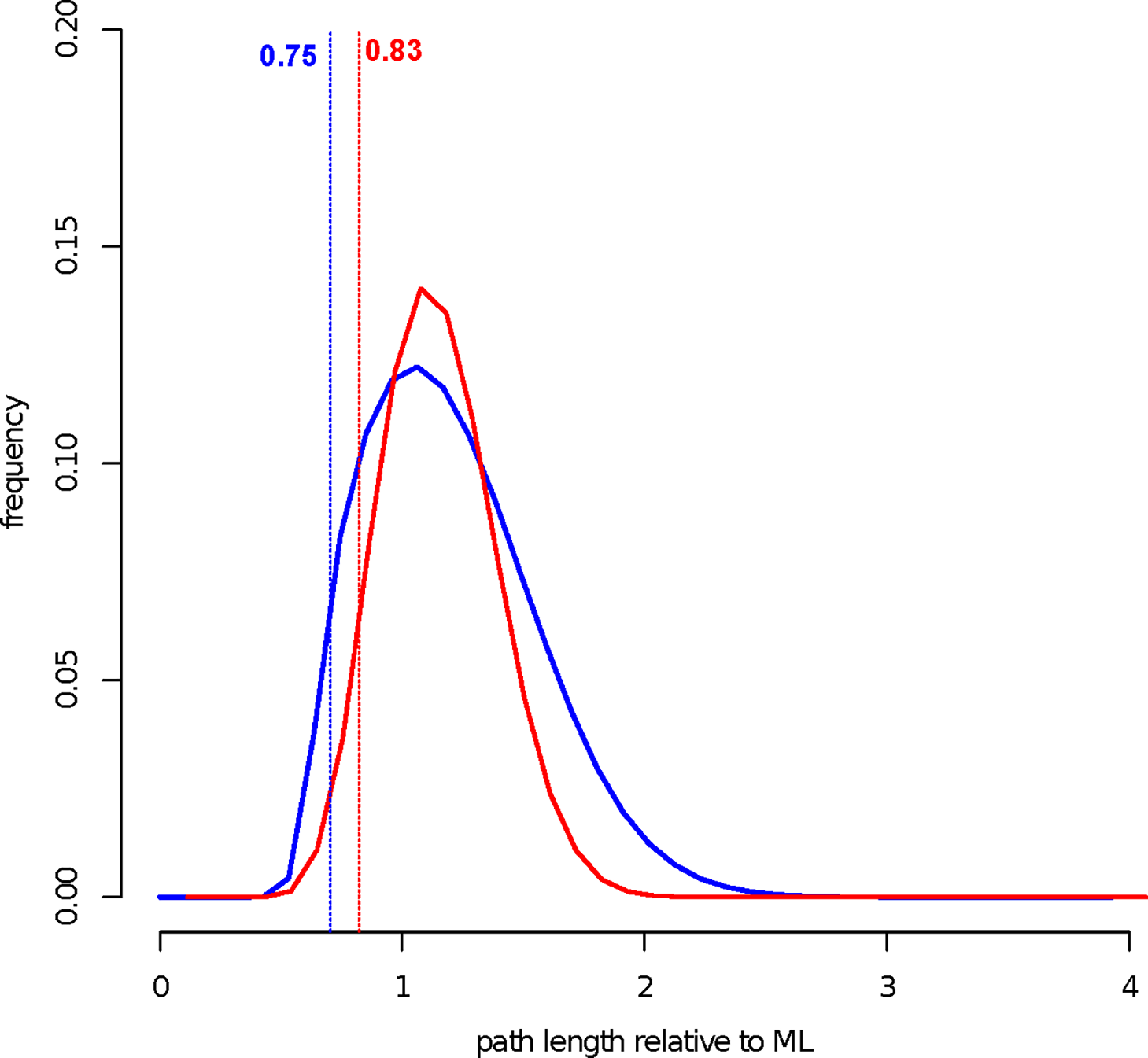

Supplement: Figure S8 — Distribution of all possible path lengths reveals that 95% of possible paths are nearly as long as the ML path. Histograms of possible path lengths weighted by path likelihoods, extracted from explicit enumeration of pathways through the parameter space. Path lengths are normalized to the maximum likelihood path. Red and blue curves denote the thermophilic and mesophilic lineages, respectively. Dashed lines indicate 95% cutoff. (TIF) [file pbio.1001994.s008.tif]
