# Supplementary material for: Thermodynamic System Drift in Protein Evolution
Source: PLoS Biol. 2014 Nov 11;12(11):e1001994. doi: 10.1371/journal.pbio.1001994 (PMC4227636; doi:10.1371/journal.pbio.1001994)
Supplement: Table S1 — Growth temperatures, Tm s and ΔG s at Tenv for extant and ancestral RNH proteins. *Extracted from stability curve fits for two-state proteins. †Errors reported are standard deviations from replicate experiments. ‡Taken from reference [20]. §Taken from reference [21]. ¶Not determined. (DOCX) [file pbio.1001994.s009.docx]

**Table S1.** Growth temperatures, *T_m_*s and *ΔG*s at *T_env_* for extant and ancestral RNH proteins

|  | ***T_m_***^†^ **(°C)** | ***T_env_* (°C)** | ***ΔG***^*^ ***at T_env_* (kcal mol^-1^)** |
| --- | --- | --- | --- |
| ***Thermus thermophilus*** | 88.5 | 68 | 6.3 |
| ***Chlorobium tepidum*** | 66.5^§^ | 48^§^ | 4.6^§^ |
| ***Escherichia coli*** | 68.0 ± 0.5 | 37 | 8.1 |
| ***Klebsiella pneumoniae*** | 68.4 ± 0.3 | 37 | 7.8 |
| ***Citrobacter sp. 30_2*** | 62.4 ± 0.4 | 37 | 6.2 |
| ***Cronobacter turicensis*** | 63.8 | 37 | ND^¶^ |
| ***Salmonella enterica*** | 59.1 | 37 | ND^¶^ |
| ***Enterobacter sp. 638*** | 63.1 ± 0.1 | 30 | ND^¶^ |
| ***Shewanella oneida*** | 53.2 ± 0.5^‡^ | 30^‡^ | 4.7^‡^ |
| ***C. Hamiltonella defensa*** | 51.7 ± 1.1 | 27 | ND^¶^ |
|  | ***T_m_*^†^ (°C)** | **Reversibility** |  |
| **ttRNH** | 88.5 | reversible |  |
| **Anc3** | 83.3 ± 0.8 | reversible |  |
| **Anc2** | 76.5 ± 0.2 | reversible |  |
| **Anc1** | 76.7 ± 0.3 | reversible |  |
| **AncA** | 69.7 ± 0.2 | reversible |  |
| **AncB** | 67.8 ± 0.1 | irreversible |  |
| **AncC** | 67.1 | irreversible |  |
| **AncD** | 67.7 ± 0.3 | irreversible |  |
| **ecRNH** | 68.0 ± 0.5 | irreversible |  |
|  | ***T_m_*^†^ (°C)** | **Reversibility** |  |
| **Anc1, AltI** | 78.3 ± 0.4 | reversible |  |
| **Anc1, AltII** | 79.8 ± 0.2 | reversible |  |
| **Anc1, AltIII** | 80.0 ± 0.4 | reversible |  |
| **Anc1, AltIV** | 78.4 ± 0.7 | reversible |  |
| **Anc1, AltV** | 77.6 ± 0.4 | reversible |  |
| **Anc1, AltVI** | 75.6 ± 0.8 | reversible |  |
| **Anc1, AltVII** | 77.0 ± 0.5 | reversible |  |
| **Anc1, AltVIII** | 76.6 ± 0.5 | reversible |  |
| **Anc1, AltIX** | 80.5 ± 0.5 | reversible |  |
| **Anc1, AltX** | 79.9 ± 0.2 | reversible |  |

^*^Extracted from stability curve fits for two-state proteins.

^†^Errors reported are standard deviations from replicate experiments.

^‡^Taken from reference [20].

^§^Taken from reference [21].

^¶^Not determined.
