# Supplementary material for: Thermodynamic System Drift in Protein Evolution
Source: PLoS Biol. 2014 Nov 11;12(11):e1001994. doi: 10.1371/journal.pbio.1001994 (PMC4227636; doi:10.1371/journal.pbio.1001994)
Supplement: Table S2 — Statistical support for resurrected ancestors. (DOCX) [file pbio.1001994.s010.docx]

**Table S2**. Statistical support for resurrected ancestors

| **Ancestor** | **Tree Support** | | **Sequence Support** | |
| --- | --- | --- | --- | --- |
|  | **e^(LR/2)** | **likelihood ratio** | **ln(likelihood)** | **mean PP** |
| Anc1 | 6.84 | 30.5 | -14.5 | 0.925 |
| Anc2 | 9.99 | 148 | -19.8 | 0.858 |
| Anc3 | 74.79 | 1.74 x 10^16^ | -11.7 | 0.912 |
| AncA | 22.21 | 6.62 x 10^4^ | -17.2 | 0.947 |
| AncB | 28.11 | 1.30 x 10^6^ | -11.6 | 0.957 |
| AncC | 31.40 | 8.45 x 10^6^ | -9.06 | 0.954 |
| AncD | 21.60 | 4.90 x 10^4^ | -4.63 | 0.977 |
