# Supplementary material for: Thermodynamic System Drift in Protein Evolution
Source: PLoS Biol. 2014 Nov 11;12(11):e1001994. doi: 10.1371/journal.pbio.1001994 (PMC4227636; doi:10.1371/journal.pbio.1001994)
Supplement: Table S3 — Data collection and refinement statistics for AncC. *Values in parentheses are for highest-resolution shell. (DOCX) [file pbio.1001994.s011.docx]

**Table S3.** Data collection and refinement statistics for AncC

AncC

| Data collection |  |
| --- | --- |
| Space group | *P*6_1_ |
| Cell dimensions |  |
| *a*, *b*, *c* (Å) | 82.38, 82.38 44.65 |
|    γ (°) | 90, 90, 120 |
| Resolution (Å) | 50-1.36 (1.41-1.36)^*^ |
| *R*_sym_ | 7.9 (85.8)^*^ |
| *I* / *I* | 26.1 (2.7)^*^ |
| Completeness (%) | 99.1 (99.7)^*^ |
| Redundancy | 7.6 (7.2)^*^ |
| Refinement |  |
| Resolution (Å) | 50-1.36 |
| No. reflections | 37219 |
| *R*_work_ / *R*_free_ | 13.4 / 16.6 |
| No. atoms |  |
| Protein | 2642 |
| Water | 237 |
| Sulfate | 2 |
| *B*-factors |  |
| Protein | 18.7 |
| Water | 28.6 |
| Sulfate | 25.5 |
| R.m.s. deviations |  |
| Bond lengths (Å) | 0.012 |
| Bond angles (°)  Ramachandran plot (%) | 1.3 |
| Favored  Allowed  Disallowed | 98.7  1.3  0 |

^*^Values in parentheses are for highest-resolution shell.
