# Supplementary material for: Thermodynamic System Drift in Protein Evolution
Source: PLoS Biol. 2014 Nov 11;12(11):e1001994. doi: 10.1371/journal.pbio.1001994 (PMC4227636; doi:10.1371/journal.pbio.1001994)
Supplement: Table S4 — ΔG s and m -values at 25°C. *Errors reported are standard deviations from replicate experiments. (DOCX) [file pbio.1001994.s012.docx]

**Table S4.** *ΔG*s and *m-*values at 25 °C

|  | ***ΔG***^*^  **(kcal mol^-1^)** | ***m-*value**^*^  **(kcal mol^-1^ M^-1^)** |
| --- | --- | --- |
| **ttRNH** | 12.8 | 3.93 |
| **Anc3** | 13.3 ± 1.2 | 3.84 ± 0.12 |
| **Anc2** | 10.6 ± 0.3 | 3.80 ± 0.10 |
| **Anc1** | 10.9 ± 1.1 | 4.23 ± 0.09 |
| **AncA** | 9.9 ± 0.2 | 4.69 ± 0.08 |
| **AncC** | 9.2 ± 0.3 | 4.99 ± 0.15 |
| **AncD** | 9.4 ± 0.1 | 5.25 ± 0.07 |
| **ecRNH** | 9.0 ± 0.3 | 4.99 ± 0.17 |

*Errors reported are standard deviations from replicate experiments
