# Supplementary material for: Thermodynamic System Drift in Protein Evolution
Source: PLoS Biol. 2014 Nov 11;12(11):e1001994. doi: 10.1371/journal.pbio.1001994 (PMC4227636; doi:10.1371/journal.pbio.1001994)
Supplement: Table S5 — Thermodynamic parameters from stability curve fits. * Errors from fit. † Extracted from thermal melt fit. ‡ Extracted from stability curve fit. (DOCX) [file pbio.1001994.s013.docx]

**Table S5.** Thermodynamic parameters from stability curve fits

|  | ***ΔC_p_***  **(kcal mol^-1^ K^-1^)** | ***T_m, fit_*** / ***T_m, meas_* (K)** | ***ΔH_m_***  **(kcal mol^-1^)** | ***T_s_* (K)** | ***ΔH_s_***  **(kcal mol^-1^)** |
| --- | --- | --- | --- | --- | --- |
| **ttRNH** | 1.91 ± 0.30^*^ | 361^‡^ / 361^†^ | 136 ± 10^*^ | 297 ± 5* | 12.5 ± 0.3^*^ |
| **Anc3** | 2.29 ± 0.30^*^ | 357^‡^ / 356^†^ | 152 ± 9^*^ | 297 ± 3* | 13.3 ± 0.3^*^ |
| **Anc2** | 2.53 ± 0.20^*^ | 350^‡^ / 350^†^ | 144 ± 5^*^ | 297 ± 2* | 11.1 ± 0.2^*^ |
| **Anc1** | 2.28 ± 0.19^*^ | 349^‡^ / 350^†^ | 136 ± 5^*^ | 294 ± 2* | 11.1 ± 0.2^*^ |
| **AncA** | 2.67 ± 0.24^*^ | 344^‡^ / 343^†^ | 137 ± 6^*^ | 296 ± 2* | 9.8 ± 0.1^*^ |
| **AncC** | 2.14 ± 0.15^*^ | 342^‡^ / 340^†^ | 121 ± 4^*^ | 290 ± 1* | 9.4 ± 0.1^*^ |
| **AncD** | 2.14 ± 0.40^*^ | 342^‡^ / 341^†^ | 121 ± 10^*^ | 290 ± 3* | 9.4 ± 0.2^*^ |
| **ecRNH** | 2.89 ± 0.31^*^ | 338^‡^ / 341^†^ | 135 ± 7 ^*^ | 295 ± 1* | 8.8 ± 0.1^*^ |

* Errors from fit.

† Extracted from thermal melt fit.

‡ Extracted from stability curve fit.
