# Supplementary material for: Thermodynamic System Drift in Protein Evolution
Source: PLoS Biol. 2014 Nov 11;12(11):e1001994. doi: 10.1371/journal.pbio.1001994 (PMC4227636; doi:10.1371/journal.pbio.1001994)
Supplement: Table S6 — Individual proteins populate discrete regions of parameter space. (DOCX) [file pbio.1001994.s014.docx]

**Table S6.** Individual proteins populate discrete regions of parameter space. Table shows fraction of points that overlap between each protein. Non-zero values are bold.

|  | **ttRNH** | **Anc3** | **Anc2** | **Anc1** | **AncA** | **AncC** | **AncD** | **ecRNH** |
| --- | --- | --- | --- | --- | --- | --- | --- | --- |
| **ttRNH** | 1.000 |  |  |  |  |  |  |  |
| **Anc3** | 0.000 | 1.000 |  |  |  |  |  |  |
| **Anc2** | 0.000 | 0.000 | 1.000 |  |  |  |  |  |
| **Anc1** | 0.000 | 0.000 | **0.003** | 1.000 |  |  |  |  |
| **AncA** | 0.000 | 0.000 | 0.000 | 0.000 | 1.000 |  |  |  |
| **AncC** | 0.000 | 0.000 | 0.000 | 0.000 | 0.000 | 1.000 |  |  |
| **AncD** | 0.000 | 0.000 | 0.000 | 0.000 | 0.000 | **0.004** | 1.000 |  |
| **ecRNH** | 0.000 | 0.000 | 0.000 | 0.000 | 0.000 | 0.000 | **0.005** | 1.000 |
