# Supplementary material for: Thermodynamic System Drift in Protein Evolution
Source: PLoS Biol. 2014 Nov 11;12(11):e1001994. doi: 10.1371/journal.pbio.1001994 (PMC4227636; doi:10.1371/journal.pbio.1001994)
Supplement: Dataset S1 — RNH sequence alignment. (DOCX) [file pbio.1001994.s015.docx]

**Dataset S1.** RNH sequence alignment

>Chloroflexi | Herpetosiphon au | YP_001547468

-----------------------------------------KVVLFSDGGSDG-NP-GP----GGYGVVLR---S-G-S--------EMR-----ELTGGFAR-TTNNRMELMGVITGLQAL-------SQP------SKVVVYSDSAYVINGMHKGWAERWSKNGWRTTTG----PVKNPDLWQQLLELAQG--H-TI-EWVQVPGHAGVKDNERCDRLAVQAA-----HQPNL-----PIDQGYRD-----------------------

>Firmicutes | Halothermothrix | YP_002508646

----------------------------------REMTKMEPIKVYTDGACSG-NP-GP----GGYAAVIL---NQG----------QER-----VVAGYEDE-TTNNRMELRAVIEALKEI-------KEG------REVHVYSDSSYIINGMK-SWIDDWKKRGWKTSSNK---PVSNKDLWLKLDNLSSK--F-NI-KFKKVKGHSGDEYNEKADSLARKQI-----EENS------PE-----------------------------

>Deinococcus-Thermus | Deinococcus radi | NP_294623

------MTRPGRPSARKKPDTSRDLLPIRAGIQPEVPVGGQVVELYSDGACDT-TK-GH----GGWATILR---Y-G-E--------REL-----VLSGNEEN-TTNNRMELRGLLEGLRTL-------RRP------CQVKVITDSQYLRKAFTDGWILNWQRNGWKTASKE---PVKNQDLWEELIELAKV--H-AL-TFLWVKGHAGHGENERVDELAVLER-----KKLRK------------------------------------

>Deinococcus-Thermus | Deinococcus geot | YP_001527665

-----------------------------------MSPPLTAVRLVTDGACSG-NP-GP----GGWACILS---S-G-A--------STR-----ELSGGEAQ-TTNNRMELTALLEGLRAL-------KRP------CQVHVVSDSRYIIDAFEQGWLAGWQAKGWK--------KVKNPDLWQAIAEAARG--H-TL-TFEWVQGHAGHPENERADQLAVQAR-----EQAARQPPAPPSGPAGGLF----------------------

>Firmicutes | Clostridium botu | YP_001780057

---------------------------------------MKKVIIYTDGACRG-N--GQENTIGAYGIVLM---Y-G-E--------HKK-----EIKKAFRD-TTNNIMELSAVVEALSLL-------KKP------CSIELYSDSAYVINAINQKWLDNWKKNNWKTASKS---PVKNKELWEKLDELLKK--H-SV-KFIKVKGHSDNEYNNRCDKLANEAM-----DEFNV------------------------------------

>Bacteroidetes | Salinibacter rub | YP_445310

----------------------------------CFFGFMNEVTIYTDGACSG-NP-GP----GGWAAILLPDDD-S-D--------ATD-----PLTGGEPH-TTNNRMELTAALEALRAL-------DDR------SRVALHTDSEYLSKAFNEGWLDSWQDNNWQTSSND---DVKNQDLWKALLEEADR--H-EV-DWVWVKGHADDELNIMADELAVAAM-----EQYK-------------------------------------

>Bacteroidetes | Rhodothermus mar | YP_003291257

-----------------------------------MSTPRKHVVIYTDGACSG-NP-GP----GGWAAILR---Y-N-Q--------HEK-----VLTGAAPH-TTNNRMELTAVIEALRAL-------KEP------CRVDVYTDSNYIVRAFQEGWVDRWQRNGWRTASKK---PVENQDLWRALLELTRR--H-DV-RFLKVKGHADDALNNRVDRLAVEAM-----RRGQ------TKAAGSAVND---------------------

>Tenericutes | Candidatus Desul | YP_001717962

---------------------------------MGEQPTMSEVVIYTDGACSG-NP-GP----GGWAAVIL---D-G-V--------ARR-----ELTGSDPK-TTNQRMELLAAIRSLQAL-------GEE-----PRRVTLYSDSAYLVNCFRDRWYERWEQNGWVNAKKQ---PVQNRDLWEELLRLARR--H-RV-TFRKIKGHGSNELNNRADALARGAL----PPGAR-------------------------------------

>Firmicutes | Syntrophomonas w | YP_754778

---------------------------------------MKEIIIYTDGACSG-NP-GP----GGWGAVLA---Y-G-E--------HQK-----EIAGAEAD-TTNQRMELMAVIEALKAI-------KGS-----GWEIRVYSDSAYFINAIQKGWLENWQRNGWKNSKKE---DVANQDLWKALIPLLRK--N-RV-RVEKVKGHSGDRWNERCDQLARNAI-----KSLG-------------------------------------

>Firmicutes | Natranaerobius t | YP_001917343

--------------------------------MTNTNEDKKRVTIYTDGACSG-NP-GP----GGWGAILL---F-N-E--------HKK-----ELSGSAEN-TTNQRMELYAAVQALKAL-------KYP------CNVELCSDSAYLVNCFQQGWWKKWQRNNWLTKSKK---KVDNQDLWRELIELNDY--H-SI-QWIKVKGHSDDELNNRADQLATEAI-----PDKS-------------------------------------

>Firmicutes | Alicyclobacillus | YP_003183894

-------------------------------------MSDETVILYTDGACSG-NP-GP----GGWAAILQ---W-N-G--------HVK-----ELSGGERE-TTNQRMELKAVIEGLKAL-------KRP------CDVIVHSDSAYVVNCFKQRWYVNWRKNGWINSKGE---PVQNRDLWEQLLEAIDG--H-RV-RFEKVKGHAGVKWNERCDELARSAI-------PR-------------------------------------

>Chlorobi | Chloroherpeton t | YP_001996233

---------------------------------------MKQVVIYTDGACSG-NP-GK----GGWGAVLI---F-G-E--------KRR-----EISGYEAQ-TTNNRMEMMAAIQALEQL-------KEP------CAVDLYSDSSYLVNAFNEGWIDGWLRRGWKTAGKK---PVLNQDLWQRLLGLTSS--H-QV-TFHKVKGHSTDELNNRCDFLATEAI----KTEGA-------------------------------------

>Chlorobi | Pelodictyon phae | YP_002017618

--------------------------------------METKITIYTGRRIAA-QI-RA----LLSGCAVD---V-R-L--------HDS-----SIAGYSPA-TTNNRMELSAAIEALEAL-------KEP------CRVDLYSDSSYLVNAINEGWLKRWTINNWKTSTKK---NVENIDLWKKILKLLTL--H-TI-TFHKVKGHSDNPYNNRCDTLAREAI-----KKKS-------------------------------------

>Chlorobi | Chlorobium chlor | YP_380038

--------------------------------------MKKQVTIYTDGACSG-NP-GP----GGWGALLM---F-G-S--------ITR-----EVSGSSPA-TTNNRMELGAAIEALALL-------KEP------CLVDLYSDSSYLVNAINNGWLQRWQRNSWQTAAKK---SVENIDLWQKLIKLLKV--H-EV-RFHKVKGHSDNAYNNRCDQLAREAI-----KKTS-------------------------------------

>Chlorobi | Chlorobium limic | YP_001942694

--------------------------------------MKKRVTIYTDGACSG-NP-GR----GGWGALMM---Y-G-T--------VNR-----ELSGYEPA-TTNNRMELTAAIEGLDAL-------KEP------CVVDLYSDSAYLVNALNQGWLKRWTTNNWTTSAKK---SVENIDLWKKILKLVTL--H-QV-TFHKVKGHSDNPFNNRCDELARQAI-----KNNS-------------------------------------

>Chlorobi | Chlorobaculum pa | YP_001998194

--------------------------------------MEKTITIYTDGACSG-NP-GK----GGWGALLM---Y-G-N--------TRK-----EISGYDPA-TTNNRMEMMAAIRALEAL-------KEP------CRVELYSDSAYLVNAMNQGWLKRWLKNGWKTASKK---PVENIDLWQEIVKLTTL--H-RV-TFHKVKGHSDNQYNNRCDELARLAI-----KEQS-------------------------------------

>Chlorobi | Chlorobium tepid | NP_662495

--------------------------------------MEKTITIYTDGACSG-NP-GK----GGWGALLM---Y-G-S--------SRK-----EISGYDPA-TTNNRMELMAAIKGLEAL-------KEP------CRVQLYSDSAYLVNAMNEGWLKRWVKNGWKTAAKK---PVENIDLWQEILKLTTL--H-RV-TFHKVKGHSDNPYNSRCDELARLAI-----KENS-------------------------------------

>Chlorobi | Chlorobium phaeo | YP_001130910

--------------------------------------MEKKVTIYTDGACSG-NP-GP----GGWGAMLM---Y-G-K--------TVR-----EISGGAPA-TTNNRMELSAAIEALQAL-------KEP------CTVDLYSDSSYLVNAINEGWLKRWTANRWKTAAKK---TVENIDLWQKILELTDR--H-RV-RFHKVKGHSDNPYNNRCDELARLAV-----RKKP-------------------------------------

>Chlorobi | Chlorobium luteo | YP_375504

-------------------------------MVEPFLSMQKKITIYTDGACSG-NP-GK----GGWGAMLM---Y-G-D--------AVR-----ELSGYSPA-TTNNRMELTAAIEALRAL-------KEP------CSVALYSDSSYVVNAFREGWLDRWTRNNWKTAAKK---NVENTDLWKQILELTAR--H-TV-TFHKVKGHSDNPYNNRCDELARQAI-----QKKP-------------------------------------

>Chlorobi | Chlorobium ferro | ZP_01385334

--------------------------------------MQKKLIIYTDGACSG-NP-GP----GGWGALLM---Y-G-P--------STR-----ELSGYSPA-TTNNRMELTAAIEALEAL-------KEP------CRVDLYSDSSYLVNAINEGWLKRWVVNNWKTAAKK---NVENPDLWQKILKLIRL--H-EV-TFHKVKGHSDNPYNNRCDVLAREAI-----KKHP-------------------------------------

>Chlorobi | Chlorobium phaeo | YP_911061

--------------------------------------MQKKIIVYTDGACSG-NP-GK----GGWGALLM---Y-G-A--------STR-----EISGYSPA-TTNNRMELSAAIEALETL-------KEP------CIVHLYSDSSYLVNAINEGWLKRWTANNWKTAAKK---SVENIDLWQKILTLIKL--H-DV-TFHKVKGHSDNPYNNRCDELARQAI-----KNNR-------------------------------------

>Chlorobi | Prosthecochloris | YP_002015246

--------------------------------------MRKKIIIYTDGACSG-NP-GK----GGWGALLM---F-G-E--------LNR-----EISGYSPA-TTNNRMELMAAIQALEAL-------KEP------CDVDLYSDSSYLVNAIKLGWLKKWSSGGWTTASRK---PVENQDLWKKILQLIKL--H-NV-TFHKVKGHSDNEYNNRCDYLARQAI-----KNNR-------------------------------------

>Chlorobi | Chlorobium phaeo | YP_001959040

--------------------------------------MQKKVTIYTDGACSG-NP-GK----GGWGALLM---F-G-S--------VKR-----ELSGYSPA-TTNNRMELMAAIQALEAL-------KEP------CEVALYSDSSYLVNAINKGWLKRWTSNNWKTAAKK---PVENIDLWKMILELIRL--H-SV-TFHKVKGHSDNEFNNRCDYLATQAI-----KNNR-------------------------------------

>Firmicutes | Clostridium ther | YP_001037101

---------------------------------------MKKVSIYTDGACSG-NP-GD----GGWGAILI---Y-G-N--------HEK-----EVSGFEKD-TTNNRMELVAAINALKML-------KEP------CEVDLYSDSAYLVNGFLQNWVEKWKKNGWKTSNKE---EVKNMELWQELDRLSNI--H-KI-RWIKVKGHSDNEYNNRCDKLATDEI-----KKNS------KK-----------------------------

>Firmicutes | Clostridium papy | ZP_05494226

---------------------------------------MKQVEIYTDGACSG-NP-GA----GGWGAVLM---Y-G-E--------HKV-----EISGFEKS-TTNNKMELTAAFEALKRL-------KEP------CKVNLYSDSAYLVNAFLQGWLDKWIKNGWKRNKNE---EVKNIELWKELVRLADI--H-EI-KWIKVKGHADNVYNNRCDKLATDEI-----KKNC-------------------------------------

>Firmicutes | Clostridium cell | YP_002506085

---------------------------------------MKQIEIYTDGACSG-NP-GA----GGWGAVLM---Y-G-E--------HKI-----EISGFEKS-TTNNKMELTAAYEALKRL-------KEP------CRVNLYSDSAYLVNAFLQGWLDKWIKNGWKRNKNE---EVKNVDLWKELVKLADI--H-EI-KWIKVKGHADNEYNNRCDKLATDEI-----KKNS-------------------------------------

>Firmicutes | Brevibacillus br | YP_002770554

-------------------------------------MTMREVEIYTDGACSG-NP-GP----GGWGAVLM---Y-G-Q--------HIK-----EMSGAEPH-TTNNRMELMAAIKALSTL-------KEP------CKVTLSSDSAYLVNCFKQGWYKGWLKNGWKNSKGQ---QVENQDLWKELLQLMDT--H-KV-EYVKVKGHADNKWNNRCDELATGAI-----KQL--------------------------------------

>Firmicutes | Paenibacillus sp | ZP_04854933

---------------------------------------MKEVTIYTDGACSG-NP-GP----GGWGAVLM---F-N-G--------HRK-----DLSGGEKM-TTNNRMEIQAVISALSQL-------KEP------CQVKVYSDSAYVVNCFQQNWIRGWLKNGWKNSKNQ---PVENRDLWEELWRLMGI--H-KV-EYIKVKGHSDNELNNYCDQLAREAI-----KRLSS------------------------------------

>Firmicutes | Paenibacillus sp | YP_003009571

---------------------------------------MKEVTIYTDGACSG-NP-GP----GGWGAVLF---Y-G-V--------HRK-----ELSGGEKH-STNNRMEIQAVIEALNLL-------KEP------CKAKIYSDSAYVVNCFQKGWIHGWLRNGWKNSKKE---PVENQDLWKTLWDLMKR--H-QV-EYIKVKGHSDNEWNNRCDELAREAI-----KRL--------------------------------------

>Firmicutes | Dethiobacter alk | ZP_03729095

---------------------------------------MKDVIIYTDGACSG-NP-GP----GGWGAVLR---Y-G-S--------HEK-----EISGGDEK-TTNQRMELQAAISALELL-------KEP------CKVKLHSDSAYLVNAFKQRWFDKWQKNGWVNSKKE---PVVNRDLWERLLELDRK--H-DI-EWVKVKGHADDELNNRCDQLARDAV-------PR-------------------------------------

>Proteobacteria beta | Moorella thermoa | YP_429492

---------------------------------------MKEVTIYTDGACSG-NP-GP----GGWGAVLI---Y-G-D--------KRK-----ELSGAEPS-TTNQRMEITAAIAALRVL-------KEP------CRVHLYSDSAYLVNAFRQGWLARWERNGWLTVKKQ---PVENQDLWRELLQVASR--H-QV-EWLKVKGHSDNPENNRCDELARAAI-----AALR------RQEIPSS------------------------

>Firmicutes | Symbiobacterium | YP_076749

---------------------------------------MREVIIYTDGACSG-NP-GP----GGWGAVLL---Y-G-S--------HRK-----ELSGFHPH-TTNNRMEIQAAIEALRAL-------KYP------CKVKLYSDSAYLVNAFRQNWLRTWQRNGWVNSRKQ---PVENQDLWQELLEAARP--H-QV-EWLKVQGHADVAENNRCDELARAAI-----AAGT------QG-----------------------------

>Firmicutes | Anaerocellum the | YP_002573213

---------------------------------------MKEVTIYTDGACSG-NP-GP----GGWCAILI---Y-K-G--------IKK-----VLKGFERY-TTNNRMELKAVVEALKAL-------KEP------CKVVIYSDSAYIVNAVNQNWIEKWQKNGWKTSEKE---EVKNIDLWNELVELMKI--H-KV-TFEKVKGHADNELNNLCDRIARSMI-----KGEQ-------------------------------------

>Firmicutes | Caldicellulosiru | YP_001180679

---------------------------------------MKEVVIYTDGACSK-NP-GP----GGWCAILI---Y-K-G--------IKK-----VLKGFEEN-TTNNRMELKAIIEGLKAL-------KEP------CKVTVYTDSAYIVNAINQNWIGKWQKNNWKTSEKE---EVKNIDLWQELLEFLKV--H-NV-KFEKVKGHSTDTLNNMCDEIARSMI-----KEMR-------------------------------------

>Firmicutes | Thermoanaerobact | ZP_05334886

------------------------------------MANIPEIDIYTDGACSG-NP-GP----GGWGAVLI---Y-N-G--------IKK-----EISGYEEN-TTNNRMELTAVIKALSLL-------KRS------CKINIYSDSSYLINAFNQKWIENWQKRGWLKSDKT---PVENKDLWLKLLDLSSC--H-DI-KWIKVKGHSDNEYNNRCDKLATDEI-----RKHSI------------------------------------

>Firmicutes | Thermoanaerobact | NP_622980

-----------------------------------MKNNNEIVEIYTDGACSG-NP-GP----GGWAAVLI---Y-K-G--------IKK-----EISGFEEN-TTNNRMELKAAIEGLKAL-------KRP------CKVNLYSDSSYLINAFNEGWIEKWQKNNWLKSDKT---PVENQDLWKELLEVSKP--H-QI-NWIKVKGHSDNEYNNLCDRLATEQI-----KKHI------KENP---------------------------

>Firmicutes | Thermoanaerobact | ZP_05379402

-----------------------------------MENNIDIVEIYTDGACSG-NP-GP----GGWAAVLL---Y-K-E--------ARK-----EISGFEEN-TTNNRMELKAVIEALKAL-------KRP------CKVNLYSDSSYVINAFKEGWLEKWQKNNWLKSDKT---PVENQELWKELLEVSKR--H-QI-NWIKVKGHADDEFNNLCDRLATEQI-----KRNT------KKL----------------------------

>Firmicutes | Thermoanaerobact | YP_001665169

-----------------------------------MSNNIDVVEIYTDGACSG-NP-GP----GGWAAVLL---Y-K-G--------TKK-----EISGFEEN-TTNNRMELKAVIEGLKAL-------KRP------CKVNLYSDSSYVINAFKEGWLEKWQKNNWLKSDKT---PVENQDLWKELLEISKN--H-QV-NWIKVKGHADNEYNNLCDRLATEQI-----KRNT------RQNPKE-------------------------

>Chloroflexi | Roseiflexus cast | YP_001431304

------------------------------------LLNSGKVVMFTDGCFDS-ES-GS----GGYGVILK---H-R-D--------RTK-----EISGGFRE-TTNNRMEIRACIEGLRAL-------KRP------SEVVIFSDSKYVVDSMSKGWVQRWKDQGWMRNEKD---QAENSDLWEQLLELCNQ--H-RV-EFRWVKGHNHTKENERCDQLASEAA-----KRSD------LPIDRRSP-----------------------

>Thermobaculum | Thermobaculum te | YP_003323183

---------------------------------------MKKVIIHTDGGCEP-NP-GP----GGWAAVIR---Y-N-S--------EVQ-----EISGGEEN-TTNNRMEMTAVIKALEAL-------HEP------HEVELYTDSEYLCKGMM-EWLPMWKAKGRLQKG-----SVKNADLWQRIDELMSR--H-LV-KCYWVKGHAGNTDNERCDKLAYEEI-----KKIYKQKGQTPPPPIQMRLIS--------------------

>Proteobacteria epsilon | Campylobacter ho | YP_001407135

---------------------------------------MKSVKLFSDGSCLG-NP-GI----GGWAYILE---F-N-G--------HEK-----CECGGEML-TTNNKMELRAAIEGLKAL-------KEP------CEVKIFTDSSYVTNSIN-GWLEKWVAKNFK--------GKQNVELWREFLRVSAM--H-KI-SAFWVKGHAGHPQNERCDEMARNFA-----QNLKGA-----------------------------------

>Proteobacteria epsilon | Campylobacter gr | ZP_05625344

---------------------------------------MKSVKLFSDGSCLG-NP-GA----GGWAYILQ---Y-G-D--------AIK-----KASGAEAM-TTNNQMELTAAIMGLSAL-------KQP------CRVELFTDSEYVVKAIS-SWLAKWVATDFK--------GKKNADLWRRYLAAAAP--H-EI-KASWVKGHAGHPQNEECDAMARAAA-----EAIKG------------------------------------

>Proteobacteria epsilon | Campylobacter re | ZP_03611429

---------------------------------------MKTVCLFSDGSCLD-NP-GP----GGWAYILE---Y-G-E--------HKK-----TASGGEAH-TTNNQMELRAAIEGLKAL-------KQP------CRVKLYTDSSYVANAVN-AWLEGWVKKNFK--------NVKNVPLWQEYLAASEP--H-EV-EAIWVKGHAGHPQNELCDEMAREQA-----VKIK------NSLKGE-------------------------

>Proteobacteria epsilon | Campylobacter cu | YP_001409227

---------------------------------------MKTVTLFSDGSCLN-NP-GA----GGWAYILE---F-N-G--------AVK-----KDSGGAAM-TTNNQMELTAVIEGLKAL-------KEP------CEVRLFTDSSYVANAVN-SWLDGWVKKNFIGSDKK---PVKNIELWQEYLRVSRP--H-KV-TASWIKAHNGHPQNEECDTMAREKA-----TKFQ------NEADI--------------------------

>Aquificae | Persephonella ma | YP_002730213

---------------------------------------MKKVEIFTDGSSLG-NP-GA----GGWCAILR---Y-N-K--------HEK-----MIKGGKEN-TTNNEMEIKAVLEALKIL-------KEP------CEIDLYSDSEYVVKAMK-EWIHNWAKNNWKTSKKK---DVAHKDMWQEIYRLMQI--H-RI-NPIWVKAHAGHRENEICDRIAKKEA-----EKFRR------------------------------------

>Proteobacteria epsilon | Helicobacter cin | ZP_03659319

---------------------------------------MKQVTLYCDGSSLG-NP-GA----GGWCGILC---F-K-D--------KQK-----ILSGGEPY-TTNNRMELLAVIESLKAL-------KEP------CVVDLYSDSKYVCDGIN-SWLKNWVAKDFK--------NVKNVDLWQSYLQVSSL--H-SV-TAHWVKGHAGHPQNELCDSLAKQAA-----KDVM------AKDEAVRF-----------------------

>Proteobacteria epsilon | Helicobacter hep | NP_860229

-------------------------------------MIMKQVTLYCDGSALG-NP-GA----GGWCGILS---F-G-D--------KQK-----ILTGGETY-TTNNRMELLAVIESLKAL-------NQP------CIVNVYSDSRYVCNGIN-LWLKSWISKQFK--------NVKNPDLWQLYLQVSSP--H-QV-IAHWVKGHAGVAQNELCDKLAKESA-----QFYL------NKGISDE------------------------

>Proteobacteria epsilon | Campylobacterale | ZP_05070331

---------------------------------------MKKITLFSDGSALG-NP-GP----GGYGVILR---Y-D-D--------KER-----EIVGSEVH-TTNNRMELLGVIEGLRAL-------SEK------CEVDIISDSSYVVKGIN-EWLANWIKKDFK--------KVKNPDLWRDYIEVSQG--H-KI-NAIWVRGHDGHEENERCDKLARDEA-----EKIKASL----------------------------------

>Proteobacteria epsilon | Caminibacter med | ZP_01872048

---------------------------------------MKKIEIYTDGSSLG-NP-GP----GGWCAILR---Y-K-G--------KEK-----IISGGEEY-TTNNRMELKAVIESLKIL-------KEP------CEIELYADSTYVLKGIN-EWLSNWVRKNFK--------NVKNEDLWREFLRYSKP--H-KI-NVNWIKGHSGHIENERCDKIAKDEA-----LRRK------SVSK---------------------------

>Proteobacteria epsilon | Nitratiruptor sp | YP_001355685

---------------------------------------MKKVSLFSDGSSLG-NP-GP----GGYCAILR---Y-K-D--------NEK-----IIKGGEPH-TTNNRMELKAVIEGLKAL-------KEP------CIVTVYSDSNYVVQAIN-SWLSGWIKKDFK--------NVKNPDLWKEFIEVAKP--H-RI-KAVWVKGHSGHEENERCDKIAKEMA-----KEAGIG-----------------------------------

>Planctomycetes | Rhodopirellula b | NP_869340

------------------------------MTDSKTEAAFKPVELYTDGACSG-NP-GP----GGWAFVLR---CPRTL--------KEI-----QRSGGQPH-TTNNQMELMAVIRGLEAL-------KEP------CAVDLYSDSKYVGQGMS-SWMAGWKSRGWKRKDGSKLVPVKNVELWQELDQQMQA--H-RV-TYHHVKGHAGHTENELCDKLAVAAY-----QQYL-------------------------------------

>Proteobacteria alpha | Ehrlichia rumina | YP_196682

----------------------------MSSFICCMKDELNKVVVYTDGACSG-NP-GP----GGWGAVLL---F-D-N--------GEK-----TICGGHPN-TTNNRMELTAVVQALKFL-------DVT------YVIDLYTDSVYVKSGIT-SWIKKWKINGWRTADKL---PVKNLELWLELDKIVKY--H-KI-TWYWVKAHSGNLYNEKADMLARSQI-------VK-------------------------------------

>Proteobacteria alpha | Ehrlichia chaffe | ZP_00544752

-----------------------------------MKDELNKVVIYTDGACSG-NP-GP----GGWAAVLL---F-D-D--------NEK-----TICGNDSD-TTNNRMELTAVIEALKLL-------KVA------YNVDLYTDSVYVKDGIT-LWIRKWKVNGWKTANKM---PVKNLELWLELDSLANF--H-KV-TWYWVRAHVGDLYNQKADMLARSQI-------VR-------------------------------------

>Proteobacteria alpha | Ehrlichia canis | YP_303391

-----------------------------------MKDELNKVVIYTDGACSG-NP-GP----GGWGAILL---F-D-K--------NER-----TICGNNPD-TTNNRMELTAVIEALKFL-------KVA------YNVDLYTDSIYVKDGIT-LWIEKWKINGWRTASKL---PVKNLELWLELDSLASF--H-NV-TWYWVKAHAGNLYNQKADILARSQI-----SK---------------------------------------

>Proteobacteria alpha | Wolbachia endosy | ZP_03335577

-----------------------------MLNLYPVNMDKKKVIIYTDGACSG-NP-GP----GGWAAVVM---Y---E---NKSVFIKK-----RISGGEEN-TTNNKMELKAVINGLKML-------KIS------CKVIVHTDSQYIKQGIT-EWINKWKTNGWKTADKK---PVKNRELWQELDEVALQ--H-DI-NWKWVRAHNGNMYNEEADRLARKES-----KNLKYRDCEVKKSPKNRGNSKFHRLGGVLWQ----------

>Proteobacteria alpha | Wolbachia endosy | YP_001974908

-------------------------------------MKKKEVTIYTDGACSG-NP-GT----GGWAAIIL---F-Q-N--------HRK-----NICGREEN-TTNNKMELTAVINGLKVL-------KFP------CNISLYTDSLYIKYGIT-EWINKWKMNGWKTSNKK---SVKNIELWKELDNAALQ--H-EI-NWNWVKAHNGDKYNEEADILARKAI-----INA--------------------------------------

>Proteobacteria alpha | Anaplasma centra | YP_003328923

-----------------MSLYYVRYWNTIKNDGRMVLMGKSRVAIYTDGACSG-NP-GP----GGWGAVLR---F-GDG--------EER-----RISGGSDD-TTNNRMELTAVIMALAAL-------SGP------CSVCVNTDSTYVKNGIT-EWIRKWKLNGWRTSSKS---AVKNVDLWMELERLTLL--H-SI-EWRWVKAHAGDEYNEKADMLARGEA-----ERRM------VAPK---------------------------

>Verrucomicrobia | Verrucomicrobium | ZP_02925152

-----------------------------------MESVLPQVIIHTDGGCLG-NP-GV----GGWAAVLE---SCG----------RRK-----EISGGEPA-TTNNRMELRAAIEALSHL-------KKT------CAVEMHTDSQYVRNGIT-KWLAGWKKNGWKTASKQ---RVKNEDLWSTLDAAAQR--H-QV-SWHWVKGHAGHDDNERCDQLCGEAM-----EAVKKQHTRQQLAAALVAFKDTGR-----------------

>Tenericutes | Candidatus Liber | YP_003065209

----------------------------------MDSKHLREVHAYTDGACSG-NP-GP----GGWGVLLR---Y-K-G--------KEK-----IISGGEKE-TTNNRMELMAAIKALTAL-------KYP------CKVLLYTDSSYVHKGFS-QWIKKWQQNGWKTSDKK---TVKNIDLWMKFVEASAQ--H-KV-DLYWIKGHAGNQENEKVDRIARNAA-----VSFKNKI----------------------------------

>Tenericutes | Candidatus Solib | YP_827887

---------------------------------------MKKVQLITDGACLG-NP-GP----GGWSAILR---F-E-E--------QKK-----ELWGCEKQ-TTNNRMELTAAIEGLRAL-------REK------CQVEVVTDSEYVLKGIT-TWIDGWKRKGWMTAAKK---PVINQDLWKLLDEQVNR--H-QA-TWTWTKGHASHADNNRCDELATRAA-----REQS------KS-----------------------------

>Firmicutes | Faecalibacterium | ZP_05614459

------------MIRFPLDRTGKIALNRGIQKFKFREKQMKQVEVYTDGACSG-NP-GP----GGWGAVLR---Y-RFN-----GKVYEK-----ELSGGDAS-TTNNRMELTAFIEALRQL-------KEP------CEVRLCSDSQYVINGLEKGWARGWKRRGWKKSDGS---PALNPDLWEQALEQEAR--H-KI-TYVWVKGHAGHPENERCDQLAVAQSQAHGGRQGR-------------------------------------

>Deinococcus-Thermus | Thermus aquaticu | ZP_03496041

-----------------------------------MSLPLKRVDLFTDGACLG-NP-GP----GGWAALLR---Y-G-S--------QEK-----LLSGGEPC-TTNNRMELRAALEGLLAL-------REP------CQVHLHTDSQYLKRAFAEGWVERWQRNGWRTAEGK---PVKNQDLWQALLKAMEG--H-EV-AFHFVEGHSGHPENERVDREARRQA-----KAQPQVPCPPKEATLF-------------------------

>Deinococcus-Thermus | Thermus thermoph | YP_144822

----------------------------------MNPSPRKRVALFTDGACLG-NP-GP----GGWAALLR---F-H-A--------HEK-----LLSGGEAC-TTNNRMELKAAIEGLKAL-------KEP------CEVDLYTDSHYLKKAFTEGWLEGWRKRGWRTAEGK---PVKNRDLWEALLLAMAP--H-RV-RFHFVKGHTGHPENERVDREARRQA-----QSQAKTPCPPRAPTLFHEEA---------------------

>Actinobacteria | Gordonia bronchi | YP_003275599

-------------------------------MTESDSAGAPVVEISTDGACLG-NP-GP----GGWGAVLR---Y-R-G--------TEK-----RISGGEPN-STNNKMELTAAIEGLAAL-------TRP------STVILYTDSTYVRNGIT-KWVKGWQRNGWKTADKK---PVKNADLWRRLVEEEKV--H-TV-EWRWVKGHAGDQYNEIADELATTAA-----RQIA--------DSGKVAG----------------------

>Proteobacteria delta | Syntrophobacter | YP_844984

--------------------------MRGRRRMPETPAIRKHVEIFADGACRG-NP-GP----GGWGAVLR---YHG----------KEK-----ELSGYAEY-TTNNQMELAAVIQALRAL-------KEP------CRVTITTDSRYLRDGIS-LWIHKWKQNGWKTRVKT---DVRNKELWIALDEACLP--H-EI-DWQWVKGHSGHPENERCDALARAAI-----DRHL------REAATEE------------------------

>Proteobacteria delta | Desulfohalobium | YP_003197359

------------------------------------MSETSVVRLYTDGACLG-NP-GP----GGWAAVLL---YGG-E--------ARK-----ELSGGYAK-TTNNRMEMLALIEGLKVL-------KRP------CRVKVWTDSRYLHDGLTKGWLQKWQKNGWKTAAKK---PVKNKDLWQELAALTSR--H-QL-ELHWVRGHSGDPENERCDVLAKAAA-----NQPG-----LAKDPGHE------------------------

>Proteobacteria gamma | Xylella fastidio | ZP_00683618

---------------------------------------YEIDHAYTDGSCLG-NP-GP----GGWAVLLR---Y-K-N--------NEK-----ELVGGELD-TTNNRMELMAAIMALERL-------SEP------CQIKLHTDSQYVRQGIT-EWMSGWVRRGWKTAAGD---PVKNRDLWERLCAATQR--H-MV-EWCWVKAHNGDSDNERVDVLARGQA-----MAQR------STVASR-------------------------

>Proteobacteria gamma | Stenotrophomonas | YP_002027232

---------------------------------------MKTIEIHTDGSCLG-NP-GP----GGWAALLR---Y-K-G--------HER-----ELSGGEAH-TTNNRMELMAAISGLETL-------TEP------CDIVLYTDSQYVRQGLT-QWMPGWIRKNWKTAGGD---PVKNRELWERLHAATLR--H-QI-DWRWVKGHSGDPDNERVDTLARNAA-----IQIR------DSSPVN-------------------------

>Proteobacteria gamma | Xanthomonas oryz | YP_199680

---------------------------------------MKSIEVHTDGSCLG-NP-GP----GGWAALLR---Y-N-G--------REK-----ELAGGEAV-STNNRMELMAAIMALETL-------TEP------CEIVLHTDSQYVRQGIT-EWMPGWVRRNWKTAGGD---PVKNRELWERLHAATQR--H-RI-DWRWVKGHNGDPDNERVDVLARNQA-----TAQR---------DGRATS----------------------

>Proteobacteria gamma | Xanthomonas camp | NP_636365

---------------------------------------MKSIEVHTDGSCLG-NP-GP----GGWAALLR---Y-N-G--------REK-----ELAGGEAN-STNNRMELMAAIMALETL-------TEP------CQILLHTDSQYVRQGIT-EWMPGWVRRGWKTSGGD---PVKNRELWERLHAATQR--H-SI-EWRWVKGHNGDPDNERVDVLARNQA-----IAQR------GGLATS-------------------------

>Verrucomicrobia | Chthoniobacter f | ZP_03131837

------------MEIAASSGRAKRNILPVAPPSRYNDIILKKVTIHTDGACEG-NP-GP----GGWAAILE---Y-G-A--------VRK-----EISGGVIA-TTNNRMELTAALEALNRL-------KER------CAVDLFTDSEYLRNGIT-KWIFGWKAKGWK---KG---TIKNIDLWQALDAAASR--H-KV-EWHWVRGHAGHPLNERCDVLAVQET-----QKFRQSHTNAERKAARAAFLAERVGVPEQPELSSSLLK---

>Actinobacteria | Streptomyces sp. | ZP_05480970

----------------------------------MAEQTEEAVEIYTDGACSG-NP-GP----GGWGALLR---Y-G-K--------HER-----ELYGAEDTVTTNNRMELMAPIRALESL-------TRA------SVVRIYTDSTYVRNGIL-QWMPRWKKNGWQTQAKQ---PVKNADLWQRLDTACRQ--H-EV-EWLWVKGHAGLPENERADKLAVKGS-----QEAA------AAGVRRARG----------------------

>Proteobacteria zeta | Mariprofundus fe | ZP_01453598

------------------------------------MTEKPVVLAFTDGACSG-NP-GP----GGWGVLLR---M-G-K--------HEK-----EIYGGEAE-TTNQQMELQAAVEALKAL-------KQP------CKITVISDSKYVVQGMN-EWIHNWKKKGWKTVGKK---PVSNLERWQELDTLAAR--H-EV-QWQWVKGHAGHVENERADELARRGI-------PA-------------------------------------

>Proteobacteria gamma | Francisella phil | YP_001678245

---------------------------------MGIFTKKNNVIAYTDGACKG-NP-GI----GGWGAILS---Y-N-G--------VDK-----EISGAEKD-TTNNRMELMAAIKTLQAL-------KRK------CDITIYTDSKYLQNGIN-QWLANWKANGWKTAAKK---EVKNKDLWQELDSLTTK--H-NV-TWSWVKGHSGNQGNEKADELANKAI-----AELT------GK-----------------------------

>Proteobacteria beta | Nitrosomonas eut | YP_748363

---------------------------------MQLKSDMKRVEIFTDGACKG-NP-GP----GGWGVCLH---F-N-G--------ETR-----EFFGGEPV-TTNNRMELLAAIRALQELESLEDNGQQH------LQVQLHTDSQYVQKGIS-EWIHGWKKRGWRTADKK---PVKNEALWRELDDLSQR--H-QV-EWFWVRGHNGHAGNERADRLANQGV-----ESVL------SKKAD--------------------------

>Proteobacteria beta | Nitrosospira mul | YP_412312

----------------------------------MKAKLAEVVEIFTDGACKG-NP-GV----GGWGALLQ---Y-N-G--------HRR-----ELFGGEKM-TTNNRMELLAVIRALEAL-------TKP------CEVRLHTDSLYVQKGIS-EWIHAWKKRDWRTADKK---PVKNDDLWRELDLLTQR--H-KI-EWLWVRGHSGHDGNEYADMLANRGV-----QTAL------RGVSAN-------------------------

>Proteobacteria beta | Nitrosomonas sp. | ZP_05315423

----------------------------------MIGNISKVVEIYTDGACKG-NP-GI----GGWGALLR---Y-G-D--------HER-----EIFGGEKL-TTNNRMELLAAIRALESL-------KRP------CKIHLHTDSQYLQKGIS-EWLDSWKARNWCTADKK---PVKNEDLWKLLDQLTQQ--H-EI-EWCWVRGHSGHIDNERADQLANRGV-----EMII------SE-----------------------------

>Proteobacteria alpha | Erythrobacter li | YP_457199

---------------------------------------MKKVEIFTDGACKG-NP-GP----GGWGVLLR---M-G-K--------HEK-----ELSGGEPE-TTNNRMELRAAIEGLNAL-------IEP------CEVELYTDSKYVVDGIT-KWVHGWKKRGWVNASKK---PVRNDDLWHDLIEAELR--H-KV-TWHWVKGHNGHAENERADRLASEAA-----DLQS-------------------------------------

>Proteobacteria alpha | Erythrobacter sp | ZP_01864562

---------------------------------------MKKVEIFTDGACKG-NP-GP----GGWGALLR---M-G-R--------HEK-----ELSGGEPD-TTNNRMEMTAAIRALSAL-------IEP------CEVALHTDSKYLIDGIT-KWVHGWKKRGWVNASKK---PVRNADLWHELIELTAR--H-KV-DWFWVKGHSGHPENDRVDQLASDAA-----ERIA---------AGEAL-----------------------

>Proteobacteria alpha | Erythrobacter sp | ZP_01038775

--------------------------------MIGQSRPMKHVEIFTDGACKG-NP-GP----GGWGALLR---L-G-K--------HEK-----ELSGGEAD-TTNNRMELTAAIEGLRAL-------IEP------CKVDLYSDSKYVIDGIT-KWVHGWKKRGWVNASKK---PVRNSDLWHDLIDVTSR--H-EV-SWHWVKGHSGHTENERVDQLASDEA-----DRVA------RGE----------------------------

>Proteobacteria alpha | Zymomonas mobili | YP_163336

--------------------------------MPDSSTQDKIVMIATDGACKG-NP-GF----GGWGALLR---Y-Q-G--------HEK-----AISGSENP-TTNNRMELQAVIEALSCL-------KKP------CQIELSTDSKYVMDGLT-RWIHGWQKNGWLTAAKK---PVKNADLWKQLLALTRQ--H-DI-AWKWVKGHAGHPDNERADQLASDAA-----IALM--QQE-KA-----------------------------

>Proteobacteria alpha | Sphingopyxis ala | YP_616183

------------------------------------MSERRTVIVATDGACKG-NP-GP----GGWGAVLR---W-G-E--------VVK-----TLSGGEAD-TTNNRMELMAAIEALAAL-------KRP------CNVELSTDSVYVRDGIT-KWIFGWQKNGWKTAAKK---PVANADLWQRLIKEAAR--H-KV-EWLWVKGHAGHGDNELADQLASDAA--LKMARAR-------------------------------------

>Proteobacteria alpha | Novosphingobium | YP_496364

---------------------------------------MKHVEIFTDGACKG-NP-GK----GGWGALLR---M-G-E--------HEK-----EMAGSEKE-TTNNRMELMAAIRALEAL-------KQP------CRVTLHTDSKYVLDGIT-KWIFGWQKKGWKTADNK---PVKNEDLWRALVDAVRP--H-KV-EWVWVKGHDGHPENERVDKLASDAA-----LAA--------------------------------------

>Proteobacteria alpha | Sphingomonas wit | YP_001263184

------------------------------------MAELPLVEIATDGACKG-NP-GR----GGWGALLR---F-G-A--------TEK-----EMSGAENP-STNNRMELMAAIRALEAL-------KKP------CRVKLSTDSRYVMDGLT-KWIHGWRKNGWKTADKK---PVKNAELWQRLLDAAAP--H-RI-EWIWVKGHAGHPDNERADKLASDAA-----LGL--------------------------------------

>Actinobacteria | Frankia alni ACN | YP_714042

---------------------------------MAQRDGRVAVDIHTDGACSG-NP-GP----GGWGAVLR---Y-G-E--------HER-----ELHGGEPARTTNNRMELTAAIMALEAL-------TRP------SVVRLHTDSTYLRSGIT-TWIAGWRRNGWLTKDRT---PVRNADLWQRLEAAVAR--H-EV-EWLWVRGHAGDPGNERADALAARGL-----QEAR------QTPPPA-------------------------

>Proteobacteria gamma | Rickettsiella gr | ZP_02061806

------------------------------------MLKIPKIEIFTDGACRG-NP-GP----GAWAALLR---FQG----------KEK-----TLSGTEAS-TTNNRMELMAAIQALIAV-------KKP------CRIILSTDSKYVQKGIT-EWLPQWKRRAWLTANKK---PVKNSDLWKELALQAER--H-QI-SWEWVKGHSGHPENDRVDYLANVAL-----DKLL--GSF--------------------------------

>Proteobacteria gamma | Coxiella burneti | ZP_01947020

----------------------------------MAKQEQNIVYLYCDGACRG-NP-GP----GGWGVLLR---Y-N-Q--------HER-----QLHGGVAN-TTNNQMELTAAIEGLKSL-------KKP------CQVVVTTDSQYLRRGIT-EWLPVWKRRGWRTSNKK---PVKNQPLWETLEREVER--H-TI-VWHWVKGHSGHAENEIADELANRGI-----DEVL------KRGAR--------------------------

>Proteobacteria alpha | Rhodospirillum r | YP_428136

-----------------------------MSAAAGDEIKRVRVDMFTDGACSG-NP-GP----GGWGTILR---W-G-D--------TEK-----ELWGGETP-TTNNRMELMAVIRGLEAL-------RRP------VTVTIHTDSRYVHDGIT-GWIHGWKRNGWKTAAKK---PVKNEDLWRRLDAALGT--H-DI-SWQWVRGHSGHVENERADELARRGT-----SEAR--QGK-VDGQSSTIL----------------------

>Actinobacteria | Jonesia denitrif | YP_003160869

----------------------------MNNQSTSDDSDRATVTMWTDGACKG-NP-GV----GGWGVWMT---S-G-A--------HTK-----ELFGGENH-TTNNRMELMAVIEGLRAL-------KRP------CDVNLHVDSTYVMKGIT-SWIHGWKRNGWRTADKK---PVKNAELWRELDDQVTR--H-RV-TWTWVKGHSGDVGNDKADELANKGV-----ELVR--STT-SSTPTEPPSRHMPATKE--------------

>Actinobacteria | Cellulomonas fla | ZP_04368013

------------------------------MSPVSKTDDLPVVEMWTDGACKG-NP-GV----GGWGAWMR---F-G-D--------QER-----ELWGGEAA-TTNNRMELSAVIEGLRAL-------KRP------CRVTLHVDSTYVMNGLQ-KWLPNWKRNGWRTGDKK---PVKNQELWQALDTEVQR--H-HV-TWVWVKGHAGDPGNERADALANRGV-----DDVR------AGVR---------------------------

>Actinobacteria | Sanguibacter ked | YP_003315226

----------------MTQHSPTTTEPSTEPSTDPSTEADSAVEIWTDGACKG-NP-GV----GGWGAWLR---A-G-G--------HER-----ELFGGETV-TTNNRMELTAVIEALRAL-------KRP------CVVNLHVDSTYVMNGMS-KWIAGWKRNGWRTGDKK---PVKNVDLWQALDEQVAR--H-TI-TWTWVKGHSGDVGNEKADELANRGV-----AEVR--ARG--------------------------------

>Proteobacteria beta | Thiomonas interm | ZP_05498609

----------------------------------MTTESDNEIIIYTDGACKG-NP-GP----GGWGVVLR---S-G-A--------HEK-----TLHGGEPQ-TTNNRMELMAAIMALEAL-------KRP------SRVLLHTDSQYVLKGMT-EWIVGWKRRGWTTADKK---PVKNVDLWQRLEKAAAP--H-TL-RWVWVRGHTGDPGNEQADALANQGV-----EAAG------RG-----------------------------

>Proteobacteria beta | Methylobacillus | YP_545588

-------------------------------------MSSNVIEIYADGACKG-NP-GP----GGWGAWLS---F-A-G--------HEK-----ELWGGELV-TTNNRMELTAVIRALEAL-------KRQ------CSVRIYTDSVYVQKGIT-EWVHSWKARNWLTSDRK---PVKNVDLWKALDSLVQQ--H-QV-EWVWVKGHAGNVGNERADALANKGV-----DQVL------GREVV--------------------------

>Proteobacteria beta | Methylovorus sp. | YP_003051221

-----------------------------------MAVEEGCVVIYADGACKG-NP-GP----GGWGAWLA---M-G-G--------HEK-----EMCGGELL-TTNNRMELTAVIRALQAL-------KRP------CQVKIYTDSVYVQKGIT-EWMTGWKARNWRTSDKK---PVKNEDLWRELDQTVQP--H-NI-EWLWVKGHAGNAGNERADALANQGV-----LQAL------EAKERA-------------------------

>Proteobacteria beta | Bordetella avium | YP_787429

--------------------------------MSTANPPADLVEMWTDGACKG-NP-GP----GGWGVLMR---Y-G-S--------HEK-----TFFGGDPQ-TTNNRMEILAVVEGLRAL-------KRA------CTVVIHTDSQYVMKGMT-EWLPNWKRRGWLTADKK---PVKNAELWQLLDAQVAR--H-EV-RWQWVRGHNGDPGNEMADMLANQGV-----ASVA------RN-----------------------------

>Proteobacteria beta | Bordetella petri | YP_001629234

------------------------------MMQTDSNEDGPQVEMWTDGACKG-NP-GP----GGWGVLMR---A-G-A--------HEK-----TLHGGEAG-TTNNRMELLAVIEGLRTL-------KRP------CQVVIHTDSQYVMKGMT-EWLANWKRRGWLTADKK---PVKNAELWQALDEQVAR--H-KV-SWRWVRGHAGDPGNERADALANLGV-----ESLR------KRRAGA-------------------------

>Proteobacteria beta | Bordetella parap | NP_885986

------------------------------MQNLEGSGDGQQVEMWTDGACKG-NP-GP----GGWGVLMR---A-G-Q--------HEK-----TMHGGERQ-TTNNRMELMAVIEGLRAL-------KRP------CRVTIHTDSQYVMKGMT-EWLANWKRRGWRTADKK---PVKNVELWQALDEQVGR--H-QV-QWRWVRGHAGDPGNERADALANQGV-----EAAR------GR-----------------------------

>Proteobacteria beta | Polynucleobacter | YP_001797659

------------------------------MLHTKSSHHQPHIVIYTDGACKG-NP-GP----GGWGAVLR---S-G-G--------HEK-----HIHGGEKL-TTNNRMEICAVIFALKAL-------KQS------STVELWTDSQYVQKGVT-EWLEGWKKRGWKTASKD---PVKNADLWQELDTLIPD--H-DI-SWHWVRGHDGHPGNELADQLANKGV-----EEFL--P----------------------------------

>Proteobacteria beta | Polynucleobacter | YP_001155804

------------------------------MPHSKHPSSHPHIIIYTDGACKG-NP-GP----GGWGAVLR---S-G-S--------HEK-----HIHGGEKL-TTNNRMEICAVIFALKAL-------KQR------SSVELWTDSQYVQKGVT-EWLEGWKKRGWKTASKD---PVKNADLWQELDTLLPD--H-DI-SWHWVRGHNGHPGNELADALANKGV-----EEFL--P----------------------------------

>Tenericutes | Candidatus Accum | YP_003169109

------------------------------------MTDEVVITIFADGGCRG-NP-GP----GGWGVVLQ---A-G-E--------HEK-----ELWGGEPD-TTNNRMEMTAAIRALEAL-------KRP------ASVRLHTDSQYLQKGIS-EWIHNWKRNGWRTADKK---PVKNADLWQRLDELAGE--H-RI-QWCWVKGHAGHSGNERADALANRGM-----DELQ--RTA-NRRPLAGDGS---------------------

>Proteobacteria beta | Neisseria mening | YP_002343102

--------------------------------------MNQTVYLYTDGACKG-NP-GA----GGWGVLMR---Y-G-S--------HEK-----ELFGGEAQ-TTNNRMELTAVIEGLKSL-------KRR------CTVIICTDSQYVKNGME-NWIHGWKRNGWKTAAKQ---PVKNDDLWKELDALVGR--H-QV-SWTWVKGHAGHAENERADDLANRGA-----AQFS-------------------------------------

>Proteobacteria beta | Neisseria mucosa | ZP_05977102

--------------------------------------MDDTVYLYTDGACKG-NP-GA----GGWGVLMR---Y-R-N--------HEK-----ELCGGEAE-TTNNRMELTAVIEGLKAL-------KRP------CRVVICTDSQYVKNGME-GWIHGWKKNGWKTAAKK---PVKNDDLWKELDALSHK--H-EL-QWTWVKGHAGHSENEKADALANQGA-----ARFL------QSSS---------------------------

>Proteobacteria beta | Neisseria flaves | ZP_04758492

------------------MPIFQTALCYDTALFDKDMPMDKPVYLYTDGACKG-NP-GA----GGWGVFMR---Y-G-T--------HEK-----ELFGGEAE-TTNNRMELTAVIEGLKSL-------KRR------CQVVICTDSQYVKNGME-SWIHGWKKNGWKTAAKK---PVKNDDLWKELDSLVQQ--H-DV-RWTWVKGHAGHPENEKADELANQGA-----AKFA-------------------------------------

>Tenericutes | Candidatus Nitro | ACE75583

-----------------------------------------MIEIYTDGACSG-NP-GP----GGWGALLR---I-D-N--------AET-----EMCGGDPA-TTNNRMELLAVIEALQSL-------TQP------VEARVYTDSQYVQKGIS-EWIHSWKRRGWKTAGKE---PVKNEDLWRRLDTLASG--H-KL-EWHWVRGHNGHPENERVDALARAGL-----EQSR--RAG-KTVGGTRQSLF--------------------

>Proteobacteria beta | Thiobacillus den | YP_315421

-------------------------------------MTADIIYIYSDGACKG-NP-GA----GGWGALLV---A-G-G--------HRK-----EISGGEPN-TTNNRMEMTAVIRALELL-------KRP------STVEVHTDSQYVQKGVS-EWLPGWKRRNWRTADGK---PVKNQDLWQQLDALSQQ--H-RI-VWKWVRGHAGHPENERADVLANQGV---------------LQARQY-------------------------

>Proteobacteria beta | Rhodoferax ferri | YP_522728

---------------------------------------MNAVEIYTDGACKG-NP-GP----GGWGAFLK---S-A-D--------SQK-----ELFGGELG-TTNNRMEMTAVIEALAAL-------KRP------CQVTLHVDSQYVLKGMT-EWLAGWKARGWKTAAKQ---PVKNVDLWQRLDELVSTSGH-RI-DWRWVRGHNGDPGNEHADMLANRGV-----ELAL------RQR----------------------------

>Proteobacteria beta | Curvibacter puta | CBA29144

-----------------------------------MTEVLTKVVVYTDGACKG-NP-GP----GGWGVLLR---SAD-G--------TEK-----ELFGGELG-TTNNRMEMMAVIEALSAL-------KRP------CQITLHIDSQYVLKGIT-EWLQGWKAKGWKTASKQ---PVKNVDLWQRLDALVSGAGH-TI-DWRWVKGHAGDPGNERADGLANRGVSWHCVNAPE------CCPAAHHPPM---------------------

>Proteobacteria beta | Variovorax parad | YP_002944233

---------------------------------------MNEVVIYTDGACKG-NP-GP----GGWGAWLK---S-G-A--------TEK-----ELFGGELN-TTNNRMELTAVIEGLAAL-------KRP------CKVILYLDSQYVRMGIT-EWIRGWKAKGWRTSTKQ---PVKNVELWQKLDKLVAEGGH-VI-EWRWVKGHSGDVGNERADMLANKGV-----DKAL------GRI----------------------------

>Proteobacteria beta | Polaromonas sp. | YP_549102

-----------------------------MTDTQAGTTTQTQVVIYTDGACKG-NP-GP----GGWGVLLA---M-G-D--------TEK-----ELFGGEPV-TTNNRMEMTAVIEALAAL-------KRP------CRVTLYLDSEYVRKGIT-EWIHGWKARGWRTAAKA---PVKNVDLWQRLDALVTSSGH-KI-DWRWVKGHNGDPGNERADALANQGV-----ERAL------GRR----------------------------

>Proteobacteria beta | Polaromonas naph | YP_981914

---------------------------MTDSAAEPTISQPQHVVIYTDGACKG-NP-GP----GGWGALLA---S-G-G--------TEK-----EIFGGEMG-TTNNRMEMTAVIEALAAL-------KKP------CTVTLYLDSQYVLKGIT-EWIHGWKARGWRTAAKA---PVKNVDLWQRLDALLVSSGH-SI-DWRWVRGHNGDPGNERADALANKGV-----ERAL------GRL----------------------------

>Proteobacteria beta | Acidovorax delaf | ZP_04763296

---------------------------------------MNQIEIYTDGACKG-NP-GP----GGWGALLR---A-G-A--------TEK-----ELFGGELG-TTNNRMELMAVIEALSAL-------KRP------CAVTLYLDSEYVRKGIT-EWIHGWKARGWRTAAKQ---PVKNVELWQRLDALVTTAGH-RI-DWRWVRGHSGDPGNERADALANRGV-----DKAL------GRG----------------------------

>Proteobacteria beta | Verminephrobacte | YP_995394

---------------------------------------MNQVEIYTDGACKG-NP-GP----GGWGVLLR---S-G-P--------TEK-----ALFGGALG-TTNNRMELMAVIEALSAL-------QRP------CAVTLYLDSEYVRKGIT-EWIHGWKAKGWRTAARQ---PVKNVDLWQRLDALVSTGGH-RI-EWRWVKGHSGDPGNERADALANRGV-----DQAL------GRGALASAE----------------------

>Proteobacteria beta | Comamonas testos | YP_003277678

---------------------------------------MNQVVIYTDGACKG-NP-GP----GGWGALLQ---A-G-S--------AQK-----ELFGGELG-TTNNRMELKAVIEALSAL-------KRP------CDVVLYLDSQYVRKGIT-EWIQGWKAKGWVTASKE---PVKNVELWKQLDALVQGSGH-RI-DWRWVKGHAGDPGNERADALANKGV-----ELAL------KKG----------------------------

>Proteobacteria beta | Delftia acidovor | YP_001565909

---------------------------------------MNQVVIYTDGACKG-NP-GP----GGWGVVLE---S-G-S--------ARK-----ELFGGELN-TTNNRMEMMAVIEALSAL-------RRP------CDVVLYIDSQYVLKGIT-EWIHGWKAKGWKTASKE---PVKNVELWQRLDALVQGGGH-RI-DWRWVKGHAGDPGNERADALANKGV-----DQAL------GR-----------------------------

>Proteobacteria beta | Acidovorax sp. J | YP_986009

---------------------------------------MNQVVIYTDGACKG-NP-GP----GGWGAVLR---S-G-T--------LEK-----ELFGGELG-TTNNRMELMAVIQALGAL-------KRP------CQVALYLDSQYVRQGIT-EWIHGWKKKGWRTAAGQ---PVKNVELWQRLDELAHQAGH-RI-EWHWVRGHAGDPGNERADMLANKGV-----EQVL------GR-----------------------------

>Proteobacteria beta | Acidovorax citru | YP_970999

---------------------------------------MNQVVIYTDGACKG-NP-GP----GGWGVVLR---S-G-A--------LEK-----ELFGGELG-TTNNRMELLAVIEALGAL-------KRP------CAVTLYLDSQYVRKGIT-EWIQGWKKKGWRTASGQ---PVKNVELWKRLDDLVAGGGH-VI-DWRWVKGHAGDPGNERADALANKGV-----DKAL------GRA----------------------------

>Proteobacteria beta | Leptothrix cholo | YP_001791002

-------------------------MSIESTAGPQAVIAKPEVVIYTDGACKG-NP-GP----GGWGAWLV---S-G-G--------HEK-----ELCGGEAN-TTNNRMEMMAVIEALASL-------KRS------CRITVYTDSAYVQNGIS-SWIHGWKRRGWKTADNK---PVKNVDLWQRLDALSTL--H-QI-EWRWVKGHAGDPGNERADALANRGV---EVARAR-------------------------------------

>Proteobacteria beta | Aromatoleum arom | YP_160719

--------------------------------------MTDQIEIFTDGACSG-NP-GP----GGWGAILR---S-G-A--------HEK-----EIWGGEPH-TTNNRMELLAVIRALELL-------KRP------VVARVHTDSQYVQKGIS-EWIHGWKARGWKTAAKA---PVKNEDLWRALDEAASR--H-QV-QWVWVRGHAGHVENERADELARRGV-----DAVR------RQGAAVAG-----------------------

>Proteobacteria beta | Azoarcus sp. BH7 | YP_933559

---------------------------------------MEEVDIYTDGACSG-NP-GP----GGWGAILR---S-N-G--------HEK-----EIWGGEPQ-TTNNRMELIAVIRALEAL-------KRP------VAARVHTDSQYVQKGIS-EWIHGWKARGWKTASKE---PVKNADLWRTLDEVAGR--H-QV-KWLWVRGHAGHVENERADALARRGA-----EAAR------KQGTVVTN-----------------------

>Proteobacteria beta | Thauera sp. MZ1T | YP_002355924

---------------------------------------MEEVDIYTDGACSG-NP-GP----GGWGAILR---S-G-S--------HEK-----EIWGGEPA-TTNNRMELLAVIRALDAL-------KRP------VAARVHTDSQYVQKGIS-EWIHGWKARGWKTASKE---PVKNADLWRALDDAASR--H-QV-KWLWVRGHNGHPENERADALARRGV-----DAVR------KSGAAVQC-----------------------

>Proteobacteria beta | Oxalobacter form | ZP_04579209

---------------------------------------MNEVEIYTDGACRG-NP-GP----GGWGVWMI---A-G-G--------HEK-----ELFGGDAD-TTNNRMELMAVIEALRAL-------KRP------CKVVLHTDSQYVQKGIS-EWIHKWKARGWRTADKK---LVKNVDLWMELDQARAQ--H-DI-DWRWIKGHAGHEGNEKADQLANKGV-----DSVL-------------------------------------

>Proteobacteria beta | Oxalobacter form | ZP_04577078

----------------------------------MDSEKMSEVEIYTDGACRG-NP-GP----GGWGVWLR---A-N-G--------HEK-----ELFGGDAD-TTNNRMELTAVIEALRVL-------KRP------CRVVLHTDSQYVQKGIT-EWIHKWKERGWRTSDRK---LVKNVDLWMELDEATRR--H-DI-RWRWVKGHAGHEGNEKADQLANRGV-----DSVL-------------------------------------

>Proteobacteria beta | Burkholderia phy | YP_001857103

-------------------------------------MSSDLIEIFTDGACKG-NP-GP----GGWGALLR---Y-G-T--------QEK-----ELFGGEAN-TTNNRMELMAVIAALEAL-------KRP------CKAVVHTDSQYVQKGIS-EWIHGWKKKGWVTAARA---PVKNADLWKRLDALTQQ--H-QL-EWRWVKGHAGHPENERADALANRGV-----ASLA--DL---------------------------------

>Proteobacteria beta | Burkholderia gra | ZP_02886594

-------------------------------------MTANIIDIYTDGACKG-NP-GP----GGWGALLR---F-G-D--------QEK-----ELFGGEAN-TTNNRMELMGVISALEAL-------KRP------CKAVVHTDSQYVQKGIS-EWIHGWKKKGWVTAAKQ---PVKNADLWKRLDALVAQ--H-EI-EWRWVRGHNGHPENERADQLANRGV-----ASLA--EL---------------------------------

>Proteobacteria beta | Burkholderia glu | YP_002911003

-------------------------------------MTLQLIDIYTDGACKG-NP-GP----GGWGALLR---F-G-D--------QEK-----ELFGGEAG-TTNNRMELLAVIRALEAL-------KRP------CRVIVHTDSQYVQKGIS-EWIHGWKKKGWVTAAKT---PVKNADLWKQLDALVGQ--H-EI-EWRWVKGHAGHAENERADALANRGV-----ESLS------QRA----------------------------

>Proteobacteria beta | Burkholderia tha | ZP_02463089

-------------------------------------MTLQTIDIYTDGACKG-NP-GP----GGWGALLR---Y-G-T--------QEK-----ELFGGEAG-TTNNRMELTAVIAALAAL-------KRP------CKVVVHTDSQYVQKGIS-EWIHGWKKKGWVTAAKT---PVKNADLWQRLDALVAQ--H-DV-EWRWVKGHAGHPENERADALANRGV-----ESLA------QA-----------------------------

>Proteobacteria beta | Burkholderia amb | ZP_02889023

-------------------------------------MTTDTIDIYTDGACKG-NP-GP----GGWGALLR---Y-G-D--------REK-----ELFGGEPN-TTNNRMELMGVIGALEAL-------KRP------CRVIVHTDSQYVQKGIS-EWIHGWKKKGWVTAAKT---PVKNADLWKRLDALVAQ--H-EI-EWRWVKGHAGHPENERADALANRGV-----ESLV--A----------------------------------

>Proteobacteria beta | Burkholderia ubo | ZP_02379873

-------------------------------------MTTDTIDIYTDGACKG-NP-GP----GGWGALLR---Y-G-D--------REK-----EMFGGEPN-TTNNRMELMAVIASLEAL-------KRE------CRVVVHTDSQYVQKGIS-EWIHGWKKKGWVTAAKT---PVKNADLWKRLDALVAQ--H-QV-EWRWVKGHAGHPENERADALANRGV-----ESLA--A----------------------------------

>Proteobacteria beta | Lutiella nitrofe | ZP_03697564

-------------------------------------MTQDIVEIYPDGACKG-NP-GP----GGWGVLLR---F-K-G--------REK-----ELFGGEQG-TTNNRMELTAVIEGLAQL-------KRP------CKVAVYTDSQYVQKGIS-EWIHGWKKRGWKTAAKE---PVKNADLWQKLDALQAG--H-QI-SWHWVKGHAGHEFNERADQLANRGV-----ETLS--A----------------------------------

>Proteobacteria gamma | Chromobacterium | NP_900926

------------------------------------MTTEDRVEIYTDGACKG-NP-GP----GGWGALMR---Y-K-G--------KEK-----ELFGGERG-TTNNRMEIMAVIRALAAL-------NRP------CKVVVYTDSQYVQKGIS-EWIHGWKARGWKTAAKE---PVKNADLWQQLDAERNR--HLDV-EWRWVKGHAGHEFNERADQLANKGV-----ESV--------------------------------------

>Proteobacteria beta | Janthinobacteriu | YP_001352901

---------------------------------------MDKIDIYSDGACKG-NP-GR----GGWGALLV---M-G-E--------REK-----EIFGGELD-TTNNRMELKAVIEALNLL-------TRP------CEVVVHTDSQYVQKGIS-EWIHGWKARGWKTAAKA---PVKNVDLWQALDAAQAR--H-KI-EWRWVRGHNGHAGNERADALANRGV-----EVAA-------------------------------------

>Proteobacteria beta | Herminiimonas ar | YP_001100572

---------------------------------------MEKIDIFTDGACKG-NP-GR----GGWGALLV---M-G-E--------REK-----ELFGGEPG-TTNNRMELKAVIEALNAL-------TRP------CEVIVHTDSQYVQKGIS-EWIHGWKARGWKTAARA---PVKNVDLWQALDAAQAR--H-QI-EWRWVRGHNGHVGNERADALANRGV-----ETVN--SN---------------------------------

>Proteobacteria beta | Ralstonia picket | YP_002981688

---------------------------------------MQEVTVYSDGACKG-NP-GL----GGWGTVLV---S-G-S--------HEK-----ELFGGEAL-TTNNRMELMAVIEAFRAL-------KRP------CRVQVYTDSQYVQKGIS-EWLAGWKARGWKTADKK---PVKNDDLWRTLDELVAG--H-EV-SWHWVKGHAGHPGNERADALANKGV-----EMAR------QAKA---------------------------

>Proteobacteria beta | Ralstonia metall | YP_584356

---------------------------------------MQEVTIYSDGACKG-NP-GP----GGWGAVLV---A-G-G--------HEK-----ELFGGESP-TTNNRMELMAVIEALRAL-------KRP------CIVNIYTDSQYVQKGIS-EWIHGWKARGWKTADKK---PVKNADLWQALDEAQKP--H-QI-TWHWVRGHNGHPGNERADALANRGV-----ASIN--T----------------------------------

>Proteobacteria beta | Ralstonia eutrop | YP_296396

---------------------------------------MQEVIIYSDGACKG-NP-GR----GGWGAVLV---A-G-T--------NEK-----ELFGGEAN-TTNNRMEMTAVIEALRAL-------KRP------CTVQVYTDSQYVQKGIS-EWLPGWKARGWKTADKK---PVKNADLWQELDTLVQP--H-KI-TWHWVRGHNGHPGNERADALANRGV-----ASLA--S----------------------------------

>Proteobacteria beta | Cupriavidus taiw | YP_002006001

---------------------------------------MQEVTIYSDGACKG-NP-GR----GGWGAVLV---A-G-T--------SEK-----ELFGGEPN-TTNNRMEMTAVIEALRAL-------KRP------CVVRVYTDSQYVQKGIS-EWLPGWKARGWKTADKK---PVKNADLWQALDTLAQA--H-QI-SWHWVRGHNGHPGNERADALANRGV-----ESIG--R----------------------------------

>Proteobacteria gamma | Cardiobacterium | ZP_05706412

--------------------------------------MSTPLLIYTDGACKG-NP-GI----GGWGVLMC---Y-G-E--------HRK-----TLNGAEAM-TTNNRMELTAAIEALRAV-------KRA------CPIVLTTDSSYVKNGIT-QWLAGWKRNGWKTADKK---AVKNVDLWQALDALVAQ--H-QI-EWQWIKGHSGHPGNEMADQLANEAI-----AELR------AKG----------------------------

>Actinobacteria | Tsukamurella pau | ZP_04025800

-----------------------------------MIIVADEIVIYTDGACLG-NP-GP----GGWGAVLR---F-G-E--------HTK-----ELYGAEKD-TTNNRMELMGAISALEAI-------TKP------FPVVLYTDSSYVKNGIT-KWVEGWKRNGWKTANKQ---PVKNVELWQRLDEVAAR--Y-EI-DWRWVKGHAGNEGNELADQLASRGA-----AEAR--DS---------------------------------

>Proteobacteria gamma | Beggiatoa sp. PS | ZP_02002985

-------------------------------------MNESIVEAFTDGACRG-NP-GP----GGWGVLLR---C-Q-N--------EEK-----QLYGGELN-TTNNRMELMAAIMALESL-------TRS------NHIRLTTDSEYVKKGIT-EWIENWIKRGWKRANNE---PVKNIDLWQRLHAVTQK--H-QV-DWQWIKGHSGHSENEQADSLANQGI-----DSVV------QS-----------------------------

>Proteobacteria beta | Limnobacter sp. | ZP_01915744

--------------------------------------------MYADGACKG-NP-GP----GGWGVFLQ---S-G-D--------HAK-----ELCGGELN-TTNNRMELTAVIEGLNAL-------KKR------CSIDVYTDSQYVRKGVL-EWMPKWKMNGWKTSDKK---PVKNADLWQILDEASVR--H-LV-RWHWVKGHSGNPGNEKADALANLGV-----EKAM------KQ-----------------------------

>Proteobacteria gamma | Reinekea blanden | ZP_01115650

---------------------------------------MKTVTLYTDGGCRG-NP-GP----GGWGAVLI---Y-G-D--------HEK-----KLKGSEPE-TTNNRMELLAAIEGLEAL-------KQA------VTVDLYTDSKYVQQGIT-QWIHNWKKNGWKTAGKK---PVKNQDLWQRLDSLMSK--H-EV-NWHWVKGHAGHKYNEIADELANQAM-----DEMV------RQ-----------------------------

>Proteobacteria alpha | Oceanicaulis ale | ZP_00957665

-------------------------------------MAENTIVIHTDGACSG-NP-GP----GGWGAILH---W-K-G--------HEK-----ELSGAEAE-TTNNRMELMAAIAALEAL-------KRR------STVRLVTDSTYVRDGVT-KWIHGWKRNGWKTAAKK---PVKNDDLWKRLDAIASK--H-DV-TWEWVKGHAGHPENERADQLARDAI-----ATLS------KG-----------------------------

>Proteobacteria alpha | BAL199 | ZP_02189003

---------------------------------MGSDVAAERVAIFTDGACSG-NP-GP----GGWGAVMC---W-R-G--------TEK-----ELSGAEPL-TTNNRMELMAAIASLEAL-------SRR------VPVDLTTDSTYVRDGIT-KWMKAWKARGWKTADKK---PVKNQDLWERLDAAAKA--H-DV-AWHWVKGHAGHPENERADELARMAI-----AAMR------EAAR---------------------------

>Proteobacteria alpha | Maricaulis maris | YP_757447

---------------------------------------MSTITIHTDGACSG-NP-GP----GGWGAILE---W-N-G--------HRK-----ELKGGEAD-TTNNRMEMMAAIQALEAL-------RKA-----DRSVILITDSVYLRDGIT-KWIHGWKKRGWKTADKK---PVKNVDLWQRLDELTRS--H-TI-DWRWVKGHAGDPGNERADELAREGL-----AEAR------GRQP---------------------------

>Actinobacteria | Nocardiopsis das | ZP_04333609

----------------------------MRNGDEVGQEPTQRVVIYTDGACSG-NP-GP----GGWGVWLR---Y-G-G--------HEK-----ELYGGEAQ-TTNNRMELMAAIRALESL-------RQP------LPVLVHTDSSYVRNGIT-SWLHGWKRRGWRTADKK---PVKNVDLWQRLDEVASR--Y-EV-EWRWVRGHSGDEGNERADALARRGR-----DEAA------GV-----------------------------

>Proteobacteria beta | Gallionella ferr | ZP_04831427

-------------------------------------MNSEIVEIFTDGACKG-NP-GV----GGWGALLR---S-K-G--------VQR-----ELFGGEAH-TTNNRMELMGAISALEAL-------TRR------CQVKLHTDSKYVLQGIT-TWLAGWKRAGWKTSSRQ---PVKNEDLWRRLDALVIQ--H-EI-EWVWVKGHSGHAGNEHADELANRGV-----AMIQ------EQANGML------------------------

>Proteobacteria gamma | Halothiobacillus | YP_003263698

-------------------MTIELKKGSADQQNADAQPATQTVHIWTDGACKG-NP-GP----GGWGALLR---Y-G-D--------TER-----ELCGGEAH-TTNNRMELLAAISALEAL-------KRP------CTVHLTTDSQYVRQGML-EWLPNWRKKNWRRADGQ---PVKNADLWARLDEAAQR--H-DM-HWHWIKGHAGHPENERADQLANQGT------PKG-------------------------------------

>Proteobacteria alpha | Bartonella bacil | YP_988727

-----------------------------------MLSETKVIEIYTDGACSG-NP-GL----GGWGAILR---W-N-S--------HER-----ELYGGKEY-TTNNQMELMAAICALNAL-------KES------CSIDLYTDSVYVRNGIS-LWLENWKKNNWRTASKS---PVKNMELWQALDGACAR--H-NV-RWHWVKGHAGHPDNERADALARKAI-----TEYR--------QNGYFKG----------------------

>Proteobacteria alpha | Bartonella graha | YP_002971483

-----------------------------------MATQQKVVEIYTDGACSG-NP-GI----GGWGAILR---W-N-G--------HER-----ELYGGKVH-TTNNQMELMAAICALKAL-------KEP------CLVDLYTDSVYVRNGIS-KWIEDWKKNNWRTASKN---PVKNMELWQALEDACSC--H-TV-RWHWVKGHAGHPENERADALARKAI-----SQYR-------ENGRFPA-----------------------

>Proteobacteria alpha | Bartonella tribo | YP_001609086

-----------------------------------MASQQKVVEIYTDGACSG-NP-GV----GGWGAILR---W-N-G--------HER-----ELYGGNAH-TTNNQMELMAAICALKAL-------KEP------CLVDLYTDSVYVRNGIS-KWIEGWKKNNWRTASKS---PVKNMELWQTLEDACSC--H-AV-RWHWVKGHAGHPENERADALARKAI-----AQYR-------ENGRFPT-----------------------

>Proteobacteria alpha | Bartonella hense | YP_033281

-----------------------------------MLHQQKVVEIYTDGACSG-NP-GV----GGWGAILR---W-N-G--------HER-----ELYGGEVQ-TTNNQMELMAALCALKAL-------KES------CSVDLYTDSVYVRNGIS-LWLKGWKKNNWQTVSKK---PVKNKELWQALEGVCSF--H-TI-RWHWIKGHTGHPDNERADALARKAI-----AEYR-------ENGCFSA-----------------------

>Proteobacteria alpha | Bartonella quint | YP_032046

-----------------------------------MLNQQKVVEIYTDGACSG-NP-GV----GGWGAILR---W-N-G--------HER-----ELYSGEVQ-TTNNRMELMAAICALKVL-------KEA------CSVDLYTDSVYVRNGIS-LWLERWKMNNWRTTSKK---TVKNIELWKALEDVCSL--H-TI-RWHWVKGHAGHPDNERADALARKAI-----TEYR--------KNGYFSA----------------------

>Proteobacteria gamma | Hahella chejuens | YP_433754

---------------------------------------MKTVEIYTDGACKK-NP-GP----GGWGAILI---Y-G-K--------NEK-----EIYGGELD-TTNNRMELMAAIEALRAL-------KQG------CKVELYTDSQYVRKGIT-EWMQNWIKKGWRTSGGD---PVKNVDLWQALDKERNK--H-DI-SWRWVKGHSGHPLNERADELANLGV-----KEAL-------GETG--------------------------

>Proteobacteria alpha | Rhodospirillum c | YP_002299808

-----------------------------------MSAEKLLVDIYTDGACSG-NP-GP----GGWGAILR---W-K-G--------TEK-----ELKGGERL-TTNNRMELMAAIQALEAL-------KRP------VTVRLHTDSQYVKNGIT-TWIHGWKKNGWKTAGRD---PVKNADLWQRLDELVGR--H-TV-EFHWVKGHAGHPENERADQLAREGM-----RDTL------AAPAA--------------------------

>Proteobacteria gamma | Sideroxydans lit | ZP_05337703

--------------------------------------MSDVVEIFTDGACKG-NP-GL----GGWGALLR---V-K-G--------KEL-----ELCGGEAH-TTNNRMELLAAISALEAL-------KRQ------CRVRLHTDSKYVQQGIS-EWVHNWKLRGWKTADKK---PVKNEDLWRRLDTLAEQ--H-HV-EWVWVKGHAGHDGNERADALANRGC-----ADVE------KHLKKS-------------------------

>Proteobacteria alpha | Methylocella sil | YP_002361589

--------------------------------------MPKPVVIFTDGACSG-NP-GP----GGWGAVMT---F-G-D--------HLK-----ELCGGEAA-TTNNRMELMAAIMALEAL-------TRP------CAVQLVTDSNYVKGGVT-TWLAGWKRNGWRTADKK---PVKNVDLWQRLEAAAEA--H-AI-EWRWVKGHAGDELNERADALARLGM-----APFL------SARKAATP-----------------------

>Proteobacteria alpha | Parvibaculum lav | YP_001411964

------------------------------------MSGEDIVEIYTDGACSG-NP-GP----GGWGVLMI---Y-K-D--------REK-----ELCGGEQA-TTNNRMELMAAIQALEAL-------KRD------AHVRIHTDSNYVKDGIT-KWIHGWKKNGWKNAAKQ---PVKNAELWRRLEAAIST--H-QV-SWHWVKGHSDHPENDRADALARQGM-----APYL------PSK----------------------------

>Proteobacteria gamma | HTCC5015 | ZP_05062252

---------------------------------------MTDVTLYTDGACKG-NP-GP----GGWGVLLI---Y-G-G--------HEK-----ELCGGEAE-TTNNRMELMAAIEGLNAL-------KRS------CRVALYTDSNYVRQGMT-QWLANWKKNGWRTAAKK---PVKNDDLWQALDAACER--H-EI-EWHWVKGHSGDPGNERADELANRGV----LSAQA-------------------------------------

>Proteobacteria alpha | Hyphomicrobium d | ZP_05377708

-----------------------------------MTAEAPKILIYSDGACSG-NP-GP----GGWGAVLI---S-G-K--------HRK-----EISGGEVL-TTNNRMELLAAISALEAL-------KKR------SEVALYTDSAYVKNGIT-GWVHGWKKNGWRTADKK---PVKNVELWQALDALRNK--H-DV-EWHWLKGHAGHPENERADELARQAM-----APFK--LAP-RPDASTKI-----------------------

>Proteobacteria alpha | Asticcacaulis ex | ZP_04769350

---------------------------------------MKKVTIYTDGACKG-NP-GK----GGWGAILT---F-G-P--------HEK-----ELYGFEAE-TTNNRMELMAVIMALEAL-------KEP------CEIDVHADSQYVLKGIK-EWIHGWKARGWKTADKK---PVKNDDLWIRLDAARQR--H-KI-HWHWVKGHAGHEMNERADGLANKAI-----TEAA------AAF----------------------------

>Proteobacteria alpha | Caulobacter sp. | YP_001686013

--------------------------------------MTPKLVIYTDGACRG-NP-GP----GGWGALLM---Y-G-D--------KKK-----EIMGGDLA-TTNNRMELMAAIQALEAL-------NKP------TKAELHTDSQYVMKGVT-QWIHGWKAKGWKTADKS---PVKNVDLWQRLDAARAR--H-EV-DWRWVKGHAGHVHNERADELARLGM-----LKTL------GERGSGKAV----------------------

>Proteobacteria alpha | Phenylobacterium | YP_002131727

--------------------------------------MTPEVVIYTDGACSG-NP-GP----GGWGAILI---H-G-E--------REK-----ELCGGEAA-TTNNRMELMAAIQALEAL-------KRP------CRVELHTDSQYVQKGIH-EWIHGWKKRGWLTADKK---PVKNDDLWKRLDAARLR--H-HV-DWRWVKGHAGHELNERADALARKGL-----SEAA------AARAAGGA-----------------------

>Proteobacteria alpha | Caulobacter segn | ZP_06122411

--------------------------------------MTPKVTIYTDGACKG-NP-GP----GGWGAILF---Y-G-D--------KKK-----EICGGEPG-TTNNRMELMAAIQALELL-------NRP------CKVELHTDSQYVMKGIQ-EWIRGWKARGWKTADKS---PVKNDDLWKRLDAARAR--H-DV-DWRWVKGHAGHPLNERADALANEGL-----RQAN------PRFG---------------------------

>Proteobacteria alpha | Brevundimonas su | ZP_06170982

---------------------------------------MSHVIIHTDGACKG-NP-GP----GGWGAIIQ---F-G-E--------KAK-----EMSGGEPL-TTNNRMELTAAIMALEAL-------TRP------CKIDLHTDSKYVMDGIT-GWIHGWKARGWKTADKK---PVKNDDLWKRLDVARTR--H-EV-KWHWVKGHAGHALNERADQLANRGI-----EEMR------AAKAKA-------------------------

>Proteobacteria alpha | Brevundimonas sp | ZP_05034100

------------------------------------MSPTDHVIIHTDGACKG-NP-GP----GGWGALLQ---TGG-G--------HEK-----ELWGGEPN-TTNNRMELMAAIMALEAL-------KRP------CRVELHTDSKYVMQGIT-EWMRGWKARGWLTADKK---PVKNADLWQRLDAARLK--H-DV-KWRWVKGHAGHELNERADQLANRGV-----ADLR------RV-----------------------------

>Proteobacteria alpha | Rhodobacterales | ZP_01741948

---------------------------------------MPDLFAYTDGACSG-NP-GP----GGWGVLLI---A-K-N---ADKVLREK-----ELCGGEQE-TTNNRMELMAAISALENL-------SRP------STLTIITDSVYVKNGVT-QWVHGWKRNGWKTASKK---PVKNEELWKRIDEAQAR--H-QV-TWKWIKGHAGHEENERADELARRGM-----APFK------K------------------------------

>Proteobacteria alpha | Rhodobacterales | ZP_01011925

---------------------------------------MVDLIAHTDGACSG-NP-GP----GGWGVLMQ---A-K-D---GGTVVKER-----TLSGGEPA-TTNNRMELMAAIMALETL-------ERA------SKITIVTDSAYVKNGVT-GWIHGWKRNGWRTANKK---PVKNVELWQRLDEAAKR--H-DV-EWRWIKGHAGHEENERADELAREGM-----APFK-------------------------------------

>Proteobacteria alpha | Rhodobacter sp. | ZP_05842972

---------------------------------------MTDLFAYTDGACSG-NP-GP----GGWGVLMQ---A-R-D---GAVVVKER-----TLSGGEAD-TTNNRMELMAAISALEAL-------KRD------AGIVIVTDSAYVKNGVT-TWMTGWKRNGWKTADRK---PVKNVDLWLRLDEAQAR--H-KV-EWRWIKGHAGHEENERADELARAGM-----APFK------KPKG---------------------------

>Proteobacteria alpha | Rhodobacter spha | YP_001044401

---------------------------------------MPDLYAYTDGACSG-NP-GP----GGWGVLML---A-R-E---GEAVVKER-----TLQGGEVL-TTNNRMELMAAISALEAL-------TRP------TEITIVTDSAYVKNGVT-TWIHGWKRNGWKTADRK---PVKNAELWERLDAAQQR--H-KV-VWRWIKGHAGHAENERADELARAGM-----APFK------TR-----------------------------

>Proteobacteria alpha | Oceanicola granu | ZP_01157443

---------------------------------------MAELFAYTDGACSG-NP-GP----GGWGAVLI---A-R-E---GGAVLKER-----ELSGGEAR-TTNNRMELMAAISALEAL-------ERP------SRLTMVTDSNYVKDGIT-SWIAGWKRRGWKTAAKK---PVKNEDLWRRLDEAAAR--H-QV-TWEWVKGHAGHPENERADELARAGM-----APFK------R------------------------------

>Proteobacteria alpha | Thalassiobium sp | ZP_05343008

---------------------------------------MPDLIAYTDGACSG-NP-GP----GGWGALMI---A-R-D---GDTVLKKR-----ELKGGEAH-TTNNRMELLGAINVLETL-------AKP------SVITIVTDSAYVKGGIT-EWIFGWKRRGWKTSTKK---PVKNEDLWKRLDEVTQR--H-TV-TWEWVKGHAGHPENERADELARAGM-----EPFK------PAK----------------------------

>Proteobacteria alpha | Loktanella vestf | ZP_01002514

---------------------------------------MADLYAYTDGACSG-NP-GP----GGWGALLI---A-R-D---GDKVVKER-----ALSGGEAD-TTNNRMELLAAISALETL-------GRA------TAITIVTDSAYVKDGIT-SWIHGWKRRGWKTSANK---PVKNEDLWRRLDSAVAQ--H-QV-RWEWVKGHAGHVENERADELARAGM-----APYK------P------------------------------

>Proteobacteria alpha | Roseobacter sp. | ZP_01749501

---------------------------------------MPDLFAYTDGACSG-NP-GP----GGWGALLV---A-R-D---GDKVLKER-----ELCGGEAD-TTNNRMELLAAISALETL-------DRS------TALTIVTDSSYVKDGIT-QWIHGWKARGWKTAAKK---PVKNEDLWKRLDEVTAR--H-DV-TWEWVKGHAGHPENEKADELARAGM-----EPFK------P------------------------------

>Proteobacteria alpha | Jannaschia sp. C | YP_508444

---------------------------------------MPDLVAYTDGACSG-NP-GP----GGWGALMR---A-K-D---GDTILKER-----ELKGGEAD-TTNNRMELLAAISALEAL-------DRP------STLTIITDSAYVKNGIT-GWMHGWKRNGWKTSTRK---PVKNVDLWQRLDEAQSR--H-TV-TWEWIKGHAGHEGNEKADELARAGM-----APFK------TGKRGKDG-----------------------

>Proteobacteria alpha | Paracoccus denit | YP_917380

---------------------------------------MNALFAWTDGACSG-NP-GP----GGWGVLMR---A-M-D---GDRMLKER-----ELSGGEAE-TTNNRMELMAAISALEAL-------TRP------SEITVTTDSAYVKNGVT-QWIHGWKKNGWRTADRK---PVKNADLWQRLDAAQAR--H-QV-RWEWIKGHAGHPENERADELARAGM-----APFK------PARVSG-------------------------

>Proteobacteria alpha | Dinoroseobacter | YP_001531532

---------------------------------------MPELFAYTDGACSG-NP-GP----GGWGALLI---A-R-D---GDTVVKER-----ALKGGEAE-TTNNRMELLAAIHALEAL-------ERP------ARLTVVTDSAYVKGGVT-GWIHGWKRNGWKTSTKK---PVKNEDLWRRLDAAQAR--H-EV-QWEWVKGHAGHPENERADALAREGM-----APFK------PGKSKAGR-----------------------

>Proteobacteria alpha | Rhodobacterales | ZP_05076900

---------------------------------------MAKLLAYTDGACSG-NP-GP----GGWGVLMR---A-M-D---GDEIVKHR-----ELSGGAEL-TTNNQMELMAAISALEVL-------ERA------SELTIITDSTYVKNGVT-GWIHGWKKNGWKTSAKK---PVKNVELWQRLDAAQAR--H-QV-TWEWVKGHAGHPENERADELARAGM-----APFK------KTA----------------------------

>Proteobacteria alpha | Roseobacter sp. | ZP_05102097

---------------------------------------MAKLIAYTDGACSG-NP-GP----GGWGALMR---A-M-E---DGKIVKER-----ELKGGEAA-TTNNRMELMAAISALEAL-------ARP------TEITIVTDSNYVKNGIT-NWIHGWKKNGWKNAAKK---PVKNAELWQRLDAANAR--H-SV-TWKWVKGHAGHPENERADELARAGM-----APFK------GK-----------------------------

>Proteobacteria alpha | Oceanibulbus ind | ZP_02152227

---------------------MTATRRAHKPRWKLPETTMPDLYAYTDGACSG-NP-GP----GGWGVLMR---A-M-D---GDKIVKER-----ELKGGEGQ-TTNNRMELMAAISALESL-------SRT------TEITIVTDSNYVKNGIT-GWIFGWKKNGWKNAAKK---PVKNAELWQRLDAANAR--H-NV-TWKWVKGHAGHPENERADELARAGM-----APFK------PGGKK--------------------------

>Proteobacteria alpha | Roseovarius nubi | ZP_00960363

---------------------------------------MVDLVAYTDGACSG-NP-GP----GGWGVLMQ---A-K-R---GAEVIKQR-----ELSGGEAL-TTNNQMELMAAITALETL-------EKP------STITIVTDSQYVKNGVT-GWIFGWKKNGWKTSAKK---PVKNVELWQRLDAAQAR--H-KV-TWEWVKGHAGHPENERADELAREGM-----APYK------PKAAK--------------------------

>Proteobacteria alpha | Roseobacter lito | ZP_02141318

---------------------------------------MPELFAYTDGACSG-NP-GP----GGWGVLLQ---A-K-E---GDRLVKER-----ALKGGEAH-TTNNRMELLAAINALESL-------SRA------STITIVTDSNYVKNGIT-GWIHGWKRNGWKNAAKK---PVANAELWQRLDEANAR--H-DV-TWKWVKGHAGHAENERADELARAGM-----APFK------P------------------------------

>Proteobacteria alpha | Roseobacter sp. | ZP_01055839

---------------------------------------MADLYAYTDGACSG-NP-GP----GGWGALLQ---A-K-D---GGSVIKEK-----ELKGGEAN-TTNNRMELLAAINALESL-------DRP------SALTVVTDSNYVKNGIT-GWIFGWKKNGWKNAAKK---PVKNAELWQRLDAAQSR--H-QV-TWEWVKGHAGHPENERADELARAGM-----APFK------KSKSKA-------------------------

>Proteobacteria alpha | Roseovarius sp. | ZP_01878558

---------------------------------------MPALFAYTDGACSG-NP-GP----GGWGVLMR---A-M-D---GDAILKER-----ELSGGEAD-TTNNRMELWAAIAALEAL-------SRP------STITIVTDSAYVKNGVT-GWMHGWKRNGWRTADKK---PVKNVELWQRLDEAQKR--H-TV-TWEWVKGHAGHPENERADELARAGM-----APYK------PSKAKG-------------------------

>Proteobacteria alpha | Sagittula stella | ZP_01744336

---------------------------------------MPDLYAYTDGACSG-NP-GP----GGWGVLLR---A-M-E---GDEVVKQR-----ELKGGERV-TTNNQMELMAAISALESL-------TKP------SRITVITDSQYVKNGVT-GWIFGWKKNGWKTAAKK---PVKNVELWQRLDAAQAR--H-DV-VWEWVKGHAGHPENERADELARAGM-----APFK------S------------------------------

>Proteobacteria alpha | Oceanicola batse | ZP_00998823

---------------------------------------MPDYFAYTDGACSG-NP-GP----GGWGVLLQ---A-K-D---GETVLKER-----DLKGGEAA-TTNNRMELLAAINALEAL-------GRS------TAITIVTDSAYVKNGVT-GWIHGWKRNGWKTAAKK---PVKNADLWQRLDEAQAR--H-DV-TWQWVKGHAGHPENERADELARAGM-----APFK-------------------------------------

>Proteobacteria alpha | Ruegeria pomeroy | YP_168415

---------------------------------------MPELFAYTDGACSG-NP-GP----GGWGVLLR---A-I-E---GETVLKER-----ELCGGEAE-TTNNRMELLAAINALETL-------ERP------SKITVVTDSAYVKNGVT-GWIFGWKRNGWKTAGKK---PVKNVELWQRLDLAQAR--H-DV-TWKWVKGHAGHPENERADELARAGM-----KPFK------PKKARA-------------------------

>Proteobacteria alpha | Roseobacter sp. | ZP_01901987

---------------------------------------MPDLFAYTDGACSG-NP-GP----GGWGVLLQ---A-I-E---GDTVLKER-----ELSGGEAE-TTNNRMELLAAINALETL-------AKP------SKITIVTDSAYVKNGVT-GWIHGWKRNGWKTAARK---PVKNVELWQRLDEAQAR--H-DV-TWEWVKGHAGHPENERADALARAGM-----APFK------PSA----------------------------

>Proteobacteria alpha | Rhodobacteraceae | ZP_05122674

---------------------------------------MPDLFAYTDGACSG-NP-GP----GGWGVLLR---A-M-D---GETVLKER-----ELKGGEAE-TTNNRMELLAAISALETL-------ERA------SDITIVTDSAYVKNGVT-GWIFGWKRNGWKTSNKK---PVKNVDLWQRLDEAQAR--H-QV-TWEWVKGHAGHPENERADELARAGM-----APFK------PGKAQA-------------------------

>Proteobacteria alpha | Silicibacter lac | ZP_05786713

---------------------------------------MPDLFAYTDGACSG-NP-GP----GGWGVLLR---A-V-D---GETVLKER-----ELNGGEAE-TTNNRMELLAAISALEAL-------ERP------SKITIVTDSAYVKNGVT-GWIHGWKRNGWKTASRK---PVKNVDLWQRLDEAQQR--H-DV-TWEWVKGHAGHPENERADELARAGM-----APFK------PKKART-------------------------

>Proteobacteria alpha | Ruegeria sp. TM1 | YP_614567

---------------------------------------MPDLFAYTDGACSG-NP-GP----GGWGALLR---A-M-D---GETVLKER-----ELKGGEKE-TTNNRMELLAAIHALESL-------ARP------SKITVVTDSAYVKNGVT-GWIFGWKKNGWKTSAKK---PVKNVELWQRLDAAQSR--H-DV-TWEWVKGHAGHPENERADELARAGM-----APFK------SSGKSSKG-----------------------

>Proteobacteria alpha | Citreicella sp. | ZP_05782407

---------------------------------------MPELFAYTDGACSG-NP-GP----GGWGVLMR---A-M-N---GEDIVKER-----ELKGGEAD-TTNNRMELLAAINALESL-------TRP------TTITVVTDSAYVKNGVT-GWIHGWKRNGWNTAAKK---PVKNAELWQRLDEAQRM--H-SV-TWKWVKGHAGHPENERADELARAGM-----APFK------PGGK---------------------------

>Proteobacteria alpha | Ruegeria sp. R11 | ZP_05089769

---------------------------------------MAELFAYTDGACSG-NP-GP----GGWGALLR---A-M-D---GDTVIKEK-----ELKGGEAE-TTNNRMELLAAIHALESL-------ARP------STITVVTDSAYVKNGVT-GWIHGWKRNGWKTASKK---PVKNVELWQRLDEAQRR--H-TV-TWEWVKGHAGHPENERADELARAGM-----APFK------QGKTKA-------------------------

>Proteobacteria alpha | Phaeobacter gall | ZP_02145641

---------------------------------------MAELFAYTDGACSG-NP-GP----GGWGVLLR---A-M-D---GETIVKEK-----ELSGGEAE-TTNNRMELLAAINALENL-------ARP------STLTVVTDSAYVKNGVT-GWIHGWKRNGWKTASKK---PVKNVELWQRLDEAQRR--H-TV-TWEWVKGHAGHPENERADELARAGM-----APFK------PGKAKA-------------------------

>Proteobacteria alpha | Rhodobacterales | ZP_05077818

---------------------------------------MPELFAYTDGACSG-NP-GP----GGWGVLLR---A-M-D---GDSIVKEK-----ELSGGEAE-TTNNRMELLAAINALESL-------ARP------STITVVTDSAYVKNGVT-GWIFGWKKNGWKTSNKK---PVKNVELWQRLDEAQRR--H-KV-TWEWVKGHAGHPENERADELARAGM-----APFK------KKKGA--------------------------

>Proteobacteria alpha | Roseobacter sp. | ZP_01753735

---------------------------------------MPDLYAYTDGACSG-NP-GP----GGWGVLLR---A-M-D---GEAIIKEK-----ELQGGEAE-TTNNRMELLAAINALESL-------ARS------STITVVTDSAYVKNGVT-GWIFGWKKNGWKTAAKK---PVKNVELWQRLDEAQSR--H-RV-TWEWVKGHAGHPENERADELARAGM-----APYK------KSKG---------------------------

>Proteobacteria alpha | Rhodopseudomonas | YP_484963

----------------------------MSGALSEAGAGPRPVVIHTDGACSG-NP-GP----GGWGAILK---F-G-D--------TEK-----ELKGGEAH-TTNNRMELLAAISALEAL-------TRP------CTVDLYTDSQYVKNGIG-SWIHNWKRNGWKTADKK---PVKNVDLWQRLDAALKS--H-QV-RWHWVKGHAGHDENERADQLARDGL-----TENR-----MKSRIG--------------------------

>Proteobacteria alpha | Rhodopseudomonas | NP_949605

----------------------------------MSEADQKPVIIHTDGACSG-NP-GP----GGWGAILK---F-G-D--------VEK-----ELKGGEPH-TTNNRMELLAAISALEAL-------TRP------CSVDLYTDSQYVKNGIG-SWIHNWKRNGWKTADKK---PVKNVDLWQRLDAALKT--H-SI-RWHWVKGHAGHAENERADQLARDGL-----TENR-----MKSRVK--------------------------

>Proteobacteria alpha | Rhodopseudomonas | YP_783034

------------------------------------MSTLPAVLIHTDGACSG-NP-GP----GGWGAILK---F-G-E--------REK-----ELKGGESH-TTNNRMELMAAISALEAL-------TKP------CSVDLHTDSQYVRNGIS-SWIHGWKKNGWKTADKK---PVKNVDLWQRLDAALKQ--H-EV-RWHWVKGHAGHAENERADQLARDGL-----SENR-----LKSRIG--------------------------

>Proteobacteria alpha | Rhodopseudomonas | YP_533921

------------------------------------MSALPAVRVHTDGACSG-NP-GP----GGWGAILK---F-G-E--------IEK-----QLKGGETH-TTNNRMELLAAISALEAL-------TKP------CTVDLYTDSQYVRQGIT-AWIHNWKRNGWKTADKK---PVKNVDLWQRLDAALKQ--H-DL-RWHWVKGHAGHDENERADQLARDGL-----IEHK-----LKSKIG--------------------------

>Proteobacteria alpha | Bradyrhizobium j | NP_767956

------------------------------------MSELPVVTIYTDGACSG-NP-GP----GGWGAILK---F-G-D--------KEK-----ELNGGERH-TTNNQMELMAAISALEAL-------KKP------CTVDLYTDSQYVRQGIT-GWIHGWKRNGWRTADKK---PVKNVELWQRLDAALKA--H-QV-RWHWVKGHAGHPENERADQLARDGI-----VKAR------LQQRVAE------------------------

>Proteobacteria alpha | Bradyrhizobium s | YP_001237112

------------------------------------MSELPTVSIYTDGACSG-NP-GP----GGWGAILR---F-G-D--------KEK-----ELKGGEPH-TTNNRMELMAAISALEAL-------KKS------CQVELYTDSQYVRQGIT-GWIHGWKRNGWKTADKK---PVKNAELWQRLDAALKP--H-KV-NWHWVKGHAGHAENERADQLARDGV-----AMAR------LQKNVRG------------------------

>Proteobacteria alpha | Nitrobacter sp. | ZP_01045333

----------------------------------MNSPALPHVTIFTDGACSG-NP-GP----GGWGAILR---F-G-D--------IEK-----ELKGGEPH-TTNNRMELLAAISALEAL-------KRP------ALVDLTTDSQYVRQGIM-SWIHNWKRNGWRTADKK---PVKNADLWQRLDAALQP--H-QV-RWHWIKGHDGHSENERADQLAREGV---AIARLK-------------------------------------

>Proteobacteria alpha | Nitrobacter hamb | YP_578927

----------------------------------MNSSALPHVTIFTDGACSG-NP-GP----GGWGAILR---F-G-E--------IEK-----ELKGGEPH-TTNNRMELLAAISALEAL-------KKA------ASVDLTTDSQYVRQGIT-SWIHNWKRNGWRTADKK---PVKNADLWQRLDTALQP--H-QV-RWHWIKGHAGHDENERADQLAREGV---ALARLK-------------------------------------

>Proteobacteria alpha | Oligotropha carb | YP_002290298

----------------------------------MSEPQRPHVVIFTDGACSG-NP-GP----GGWGAILR---F-G-E--------IEK-----ELKGGENP-TTNNRMELLAAISALEAL-------KRS------AIVDLTTDSQYVRQGIT-SWIFNWKKNGWRTSDKK---PVKNVDLWQRLDAALKP--H-EV-RWHWIKGHAGHAENERADELAREGL-----AENR-------------------------------------

>Proteobacteria alpha | Beijerinckia ind | YP_001833435

--------------------------------------MSGRVTIYTDGACSG-NP-GP----GGWGAILM---F-G-Q--------HEK-----ELSGGEAQ-TTNNRMELTAAIRALEAL-------TRP------CAVDLHTDSNYLRGGVT-SWIKGWKKNGWRTADKK---PVKNVELWQELDQLAAS--H-EI-AWHWVKGHAGHPLNERADALARQGM-----APFR------GGARIHPH-----------------------

>Proteobacteria alpha | Azorhizobium cau | YP_001524382

---------------------------------------MSRVEIWTDGACSG-NP-GP----GGWGAILR---S-G-P--------HEK-----ELKGGEAL-TTNNRMELMAAISALEAL-------KKP------CGVDLHTDSEYLRNGIT-KWMFGWKRNGWRTADKK---PVKNQDLWERLDAALHS--H-DI-AWHWVKGHAGNELNERADQLARDGM-----APFK------MGGRVA-------------------------

>Proteobacteria alpha | Xanthobacter aut | YP_001417257

---------------------------------------MKEVAVFTDGACSG-NP-GP----GGWGAILR---F-G-A--------HEK-----ELSGGEAL-TTNNRMELMGAIAALEAL-------KEP------CTVDLHTDSNYLKDGVT-KWMHGWKRNGWRTADKK---PVKNQDLWERLDAALKR--H-TL-RWHWVKGHAGHAENERADELARAGM-----APFK------LKGRLSG------------------------

>Proteobacteria alpha | Sphingomonas sp. | ZP_01303532

------------------------------------MSDLPQVEIFTDGACKG-NP-GP----GGWGAVLR---F-G-D--------TEK-----EISGGEAQ-TTNNRMEMTAALEALNLL-------KKP------CAVTLYTDSKYVMDGIT-KWVFGWQKKGWRTADNK---PVKNVEIWQNLVKAAAR--H-QM-TWKWVKGHAGHPENERADQLASAAA-----ETFR------R------------------------------

>Proteobacteria alpha | Fulvimarina pela | ZP_01439595

------------------------------------MSKENRVEIYTDGACSG-NP-GP----GGWGVLLR---F-G-E--------HSK-----ELKGGEAN-TTNNRMELLAAIEALSAL-------KRP------CAIDLHSDSSYMRDGIM-KWIHGWKKNGWKTADKK---PVKNAELWQRLDEERSR--H-DV-TFHWVKGHAGHEGNERADQLANDGM-----EPFK------KKARSA-------------------------

>Proteobacteria alpha | Aurantimonas man | ZP_01228016

------------------------------------MSAEGRVEIHTDGACSG-NP-GP----GGWGAILR---F-N-G--------NEK-----ELKGGEEH-TTNNRMELLAVIEALTAL-------KRS------CPVDIYSDSQYMRDGIT-KWIHGWKRNGWKTADKK---PVKNAELWQKLEEEKGR--H-DV-TFHWVKGHAGDEMNERADQLARDGM-----EPFK------RRKRSA-------------------------

>Proteobacteria alpha | Labrenzia alexan | ZP_05115201

------------------------------------MSQENRVTIYTDGACSG-NP-GP----GGWGVIMR---F-G-E--------HER-----ELKGGEVE-TTNNRMELTAAIEALNAL-------KRP------CVVDLYTDSTYVRSGIS-EWMYGWKRKNWKTAANK---PVKNADLWQALDAARER--H-DV-TWHWVKGHAGHPDNERADELARGGM-----EPFK------KNS----------------------------

>Proteobacteria alpha | Labrenzia aggreg | ZP_01548145

-----------------------------------MTENTNRVTIYTDGACSG-NP-GP----GGWGVILR---F-G-E--------HEK-----ELCGGEAE-TTNNRMELMAAIEALNAL-------KRP------CAVDLYTDSTYVRSGIK-EWMYGWKRKNWRTAANK---PVKNADLWQALDAAKER--H-DV-TWHWVKGHAGHPDNERADELARGGM-----APYK------NGEKTNPV-----------------------

>Proteobacteria alpha | Chelativorans sp | YP_673315

---------------------------------------MKRIEIFTDGACSG-NP-GP----GGWGAILR---Y-N-G--------TEK-----ELYGGEAD-TTNNRMELTAAIEALEAL-------KEP------CEVDLHTDSNYLRDGIS-GWIEGWKRNGWRTADRK---PVKNAELWQALDEARRR--H-KV-HWHWVRGHAGHPENERADALARAGM-----APFK------KKKGGDTASSEEGSARRR-------------

>Proteobacteria alpha | Ochrobactrum ant | YP_001369151

---------------------------------------MKRIEAYTDGACSG-NP-GP----GGWGAILR---W-N-D--------NVK-----ELKGGEAD-TTNNRMELMAAISALSAL-------KEP------CEVDLYTDSVYVRDGIS-GWIEGWKRNGWKTAAKK---PVKNAELWQALDEARKP--H-KV-NWHWVKGHAGHPENERADELAREGM-----EPFK------YGGRKSLKVQ---------------------

>Proteobacteria alpha | Brucella meliten | NP_540374

---------------------------------------MKRIEAYTDGACSG-NP-GP----GGWGALLR---W-N-G--------NEK-----ELKGGEAE-TTNNRMELMAAISALSAL-------KEP------CEVDLYTDSVYVRDGIS-GWIEGWKRNGWKTAAKK---PVKNAELWQALDEARKA--H-KV-TWHWIKGHAGHPENERADELARAGM-----EPFK------YAGHRTLKVK---------------------

>Proteobacteria alpha | Mesorhizobium op | ZP_05808546

--------------------------------------MSKNVEIFTDGACSG-NP-GP----GGWGAILR---F-N-G--------ATK-----ELSGGEAE-TTNNRMELLAAISALNAL-------KEP------CTVELHTDSKYVMDGIS-KWIHGWKKNGWKTADKK---PVKNGELWQALDEANRR--H-KV-TWNWVKGHDGHVENERADELARQGM-----APFK------KGPFKPAAPAKPSAPAKQPAATKARRSTQSY

>Proteobacteria alpha | Hoeflea phototro | ZP_02167424

---------------------------------------MKKVEVFTDGACSG-NP-GP----GGWGAILR---Y-G-E--------IEK-----EMSGGEAA-TTNNRMELLAAINALNAL-------KGA------CEVELHTDSKYVMDGIS-KWIHGWKKNGWKTAAKK---PVKNAELWQALEEARKP--H-KV-NWHWVKGHAGHPENERADELARFGM-----EPYK------NKTAPRRSAG---------------------

>Proteobacteria alpha | Agrobacterium vi | YP_002548759

---------------------------------------MKHVDIFTDGACSG-NP-GP----GGWGAVLR---Y-G-E--------VEK-----DLCGGEAD-TTNNRMELLAAITALNTL-------KTP------CEVDLHTDSKYVMDGIS-KWIFGWKKNGWKTADKK---PVKNGELWQQLDAANQR--H-KV-TWHWVKGHAGHPENERADELARKGM-----EPFK------KGGAGSKPIL---------------------

>Proteobacteria alpha | Rhizobium sp. NG | YP_002825084

---------------------------------------MKHVDIFTDGACSG-NP-GP----GGWGAVLR---Y-G-E--------VEK-----EMFGGEAE-TTNNRMELMAAISALNAL-------KQP------CEVDLHTDSKYVMDGIS-KWIHGWKRNGWKTGDRK---PVKNGELWQALDEARDR--H-QV-TWHWVKGHAGHPENERADELARKGM-----EPFK------KVRRTDAVK----------------------

>Proteobacteria alpha | Sinorhizobium me | NP_385020

---------------------------------------MKHVHIFTDGACSG-NP-GP----GGWGAVLR---Y-G-D--------VEK-----EMSGGEAE-TTNNRMELLAAISALNAL-------RQP------CEVDLHTDSKYVMDGIS-KWIHGWKRNGWKTGDRK---PVKNGELWQALDEARNR--H-NV-TWHWVKGHAGHPENERADELARKGM-----EPFK------KARRADAVK----------------------

>Proteobacteria alpha | Agrobacterium tu | NP_353800

---------------------------------------MKHVDIFTDGACSG-NP-GP----GGWGAVLR---Y-G-E--------TEK-----ELSGGEAD-TTNNRMELLAAISALNAL-------KSP------CEVDLYTDSAYVKDGIT-KWIFGWKKKGWKTADNK---PVKNVELWQALEAAQER--H-KV-TLHWVKGHAGHPENERADELARKGM-----EPFK------RR-----------------------------

>Proteobacteria alpha | Rhizobium etli K | ZP_03502453

---------------------------------------MKHVDIFTDGACSG-NP-GP----GGWGAVLR---Y-G-D--------VEK-----ELCGGEAD-TTNNRMELMAAISALQAL-------KTP------CEVDLYTDSAYVKDGIS-KWIFGWKKNGWKTSDKK---PVKNAELWQALEEARNR--H-KV-TLHWVKGHAGHPENERADELARRGM-----EPFK------KGKAVSV------------------------

>Proteobacteria alpha | Agrobacterium ra | YP_002543663

---------------------------------------MKQVDIFTDGACSG-NP-GP----GGWGAVLR---Y-G-D--------KEK-----ELFGGEAE-TTNNRMELMAAISSLNAL-------KSP------CEVNLYTDSKYVMDGIS-KWIFGWKKNGWKTADKK---PVKNAELWQALEEARNR--H-QV-TLHWVKGHAGHPENERADELARKGM-----EPFK------RK-----------------------------

>Proteobacteria gamma | Chromohalobacter | YP_573993

------------------------------MTDSHATGDMPRVTIYTDGACRG-NP-GP----GGWGAVLR---Y-G-Q--------HEK-----TLKGGEAV-TTNNRMELMAAIQALRTL-------TRA------CDVALWTDSEYLRKGIT-EWIHGWVKRGWKTAAKQ---PVKNAELWRELLAETQR--H-RI-EWHWVKGHSGHEGNELADTLANAAT-----DEIQ------AAKRQAMAGEQ--------------------

>Proteobacteria gamma | Allochromatium v | ZP_04772617

--------------------------------------MSDCVEAFTDGACKG-NP-GP----GGWGVLLR---W-G-E--------VEK-----ELHGGERE-TTNNRMELMAVIMALEAL-------KRP------TPIRITTDSQYVKRGVG-EWMPRWKRNGWRTADRQ---PVKNRDLWERLDRALGQ--H-EV-SWRWVKGHAGHAENERADWLANLGV---------------PTTGGR-------------------------

>Proteobacteria gamma | Legionella longb | EEZ95027

----------------------------------------MIVEIYTDGACKG-NP-GP----GGWGVLLR---C-K-G--------QEK-----TLHGGEAH-TTNNRMELMAAIKGLEAL-------KRS------CIVDLYTDSQYLRQGML-DWLPNWKMKGWRNSKKE---PVKNADLWMMLDELASR--H-QI-NWHWVKGHSGHLENELVDALANQAI-----EELR------E------------------------------

>Proteobacteria gamma | Nitrosococcus oc | YP_344792

--------------------------------------MTEIVEIFTDGACRG-NP-GP----GGWGALLC---Y-Q-G--------REK-----TLSGAESK-TTNNRMELMAAIRALETL-------KRP------CRVHLTTDSQYLRQGIT-CWLSNWKRRGWKTANRQ---PVKNIDLWQRLDQVAAQ--H-RI-EWFWVRGHEGHPGNERADALARSAI---TNGEEK-------------------------------------

>Proteobacteria gamma | Bermanella maris | ZP_01308670

--------------------------------------MSQIVEMFTDGACKG-NP-GP----GGWGVLLR---Y-G-A--------HEK-----ELFGGELE-TTNNRMELMAAIRGLEAL-------TKP------CKVRLTTDSQYVRQGIT-QWLSGWKKKNWMTSSRQ---PVKNKELWQRLDSAVSK--H-DV-EWHWVKGHSGHIENERADDLANKGV-----EQVT------KS-----------------------------

>Proteobacteria gamma | Methylococcus ca | YP_113229

-----------------------------------MSETEPTVYAYTDGACRG-NP-GP----GGWGVLLR---Y-G-S--------KTR-----EIYGGERE-TTNNRMELMAAIRALETL-------SRP------CKVKIVTDSQYVKKGIT-EWVAQWEKRGWKTAGRS---PVKNIDLWQRLIQAEQR--H-QV-SWGWIKGHSGHPENEAADRLANRGI-----DELL------QSDKIPA------------------------

>Proteobacteria alpha | Marinomonas sp. | ZP_01075303

--------------------------VFLKKANRKRDEIVKHVIIYTDGACKG-NP-GP----GGWGAWIT---F-G-E--------HEK-----RLCGGEND-TTNNRMELSGAIEGLKAL-------TEP------CKVTLYTDSSYVQKGIT-QWLAGWKKKGWKTASKQ---PVKNKDLWQALDEECQR--H-DI-EWKWVKGHAGIKGNEIADELANLGI-----EKIR------D------------------------------

>Proteobacteria alpha | Marinomonas sp. | YP_001340565

--------------------------------------------MYTDGACKG-NP-GI----GGWGAWLT---F-G-E--------HEK-----HLCGGEHD-TTNNRMELMGAIEGLRAL-------KEK------CSVTLYTDSSYVQKGIT-EWLAGWKRKGWMTASKQ---PVKNKDLWQALDEQCQY--H-EV-TWKWVKGHAGIEGNEIADQLANKGI-----DELR------AAS----------------------------

>Proteobacteria beta | Dechloromonas ar | YP_284812

------------------------------------MTAEETVEIFTDGACKG-NP-GP----GGWGAILR---L-G-P--------HEK-----ELWGGEKE-TTNNRMELTAAIRAIEAL-------KRP------IGGKIYTDSQYVMKGIN-EWIHGWKKNGWKTSDKK---PVKNADLWQLLDAQVKL--H-KL-EWIWVRGHSGHPENERADALANRGI-----EELK------G------------------------------

>Proteobacteria gamma | Marinobacter sp. | ZP_01738333

--------------------------------------MSGNVIMYTDGACKG-NP-GR----GGWGVVLR---W-G-E--------VCK-----TLHGGEQH-TTNNRMELMAAIEGLKAL-------KRD------CDVELYTDSQYVRKGIT-EWLAGWKRNGWKTAAKK---PVKNDDLWKALDEQSER--H-RV-NWHWVKGHAGVPDNELADQLANQGV-----EELT------G------------------------------

>Proteobacteria gamma | Marinobacter alg | ZP_01895194

--------------------------------------MAGKVVLYTDGACKG-NP-GP----GGWGVVLR---Y-G-D--------ANK-----MLHGGEAN-TTNNRMELMAAIQGLKAL-------RRT------CDVELYTDSQYVRKGIT-EWMTGWKRNGWKTSAKK---PVKNEDLWRELDNEVAR--H-KV-NWHWVKGHSGNPDNELADELANRGV-----EELA------NAG----------------------------

>Proteobacteria gamma | Marinobacter aqu | YP_958805

--------------------------------------MAGKVVMYTDGACKG-NP-GP----GGWGVVLR---Y-G-D--------ACK-----TMHGGELQ-TTNNRMELMAAIRGLREL-------KRA------CQVELYTDSQYVRKGIT-EWMSGWKRNGWKTSAKK---PVKNADLWQELDAETAR--H-TV-NWHWVKGHSGHPDNELADELANRGV-----RELN------GA-----------------------------

>Proteobacteria gamma | Thioalkalivibrio | ZP_03690355

-------------------------------------MTDERVYIYTDGACRG-NP-GP----GGWGAILR---Y-R-G--------TER-----ELYGAEAE-TTNNRMELTAAIRALETL-------KRP------CKVELVTDSKYVKQGLT-EWLPGWKRRNWKSASGS---PVKNRDLWEALDAEAAR--H-DI-EWYWVRGHSGHPENERADELANAGI-----DALL------AGQPIEN------------------------

>Proteobacteria gamma | Alcanivorax bork | YP_692944

---------------------------------------MKNVIIYTDGACRG-NP-GP----GGWGAILL---Y-G-D--------KEK-----ELFGGEPE-TTNNRMELMAAIVALETL-------NTP------CQVVLTTDSKYVMDGIT-QWMANWKKRGWKTASKQ---PVKNVDLWQRLDAAVQR--H-DI-DWQWVKGHSGHPGNERADALANRGI-----DEMK------HKQGQAS------------------------

>Proteobacteria gamma | Kangiella koreen | YP_003147108

---------------------------------------MKKIEIYTDGACKG-NP-GP----GGWGALLR---Y-N-K--------HEK-----HLFGGELN-TTNNRMELMAAIEALKAL-------KDK------CQVDLTTDSVYVKNGIN-QWLENWKAKGWKTANRK---PVKNQDLWQQLDQQVAR--H-NV-TWHWVKGHSGHPENDIADELANKGV-----EKVL------QSSGV--------------------------

>Proteobacteria gamma | Thioalkalivibrio | YP_002513030

------------------------------------MAESQRVEIYTDGACRG-NP-GP----GGWGAVLR---F-K-G--------RER-----TLKGAEAE-TTNNRMELTAAIMALETL-------TRP------CAVDLTTDSQYVKQGLT-QWIHGWKRKGWRTADGK---PVKNQDLWMRLDAAAAR--H-EV-AWHWVRGHTGHPENELADQLANEAI-----DEML------AGA----------------------------

>Proteobacteria alpha | Magnetospirillum | YP_420125

--------------------------------MSETAPKPETVEIYTDGACSG-NP-GP----GGWGAILR---F-K-G--------IEK-----ELKGGESP-TTNNRMEMMAVLVALNTL-------TRS------CAVDVYTDSEYVKKGMT-EWLRGWKARGWKTADKK---PVKNDDLWKALDEAAAR--H-KV-SWHWVKGHAGHPENERADALAREGI-----ADLR------ART----------------------------

>Proteobacteria alpha | Magnetospirillum | CAM75322

--------------------------------------MTERVEIFTDGACSG-NP-GP----GGWGAILR---Y-K-G--------VEK-----ELCGGENP-TTNNRMEMMAAIMALETL-------SRS------CPVTLYTDSQYVMKGMT-EWLKGWKARGWKTADKK---PVKNDDLWQRLDAACAR--H-QI-TWQWVKGHAGHPENERADQLARDGI-----KVVL------GK-----------------------------

>Proteobacteria gamma | HTCC2080 | ZP_01625362

---------------------------------------MKVVEAFTDGACKG-NP-GP----GGWGVLLR---M-D-N--------QSR-----EIFGGDGA-TTNNRMELTAAIEALAAL-------KEA------CTVELTTDSTYVKDGVT-RWMENWERNGWRTAAKK---PVKNQDLWQALKAQVAR--H-EV-NWHWVKGHSGHPENELADMLANKGI-----AELQ------R------------------------------

>Proteobacteria gamma | NOR5-3 | ZP_05126429

---------------------------------------MKNVELFTDGACRG-NP-GP----GGWGALLC---Y-A-G--------KER-----EVYGAEPN-TTNNRMELSAAIEGLAAL-------SEP------CAVRLVTDSTYVMKGIT-EWLPNWKRRGWKTSAKK---PVANADLWQLLEVQNQR--H-KV-SWEWVKGHSGHPGNERADALANRAI-----DEML------S------------------------------

>Proteobacteria gamma | Congregibacter l | ZP_01101009

---------------------------------------MKRVDLFTDGACRG-NP-GP----GGWGALLV---Y-G-S--------KER-----ELYGGAAD-TTNNRMELSAAIEGLAAI-------SEP------CAVKLVTDSTYVMKGIT-EWLPNWKRRGWKTAAKK---PVANADLWQRLEAECER--H-SI-EWEWVKGHSGHPGNERADALANKAI-----DEML------A------------------------------

>Proteobacteria gamma | Thiomicrospira c | YP_391198

---------------------------------------MQEVELFTDGGCRG-NP-GP----GGWGALLR---F-G-G--------VEK-----ELKGAELD-TTNNRMELTAAIEGLKAL-------KRP------CKVTLTTDSQYVKNGIT-QWMTNWKKNNWKTAAKK---PVKNKDLWQALDEALQP--H-DV-TWAWVKGHSGHDENERVDELANQAM-----DELT------G------------------------------

>Proteobacteria gamma | Pseudomonas aeru | NP_250506

------------------------------------MTDKEQVVIYTDGACKG-NP-GR----GGWGALLL---Y-K-G--------AER-----ELWGGEPD-TTNNRMELMAAIQALAAL-------KRS------CPIRLITDSEYVMRGIT-EWLPNWKKRGWKTASKQ---PVKNADLWQALDEQVAR--H-QV-EWQWVRGHTGDPGNERADQLANRGV----AELPR-------------------------------------

>Proteobacteria gamma | Pseudomonas stut | YP_001172753

-------------------------------------MSDDWVEIYTDGACKG-NP-GP----GGWGALLI---Y-K-G--------VKR-----ELWGGEPD-TTNNRMELMAAIRALAEL-------KRP------CKVRLVTDSQYVMQGIN-DWMPNWKKRGWKTASKQ---PVKNADLWQQLDEQVNR--H-EV-SWQWVRGHTGHPGNEQADLLANRGV-------VQ------AKRQPIV------------------------

>Proteobacteria gamma | Azotobacter vine | YP_002800101

--------------------------------------MSDKVEIYTDGACKG-NP-GP----GGWGALLV---C-Q-G--------VER-----ELWGGEAE-TTNNRMELTAAIRALAEL-------KRP------CEVHLTTDSEYVMRGIL-EWLPNWKKRGWKTAARQ---PVKNADLWQQLDEQVGR--H-RV-TWGWVRGHTGHPGNERADLLANRGV-----AAAR-----AQKKHGV-------------------------

>Proteobacteria gamma | Pseudomonas mend | YP_001187559

------------------------------------MSETDEVVIYTDGACKG-NP-GP----GGWGALLV---Y-K-G--------VEK-----ELWGGDPS-TTNNRMELMAAIAGLIAL-------TRP------CSVKLVTDSQYVMKGIQ-EWLPNWKKRGWKTASKE---PVKNADLWQKLDEEVNR--H-QV-SWQWVRGHTGHPGNERADQLANRGV-----DEVR------SAR----------------------------

>Proteobacteria gamma | Pseudomonas ento | YP_609085

--------------------------------------MSDSVEIYTDGACKG-NP-GP----GGWGVLMV---F-K-G--------VEK-----ELWGGERE-TTNNRMELMAAIEGLKAL-------KRE------CEVVLTTDSQYVMKGIN-EWMVNWKKRGWKTAAKE---PVKNADLWMALDEQVNR--H-KV-TWKWVRGHIGHPGNERADQLANRGV-----DEVR------AQR----------------------------

>Proteobacteria gamma | Pseudomonas puti | YP_001750319

--------------------------------------MSDSVELYTDGACKG-NP-GP----GGWGVLLI---Y-K-G--------VEK-----ELWGGERE-TTNNRMELMAAIQGLMAL-------KRE------CEVVLTTDSQYVMKGIN-EWMVNWKKRGWKTAAKE---PVKNADLWQQLDEQVNR--H-KV-TWKWVRGHIGHPGNERADQLANRGV-----DEVR------AKR----------------------------

>Proteobacteria gamma | Pseudomonas syri | ZP_05637838

--------------------------------------MSDSVELFTDGACKG-NP-GP----GGWGALLV---C-K-G--------VEK-----ELWGGEAN-TTNNRMELTGAIRGLEEL-------KRP------CEVTLVTDSQYVMKGIT-EWMVNWKKRGWKTAAKE---PVKNADLWQLLDEQVSR--H-NV-KWQWVRGHIGHPGNERADQLANRGV-----DEVR------GIKS---------------------------

>Proteobacteria gamma | Pseudomonas fluo | YP_002872231

--------------------------------------MTDSVELFTDGACKG-NP-GP----GGWGALLV---C-K-G--------VEK-----ELWGGEAN-TTNNRMELMGAIRGLEEL-------KRR------CNVLLVTDSQYVMKGIN-EWMVNWKKRGWKTAAKE---PVKNADLWQLLDEQCNR--H-DI-TWKWVRGHIGHPGNERADQLANRGV-----DEVR----------GYKQS----------------------

>Proteobacteria gamma | Pseudomonas fluo | YP_347918

-----------------------------------MSESVDSVELFTDGACKG-NP-GP----GGWGALLV---C-K-G--------VEK-----ELWGGEAN-TTNNRMELLGAIRGLEAL-------KRP------CEVLLVTDSQYVMKGIN-EWMANWKKRGWKTAAKE---PVKNADLWKALDEQVNR--H-KV-TWKWVRGHIGHHGNERADQLANRGV-----DEVR----------GYKQS----------------------

>Proteobacteria gamma | Pseudomonas fluo | YP_260402

--------------------------------------MSDSVEIFTDGACKG-NP-GP----GGWGALLV---C-K-G--------VEK-----ELWGGEAN-TTNNRMELMAAIRGLEEL-------KRQ------CDVQLVTDSQYVMKGIN-EWMANWKKRGWKTAAKE---PVKNADLWQQLDEQVNR--H-NV-TWKWVRGHTGHHGNERADQLANRGV-----DEVR----------GYKQP----------------------

>Proteobacteria gamma | HTCC2207 | ZP_01224052

---------------------------------------MKAVELFTDGACRG-NP-GP----GGWGVLMR---Y-G-D--------KEK-----TLCGGEAE-TTNNRMELTAVIEGIAAL-------SEP------CKVSVTSDSTYVLKGIQ-EWMPAWKKRNWKTASKK---PVKNVDLWQKLDAVIKH--H-DI-DWHWVKGHSGHAENEIADQLANRGI-----DEL--------------------------------------

>Proteobacteria gamma | NOR51-B | ZP_04958093

--------------------------------------MTTSVEAFTDGACRG-NP-GP----GGWGVLLR---R-G-D--------RER-----ELWGGEAQ-TTNNRMELTAAIEALRSL-------KDG------STVDLTTDSVYVRDGIT-RWVAGWKRNGWRTAARK---PVKNQDLWQSLDEQCAR--H-EV-RWHWVKGHSGHAENDRADALANRGI-----DELK------G------------------------------

>Proteobacteria gamma | Neptuniibacter c | ZP_01167471

---------------------------------------MKTVEIFTDGACKG-NP-GP----GGWGAVLR---Y-G-D--------AEK-----QMHGGEND-TTNNRMELMAAIVALETL-------NRP------CEVILTTDSQYVRQGIT-EWIEGWKRKGWKNSQKK---PVKNADLWQRLDAARQP--H-KI-DWRWVKGHSGHPENELADQLANKGV-----EELG------RA-----------------------------

>Proteobacteria gamma | Nitrococcus mobi | ZP_01128586

---------------------------------------MAPVEIYTDGACRG-NP-GP----GGWGAVLR---Y-G-G--------HEK-----SLCGGATQ-TTNNRMELTAAIQALESL-------KRP------CRVVLTTDSQYLRRGIT-EWLPNWKRRGWRTAERK---PVKNADLWQRLDMLAAR--H-EV-DWRWVRGHNGHPGNEQADRLANQGI-----DEML------ARR----------------------------

>Proteobacteria gamma | Alkalilimnicola | YP_742824

------------------------------------------MYAWTDGACRG-NP-GP----GGWGVVLR---Y-R-G--------HER-----TLHGGEPH-TTNNRMELTAAIQALEAL-------DRP------CVVHLTTDSQYVRKGIT-EWMAGWKRRGWRTAARK---PVLNEDLWRRLDALNQR--H-EV-HWHWVRGHSGHAENEQADALANRGI-----DEMQ------EAGAT--------------------------

>Proteobacteria gamma | Halorhodospira h | YP_001003151

-----------------------------------MTEQRGVVEAFTDGACRG-NP-GP----GGWGVLLR---Y-G-E--------HER-----ELYGGEPE-TTNNRMELTAAIRALEAL-------DRP------CRVVLTTDSQYVRRGIT-EWLEGWKRRGWRTASRK---PVLNQDLWQRLDELAAY--H-QV-DWHWVRGHAGHAENERADALANQGI-----DELV------A------------------------------

>Proteobacteria gamma | Teredinibacter t | YP_003073577

---------------------------------------MKKIEIFTDGACRG-NP-GP----GGWGVLLR---Y-G-D--------KEK-----TLHGGERD-TTNNRMELRAAIEGLSAL-------KEP------CEVRLVTDSQYVRKGIT-EWIANWKKRGWRTAAKK---PVMNVDLWQALDTACDQ--H-QI-TWEWVKGHSGHRENEIADELANLGI-----DELN------VRR----------------------------

>Proteobacteria gamma | HTCC2148 | ZP_05093760

---------------------------------------MKNIEIFTDGACRG-NP-GP----GGWGALLR---F-Q-G--------KEK-----SLYGGEAQ-TTNNRMELQAAIEGLKAL-------KEP------CVVALTTDSIYVKNGIT-SWLPGWKKKGWKTSNKK---PVKNVDLWQSLDEQNQR--H-QV-DWHWVKGHSGHRENEIADQLANRGI-----DELT-------------------------------------

>Proteobacteria gamma | Cellvibrio japon | YP_001982514

---------------------------------------MKTVEIFTDGACKG-NP-GP----GGWGALLR---Y-G-Q--------VEK-----SLYGGEPE-TTNNRMELMAAIAALSAL-------KEP------CAVVITTDSQYVRKGIT-EWMPGWKRNGWRTAAKE---PVKNADLWQRLDEQNQR--H-QV-TWKWVKGHSGHRENELADALANRGI-----DELR-------------------------------------

>Proteobacteria delta | Stigmatella aura | ZP_01463545

-------------------------------------MTLPLVHIYCDGACSP-NP-GI----GGWGSILVSPAH-G-H--------ARK-----ELSGAEPG-TTNNRMELTAALMALRAL-------KSP------CQVQLFTDSQYVRNAFQEKWLDKWQRTGWKTAGRQ---PVQNADLWQALLEQTRV--H-QV-SWNWVRGHSGHVENERADAMAVAAR-----LA----L---AAKLGR-------------------------

>Proteobacteria gamma | Buchnera aphidic | NP_777852

--------------------------------------MLKTIKIFSDGSCLG-NP-GP----GGYSFIIQ---H-L-E--------YEN-----ISSSGFYL-TTNNRMELMGIIVATESL-------KQP------CCITISTDSQYVQKGIL-YWIKNWKTKGWKTSRKT---YVKNVDLWLRLEKSLNL--H-QV-TWKWIKSHSGNKKNEQCDHLARESA-----KFPT------LKDFGYIL-----------------------

>Proteobacteria delta | Desulfonatronosp | ZP_03736090

---------------------------------MSRNNNTQVVDIYTDGACLG-NP-GP----GGYAAILK---W-G-D--------LEK-----EISQGTPG-TTNNRMELMAVLEGLKAL-------KYP------CRVRIHTDSQYIARAINEKWLEKWQRNGWKTAQKE---DVKNRDLWEELAALLQE--H-KV-EFKWVRGHSGHEYNERCDSLARQAA-----QAVDD------------------------------------

>Proteobacteria delta | Desulfomicrobium | YP_003156805

-------------------------------------MTDTSVTIYTDGSSLG-NP-GP----GGWGAVLI---W-A-D--------SKK-----ELSRGYIE-TTNNRMEIRGVLHALEHL-------KRP------CTVHVHSDSRYVCDAISKKWIQSWLKNGWLTSAKK---PVKNRDLWEQLLSLLQK--H-KV-IFHWVKAHDGHPENERCDELAKNAA-----KARE-----REIDEGYLRNT---------------------

>Proteobacteria gamma | Buchnera aphidic | NP_660587

--------------------------------------MLKLVKMFSDGSCLG-NP-GS----GGYGTILR---Y-K-L--------HEK-----ILTSGFFL-TTNNRMELMGVICGLESL-------KES------CIVEITIDSQYVKQGIT-NWIATWEKKKWKTTKKK---LIKNLDLWLRINAVIKN--H-HI-TWFWVKAHMGHLENERCDKIARQSA-----QSPS------VKDFFYENNFYQNKNL---------------

>Actinobacteria | Atopobium vagina | ZP_03946132

-------------------MENSSSIKAKAMDAARTNSTKPQVIIWTDGSSRG-NP-GP----GGYGAVML---F-Y-D---SAGREHKR-----ELSCGYRQ-TTNNRMELLAPIIALEEL-------KYP------CKVELHSDSQYVIHAFQQHWIDGWQKRGWKTANKQ---PVKNVDLWKRLLRAMQP--H-EM-SFVWVKGHAGTELNERCDELATTAA-----DADV---SLLQVDEGFEALS---------------------

>Actinobacteria | Atopobium rimae | ZP_03568735

----------------------MYEDVYTQSETIGSSSKRAQVVAYTDGASRG-NP-GP----GGYGAVLV---Y-V-D---ASGKRHTR-----EFSQGYRL-TTNNRMELLGVIVALEAL-------TQP------CVVEVHSDSKYVVDAFNQGWIFGWMRRGWKTSNKQ---NVKNIDLWKRLLVASSS--H-EV-HYVWVKGHAGEELNERCDELATTAA-----DGQD-----LLEDVGFQGE----------------------

>Firmicutes | Butyrivibrio cro | ZP_05792240

---------------------------------------MNNVIIYTDGAARG-NPNGP----GGYGVVLE---Y-T-D---KNGIVHHK-----ELSQGYKK-TTNNRMELMAAIAGLEAL-------KAP------CNVTLYSDSKYLVDAFRQKWIDSWIAKDFKRGKNE---PVKNPDLWKRLLKAKEN--H-NV-EFVWVKGHAGHAMNEKCDMLATSAA-----DGDN--L---ADDVTLENIV---------------------

>Actinobacteria | Atopobium parvul | YP_003180122

---------------------------------MEAATSYMHVTVYTDGASRG-NP-GP----GGYGAVLL---Y-T-D---PSGQQHTK-----EFSQGYKT-TTNNRMELLGVIVALEAL-------KRP------CQVELYSDSKYVVDAFNQKWVSGWVRKGWKTASKE---PVKNVDLWKRLLAAMED--H-EV-SFKWVKGHAGHPLNERCDQLATEAA-----DGSN-----LLDDQGFFADQSLL------------------

>Firmicutes | Clostridium hath | ZP_06117226

---------------------------------------MGKVLLFTDGAARG-NPDGP----GGYGAVLQ---F-T-D---SKGQLHEK-----TLSAGYVR-TTNNRMELMAAIAGLEAL-------NRP------CEVELYSDSKYVTDAFNQHWIDNWVKNNWKRGKSG---PVKNIDLWKRLLKAMEP--H-RV-TFCWVKGHAGHPENERCDQLATTAA-----DGDS-----LLVDEGL-------------------------

>Firmicutes | Clostridium phyt | YP_001559482

----------------------------------------MKVTIYTDGAARG-NPDGP----GGYGTILS---Y-I-D---STGVEHIR-----EYSGGYKK-TTNNRMELMAAIVGLEAL-------TKP------CVVTLYSDSQYVVKAFNEHWLDGWIKKGWKRGKNE---PVKNVDLWKRLLAAKNQ--H-DV-TFCWVKGHDGHPQNERCDVLATTAA-----DGGN------LADDNVVE-----------------------

>Firmicutes | Bryantella forma | ZP_05348692

----------------------------------------MLVKIYTDGAARG-NPDGP----GGYGTILH---Y-T-D---TKGVLHER-----TFSQGYEK-TTNNRMELMAAIIGLEAL-------NRP------CQVELYSDSKYLTDAFNRHWIDSWQRKGWKRGKNE---PVKNVELWKRLLAAMEP--H-EV-SFIWVKGHDGHELNERCDRLATSAA-----DGEE----LAVDDGGEMR-----------------------

>Actinobacteria | Eggerthella lent | YP_003181454

----------------------------------------MHVDIYTDGAARG-NP-GP----GGYGTVLR---F-V-D---SKGAVHEK-----ELSQGYER-TTNNRMELMAVVAGLEVL-------KRP------CSITLYSDSQYVVNAFNQHWVDGWLKRGWKNAQKQ---PVKNDDLWKRLLAAKEP--H-DV-TFVWVKGHAGHPENERCDELATTAA-----DGAG-----RIRDEGFNG-----------------------

>Actinobacteria | Slackia heliotri | YP_003143435

----------------------------------------MHVEIYSDGSSRG-NP-GP----GGYGSVLH---Y-T-D---AQGQLHVK-----ELSQGFVT-TTNNRMELLGVIVALEAL-------KRP------CSVDVYSDSQYVVKAFNDHWIDGWLKRGWKNSKKE---RVKNQDLWRRLLAAKAP--H-QV-SFHWVKGHAGHPENERCDQLATEAA-----DGSG-----LILDEGFTSEDV--------------------

>Actinobacteria | Slackia exigua A | ZP_06159453

----------------------------------------MHVDIYSDGSSRG-NP-GP----GGYGAILR---F-V-D---PSGGVHQK-----ELSGGFER-TTNNRMELLGVIVALEAL-------KAP------CEVAVYTDSQYVVKAFTDRWVDGWKRRGWKNAKKE---PVKNQDLWMRLIAALEG--H-RV-SFHWVKGHAGHPENERCDELATTAA-----DGAD-----RLIDEGFTD-----------------------

>Nitrospirae | Leptospirillum f | EES51503

-------------------------------------MESDEVELFADGACSG-NP-GP----GGWGVLLR---C-R-G--------HVR-----EISGGEFQ-TTNNRMELSGVIAGLSAL-------KKP------CRVMVTTDSQYVKNGMT-TWIRSWKKNGFRTSSGQ---PVKNEDLWRELDRLAAL--H-EI-TWHWVRGHDGHPENERVDLLAREAI-----SSVR--K---GNSQGA-------------------------

>Proteobacteria delta | Desulfuromonas a | ZP_01311301

------------------------------------MSQKQHVEIYSDGACRG-NP-GP----GGYGTLLR---C-G-S--------HIK-----ELSGYEAQ-TTNNRMELLGAIAGLEAL-------KKP------CIVTLTTDSQYVYKGMT-QWLSGWKKKGWKNSQKK---DVLNRDLWERLERAAQD--H-EV-TWQWVKGHAGHEENERCDELARTAI-----DLAE-------------------------------------

>Proteobacteria delta | Pelobacter carbi | YP_355635

-----------------------------------MSESRSMVEIFSDGACSG-NP-GP----GGFGTLLR---C-G-E--------RVR-----ELSGFDPE-TTNNRMELLGAIAGLEAL-------TRP------CRVRLTTDSQYVCKGMT-EWIHGWQKKGWKNSKKE---DVANRDLWERLLVLVSK--H-EV-SWHWVRGHAGHAENERCDELARQAI-----ADGC--S---SVV----------------------------

>Proteobacteria delta | Pelobacter propi | YP_902594

-----------------------MSARPDQSRTAAPSATTSPVEIYCDGACSG-NP-GP----GGYGAILR---Y-N-G--------HEK-----EIRGSEAH-TTNNRMELTAAMEALRLL-------TRP------CRITIVTDSQYLVKGMT-EWIQGWQRRGWQNSKKE---PVLNRDLWEELLKLSAH--H-DV-SWQWIRGHAGHAENERCDSLARQAI-----TEMR------GAP----------------------------

>Proteobacteria delta | Geobacter lovley | YP_001953011

---------------------------------MKQQNSLTEVEIFCDGACSG-NP-GP----GGYGTILR---C-R-G--------KEK-----ELSGAATE-TTNNRMELTAALEGLRQL-------TRS------CRVTITTDSQYLVKGMT-EWLPGWQRNGWKNSKKE---PVLNRDLWEALVEASKP--H-QV-AWQWVRGHAGHAENERCDTLAREAI-----SAMQ------VRN----------------------------

>Proteobacteria delta | Geobacter sulfur | NP_953120

--------------------------------------MSAEVEVFCDGACSG-NP-GV----GGYGAILR---Y-G-S--------AEK-----ELSGADGD-TTNNRMELTAAIRALEAL-------SRP------CAVTITTDSQYLVKGMT-EWLSGWVRRGWVNSKKE---PVLNRDLWERLRELTGK--H-QV-RWVWVRGHNGHPENERCDALARRAI-----DAYR------NERR---------------------------

>Proteobacteria delta | Geobacter sp. FR | YP_002538914

----------------------------------------MKVEIFCDGACSG-NP-GV----GGWGCILR---Y-G-D--------NVK-----EMSGADGN-TTNNRMEMTAAIEALASL-------KRP------CEVHLTTDSQYLVKGMT-EWIGGWVRKGWVNSKKE---PVLNRELWERLMELSRL--H-TI-HWLWVRGHNGHPENERCDELARAAI-----ETFR------RSC----------------------------

>Proteobacteria delta | Geobacter uranii | YP_001231597

----------------------------------------MKVEIFCDGACSG-NP-GV----GGWGSILR---Y-G-D--------TVK-----ELSGADGD-TTNNRMEMTAAIEALASL-------KRP------CEVVLTTDSQYLVKGMT-EWMSGWIRKGWVNSKKE---PVLNRELWERLLALSKI--H-KI-RWAWVRGHNGHPENERCDELARAAI-----EVFK------GRKP---------------------------

>Proteobacteria delta | Geobacter sp. M1 | ZP_05310359

----------------------------------------MQVEIFCDGACSG-NP-GV----GGWGSILR---Y-G-D--------KVK-----ELSGAEGE-TTNNRMEMSAAIGALEAL-------TRP------CEVVVTTDSQYLAKGMT-EWVAGWIRKGWVNSKKE---PVVNRDLWERLVALARV--H-RI-KWVWVRGHNGHVENERCDELARAAI-----DSYRAARRQETVSPPNPPAADTRL-----------------

>Proteobacteria delta | Geobacter sp. M2 | YP_003021576

----------------------------------------MQVEIFCDGACSG-NP-GV----GGYGSILR---C-G-E--------TVK-----EISGADGD-TTNNRMEMSAAIAALEAL-------KRP------CQVVVTTDSQYLAKGMT-EWLSGWVKRGWVNSKKE---PVLNRDLWERLLELSKV--H-QI-RWVWVRGHNGHVENERCDELARAAI-----DSYR------AGNGR--------------------------

>Proteobacteria delta | Desulfovibrio sa | YP_002989784

-------------------------------------MSKKKLTIYTDGSCLG-NP-GK----GGYGAVLL---F-N-E--------HRK-----ELSQGYKK-TTNNRMEMRAVIAALTEL-------KEP------CEVTLYTDSQYVKNAFTKKWIDNWQKNGWKTAAKK---PVKNKDLWLQFIPLLEK--H-DV-TFRWVKGHAGDPENERCDDLARTAA-----SSGD-----LIVDEGA-------------------------

>Proteobacteria gamma | Baumannia cicade | YP_588912

--------------------------------------MRKKIEIFTDGSCFG-NP-GP----GGYGAILR---Y-K-K--------YEK-----EHSAGFLL-TTNNRMELMAAIIALEFL-------RDP------CEAIVYIDSKYVHQGVI-QWIYNWKKHNWKNSAKK---IIKNLDLWQRLDVVSNL--H-VI-HWRWVKSHTGHPENERCDELARIAA-----EHPK------FEDIGYKR-----------------------

>Proteobacteria gamma | Pseudoalteromona | ZP_01132449

--------------------------------------MRKSVAIYTDGSCLG-NP-GP----GGYGVVMR---Y-N-E--------HLK-----ELSQGFEL-TTNNRMELLAAIVGLESL-------KQA------CDVVLTTDSQYVKQGIE-SWLQGWKKRNWLTANKQ---PVKNIDLWQRLDIINQT--H-HV-QWRWVKGHSGHFENERCDVLARSAA-----ESST-----LLPDEGYRATE---------------------

>Proteobacteria gamma | Haemophilus para | ZP_02478158

---------------------------------------MKLVDIFTDGSCLG-NP-GK----GGIGILLR---Y-Q-G--------KEK-----RISQGYYL-TTNNRMELLAVITALNAL-------KEP------CNVHLHSDSQYMQNGIQ-KWIFNWKKNNWKTSNNT---PVKNQDLWIALDKAITR--H-QV-EWQWVKGHSGHTENEICDQLAKEGA-----NNPT------LEDVGYPPS----------------------

>Proteobacteria gamma | Haemophilus ducr | NP_873664

---------------------------------------MKSVNIFTDGSCLG-NP-GP----GGIGVVLR---Y-N-Q--------HQK-----KVSQGYFQ-TTNNRMELRAVIEGLSML-------KEA------CNVTLYSDSQYMKNGIT-KWIFKWKKSNWKTANGK---AVKNKDLWLLLDEKIQI--H-YI-EWKWVKGHSGHYENEICDELAKLGA-----NNPT------LEDVGYQPA----------------------

>Proteobacteria gamma | Mannheimia haemo | ZP_04977670

--------------------------------------MMKLVEIFTDGSCLG-NP-GK----GGIGILLR---Y-N-G--------YEK-----TVSKGYFQ-TTNNRMELRAVIEALAML-------KEP------CKVQLNSDSQYMKNGIQ-KWIFNWKKNDWKTSDKK---PVKNKDLWVALDQEIQR--H-QI-EWSWVKGHSGHRENEICDELAKQGA-----NNPT------LDDVGYIAD----------------------

>Proteobacteria gamma | Actinobacillus m | ZP_04752263

---------------------------------------MKQVEIFTDGSCLG-NP-GK----GGIGILLR---Y-N-Q--------HEK-----TVSQGYFQ-TTNNRMELRAVIEALAML-------KEP------CQVHLHSDSQYMKDGIT-KWIFNWKRNNWKTANGK---AVKNQDLWIALDMEIQR--H-KM-EWHWVKGHAGHRENEICDELAKAGA-----NNPT------LEDIGYNAE----------------------

>Proteobacteria gamma | Actinobacillus p | YP_001651475

---------------------------------------MKLVEIFTDGSCLG-NP-GK----GGIGIVLR---Y-N-G--------HEK-----QVSKGYLQ-TTNNRMELRAVIEALAML-------KEP------CQVQLNSDSQYMKDGIT-KWIFNWKKNNWKTANGK---PVKNKELWIALDQEIQR--H-KI-EWTWVKGHSGHRENEICDELAKAGA-----NNPT------LEDIGYNAD----------------------

>Proteobacteria gamma | Haemophilus somn | YP_718816

--------------------------------------MLKKIEIFTDGSCLG-NP-GA----GGIGILLR---Y-K-Q--------HEK-----KLYQGFFQ-TTNNRMELRAVIVALNSL-------KEP------CSVILYSDSQYMKNGIT-KWIFNWKKNNWKTSSGN---AVKNQDLWCSLDQAIQK--H-QI-EWRWVKGHNGHRENEICDQLAKQGA-----ENPT------LEDVGYRAE----------------------

>Proteobacteria gamma | Mannheimia succi | YP_088763

----------------------------------MYQIMRKQIEIFTDGSCLG-NP-GA----GGIGVVLR---Y-K-Q--------HEK-----TLSQGYFK-TTNNRMELRAVIEALNLL-------KEP------CAVTLHSDSQYMKNGIT-QWIFNWKKKNWKASNGK---PVKNQDLWMALDNAVQA--H-TI-DWRWVKGHSGHRENELCDQLAKQGA-----ENPT------LEDIGYQPD----------------------

>Proteobacteria gamma | Actinobacillus s | YP_001344181

--------------------------------------MRKQIEIFTDGSCLG-NP-GV----GGIGVVLR---Y-K-Q--------HEK-----TLSKGYFQ-TTNNRMELRAVIEALNLL-------KEP------CEIILHSDSQYMKNGIT-QWIFNWKKNNWRASTGK---PVKNQDLWIALDSAIQP--H-TI-HWRWVKGHSGHRENEMCDELAKQGA-----ENPT------LEDTGYRQD----------------------

>Proteobacteria gamma | Aggregatibacter | YP_003256376

--------------------------------------MRKQIEIFTDGSCLG-NP-GA----GGIGILLR---Y-K-Q--------HEK-----KLSKGFFL-TTNNRMELLAVVEALNSL-------KEP------CDIHLYSDSQYMKNGIT-QWIFNWKKNHWKASSGK---PVKNQDLWMALDQAIAR--H-KV-DWRWVKGHAGHRENEICDQLAKQGA-----ENPT------LNDEGYQAEA---------------------

>Proteobacteria gamma | Aggregatibacter | YP_003006768

--------------------------------------MRKQIEIFTDGSCLG-NP-GA----GGIGVLLR---Y-K-Q--------HEK-----SLSKGYFL-TTNNRMELLAVIEALNSL-------KEP------CDIHLYSDSQYMKNGIT-QWIFNWKKNNWKASSGK---PVKNQDLWIALDQAIAR--H-KV-DWRWVKGHTGHRENEICDQLAKQGA-----ENPT------LHDEGYQGE----------------------

>Proteobacteria gamma | Haemophilus infl | ZP_05851053

------------------MFNLSLSIKIPAILHNNLFVMQKQIEIFTDGSCLG-NP-GA----GGIGAVLR---Y-K-Q--------HEK-----MLSKGYFK-TTNNRMELRAVIEALNTL-------KEP------CLITLYSDSQYMKNGIT-KWIFNWKKNNWKASSGK---PVKNQDLWIALDESIQR--H-KI-NWQWVKGHAGHRENEICDELAKKGA-----ENPT------LEDMGYFEE----------------------

>Proteobacteria gamma | Haemophilus infl | ZP_04465570

--------------------------------------MQKQIEIFTDGSCLG-NP-GA----GGIGAVLR---Y-K-Q--------HEK-----TLSKGYFK-TTNNRMELRAVIEALNTL-------KEP------CLITLYSDSQYMKNGIT-KWIFNWKKNNWKASSGK---PVKNQDLWKALDESIQR--H-KI-NWQWVKGHAGHRENEICDELAKKGA-----ENPT------LEDMGYIKE----------------------

>Proteobacteria gamma | Pasteurella dagm | ZP_05920208

--------------------------------------MQKQIEIFTDGSCLG-NP-GP----GGIGILLR---Y-K-Q--------HEK-----QISKGYIQ-TTNNRMELRAVVEALNAL-------KEP------CTVTLHSDSQYMKNGIT-KWIFNWKKNNWKASTGK---PVKNQDLWIALDQAIQR--H-NI-NWQWVKGHSGHVENEICDELAKAGA-----EKPT------LEDVGYQPE----------------------

>Proteobacteria gamma | Pasteurella mult | NP_245044

--------------------------------------MQKQIEIFTDGSCLG-NP-GP----GGIGVLLR---Y-K-Q--------HEK-----QISAGYFL-TTNNRMELRAVIEALNTL-------KEP------CSVTLHSDSQYMKNGIT-KWIFNWKKNNWKASTGK---PVKNQDLWIQLDQAIQR--H-HI-NWQWVKGHSGHIENEICDQLAKAGA-----ENPT------LQDVGYQPE----------------------

>Proteobacteria gamma | Idiomarina loihi | YP_156076

------------------------------------MSNSKTVHLYTDGSCLG-NP-GP----GGYGAVLE---Y-G-K--------HHK-----ELSQGYRL-TTNNRMEMLATIAGLREL-------KRS------CHVILTTDSQYVKQGVE-QWMHRWKQNGWRTSARK---AVKNKDLWQQLDEEVNR--H-KV-EWKWIKGHSGHKQNERCDELARDAA----TREPM------LEDEGFGGE----------------------

>Proteobacteria gamma | Idiomarina balti | ZP_01044146

---------------------------------------MAKVHIFTDGSCLG-NP-GP----GGYGVVLE---Y-G-Q--------HHK-----ELSGGYQC-TTNNRMELLACIKGLQVL-------NRA------CDVILTTDSQYVKQGIE-QWIHNWKRNGWRTSNKK---AVKNVDLWQQLDQAIAA--H-KV-TWEWVKGHAGHPQNERCDELARAAA-----EQNP-----TQVDTGFSTE----------------------

>Tenericutes | Candidatus Bloch | YP_277736

--------------------------------------MYKKIEIFTDGSCLG-NP-GP----GGCAAILR---Y-K-Q--------HKK-----EFSIGYRL-TTNNRMELMAAIIALESL-------KNP------CQIILNTDSQYLLHGIT-QWIHIWKKHHWKTSEEK---LVKNIDLWQRLDVAIQI--H-SIIHWNWLKSHTGHPDNERCDQLARLAAKCPINEDFY-------------------------------------

>Tenericutes | Candidatus Bloch | NP_878522

--------------------------------------MYKQIEIFTDGSCLG-NP-GP----GGCAGILR---Y-R-Q--------YKK-----EFSAGYHI-TTNNRMELMAAIIALESL-------KNS------CQIILYSDSQYLLTGIT-QWIQIWKKHHWKTADSK---LVKNIDLWRRLDIAIQP--H-NIKDWRWLKSHTGHPDNERCDQLARKAA-----KYPLNKDFDNNPVVLYNDNDLMKID----------------

>Proteobacteria gamma | Tolumonas auensi | YP_002892125

--------------------------------------MLKQITLYTDGSCLG-NP-GP----GGYAAVLI---Y-K-Q--------HRK-----ELAQGYEL-TTNNRMELMAAIAGLQSL-------SEP------CQVRLTTDSQYVRQGIT-QWIHGWKKKGWKTANRE---PVKNVDLWLLLDSEIQR--H-DV-EWFWVKGHSGHPENERCDELARNAA----LADSR------LIDSGYPS-----------------------

>Proteobacteria gamma | Aeromonas hydrop | YP_856105

--------------------------------------MLKKIDLYTDGSCLG-NP-GP----GGYGAVMV---Y-G-K--------HRK-----ELAGGFRL-TTNNRMELMAAIMGLRTL-------NEP------CQVRLTTDSQYVRQGIT-QWIIGWKKKGWVTASRQ---PVKNVDLWQALDAEVAR--H-QI-EWLWVKGHSGHPENERCDELAREAA-----SGKQ-----LAEDTGYQP-----------------------

>Proteobacteria gamma | Aeromonas salmon | YP_001142554

--------------------------------------MLKHIDLYTDGSCLG-NP-GP----GGYGAVLV---Y-G-D--------HRK-----EISGGFRL-TTNNRMELMAAIMGLRTL-------NAA------CQVRLTTDSQYVRQGIT-QWIIGWKKKGWMTSNRQ---PVKNVDLWKELDAEVAR--H-QI-EWLWVKGHSGHPENERCDELARDAA-----SGKE-----LAEDTGYQP-----------------------

>Proteobacteria gamma | Alteromonadales | ZP_01614115

--------------------------------------MQKTVEIYTDGSCLG-NP-GP----GGYGIFMI---Y-D-A--------HEK-----KLSQGYKL-TTNNRMEMLAAIVALESL-------NRA------CVVNLTTDSQYVKQGIE-SWISNWKKRGWITSAKK---PVKNVDLWKRLDAACSK--H-TV-NWKWVKGHSGHKYNEIVDDLARDAA-----GSTD-----LLEDVGYQP-----------------------

>Proteobacteria gamma | Pseudoalteromona | YP_340464

--------------------------------------MEKTVEIYTDGSCLG-NP-GP----GGYGIFMI---Y-N-E--------HEK-----KLSQGYKL-TTNNRMEMLGAIVALEVL-------TRP------CVINITTDSQYVKQGIE-SWITNWKKRGWLTSAKK---PVKNVDLWKRLDLACAK--H-TV-TWKWVKGHSGHKYNEIVDDLARDAA-----GSKD-----LLDDVGYQP-----------------------

>Proteobacteria gamma | Shewanella denit | YP_563025

-----------------------------------MMTTHKQVNIYTDGSCLG-NP-GP----GGYGIVMQ---Y-K-Q--------HSK-----EIADGFAL-TTNNRMELLAPIIALEAL-------MEP------CIVTLTSDSQYMRQGIT-QWIHGWKKKGWMTSNKQ---AVKNVDLWKRLDSVSQR--H-NI-DWRWVKGHTGHKQNERCDKLARDAA----EAKPK------QIDTGYQESL---------------------

>Proteobacteria gamma | Shewanella oneid | NP_718146

------------------------------------MTELKLIHIFTDGSCLG-NP-GP----GGYGIVMN---Y-K-G--------HTK-----EMSDGFSL-TTNNRMELLAPIVALEAL-------KEP------CKIILTSDSQYMRQGIM-TWIHGWKKKGWMTSNRT---PVKNVDLWKRLDKAAQL--H-QI-DWRWVKGHAGHAENERCDQLARAAA----EANPT------QIDTGYQAES---------------------

>Proteobacteria gamma | Shewanella sp. W | YP_963624

------------------------------------MTERKLIHIFTDGSCLG-NP-GP----GGYGIVMN---Y-K-G--------HTK-----EMSDGFAL-TTNNRMELLAPIIALESL-------KEP------CRVVLTSDSQYMRQGIM-TWIHGWKKKGWMTSNRT---PVKNVDLWKRLDKVSQM--H-TI-DWQWVKGHAGHAENERCDILARSAA----EANPT------QIDEGYQP-----------------------

>Proteobacteria gamma | Shewanella balti | YP_001050372

------------------------------------MTELKLIHIFTDGSCLG-NP-GP----GGYGIVMN---Y-K-G--------HTK-----EMSDGFAL-TTNNRMELLAPIIALESL-------KEP------CQVVLTSDSQYMRQGIM-TWIHGWKKKGWMTSNRT---PVKNVDLWKRLDKASQM--H-TI-DWQWVKGHAGHAENERCDVLARTAA----ESKPT------QPDLGYQP-----------------------

>Proteobacteria gamma | Shewanella halif | YP_001674098

------------------------------------MTGLKQISIYTDGSCLG-NP-GP----GGYGIVLK---Y-K-K--------QTK-----ELADGFAL-TTNNRMELLAPIVALEVL-------KVP------CQVILTSDSQYMRQGIT-QWIHGWKRKGWLTSAGQ---PVKNVDLWKRLDTVSQR--H-QI-DWRWVKGHAGHTENERCDDLARQAA----EAKPS------QEDSGYINQQAQA------------------

>Proteobacteria gamma | Shewanella peale | YP_001502258

------------MNLYISQLNFLGILARNYSYGSNLMTGLKQISIYTDGSCLG-NP-GP----GGYGIVLK---Y-K-K--------RTK-----ELADGFAL-TTNNRMEMLAPIIALEAL-------KVP------CEVILTSDSQYMRQGIT-QWIHGWKRKGWMTSTNQ---PVKNVDLWKRLDTVSQR--H-QV-EWRWVKGHAGHSENERCDDLARQAA----EAKPT------QEDNGYLAQQKQD------------------

>Proteobacteria gamma | Shewanella amazo | YP_927756

------------------------------------MSELKQIRIYTDGSCLG-NP-GP----GGYGVVMI---Y-K-Q--------HRK-----ELADGFAL-TTNNRMELLAPIVALESL-------KEP------CDVILTSDSQYMRQGIT-EWIHGWKKKGWVTASKT---PVKNVDLWQRLDAAAAK--H-KV-DWRWVKGHAGHAENERCDTLAREAA----EAKPT------QIDKGYQP-----------------------

>Proteobacteria gamma | Shewanella frigi | YP_750889

------------------------------------MAELKQLYIFTDGSCLG-NP-GP----GGYGVVMK---Y-K-H--------QQH-----EIADGFSL-TTNNRMELLAPIIALETL-------YEP------CNIILTSDSQYMRQGIM-TWIHGWKKKGWITSTKQ---PVKNVDLWKRLDAVSQL--H-KI-DWHWVKGHAGHIENERCDVLARKAA----EAKPQ------QVDTGYNPE----------------------

>Proteobacteria gamma | Shewanella loihi | YP_001094237

------------------------------------MHGLKQLLIFTDGSCLG-NP-GP----GGYGVVMK---Y-K-A--------HVK-----ELSGGFAL-TTNNRMELLAPIMALEAL-------KEP------CQIILTSDSQYMRQGIT-QWIHGWKKRGWLTAAKE---PVKNVDLWQRLDAATST--H-KI-DWRWVKGHAGHIENERCDTLAREAA----EAGPS------EVDTGYQAKG---------------------

>Proteobacteria gamma | Shewanella benth | ZP_02159445

--------------------------------------MLKPLSIFTDGSCLG-NP-GP----GGYGVVMQ---Y-K-S--------RIK-----ELSDGFLL-TTNNRMELLAPIIALEAL-------KVP------CKIVLTSDSQYMRQGIT-QWIHAWKKKGWQTAAKQ---PVKNVDLWKRLDAATAS--H-EI-EWRWVKGHAGHVENERCDTLARVAA----EAKPT------QEDIGYPV-----------------------

>Proteobacteria gamma | Shewanella sedim | YP_001473729

------------------------------------MMGMKQLSIFTDGSCLG-NP-GP----GGYGVVMK---Y-K-Q--------HTK-----EIADGFLL-TTNNRMELLAPIIALEAL-------KVP------CKIVLTSDSQYMRQGIT-QWIHGWKKKGWITSSKQ---PVKNVDLWKRLDLASKG--H-EI-DWRWVKGHAGHVENERCDTLAREAA----EAKPK------QEDIGYQA-----------------------

>Proteobacteria gamma | Shewanella woody | YP_001760980

------------------------------------MSVLKQLSIFTDGSCLG-NP-GP----GGYGVVMK---Y-K-A--------HTK-----ELSDGFAL-TTNNRMELLAPIIALEAL-------KVP------CKIILTSDSQYMRQGIT-QWIHGWKKKNWITSTKQ---PVKNVDLWKRLDAATQS--H-EI-DWHWVKGHAGHVENERCDTLARVAA----EAKPT------QEDLGYQPSVSSS------------------

>Proteobacteria gamma | Psychromonas ing | YP_941950

-------------------MQVLHNEVGGFIIINQFGIRMKKIQLFTDGSCLG-NP-GP----GGYGAVMI---Y-N-E--------HCK-----ELSEGFLL-TTNNRMEMLACIKALQSL-------TEP------CEVELTTDSQYVRQGIT-LWIHNWKKRGWKTAAKA---PVKNVDLWKALDAAQEK--H-KV-AWHWVKGHSGHPENERCDDLARRAA----ENNPT------QEDIGYEG-----------------------

>Tenericutes | Candidatus Hamil | YP_002923568

---------------------------------------MKQVEIFTDGSCLG-NP-GA----GGYASILR---Y-Q-Q--------HEK-----IFSQGYRL-TTNNRMELMASIVALQAL-------KSP------CTVTLFTDSQYVRQGIT-QWVVHWKKRGWKTSERK---EVKNIDLWKALDAEIQK--H-QI-NWQWVKGHAGHPENERCDKLARLAA-----SSPT------QEDRGYQGSC---------------------

>Proteobacteria gamma | Edwardsiella tar | YP_003296868

--------------------------------------MLKQVEIFTDGSCLG-NP-GP----GGYGAILR---Y-R-Q--------HEK-----ALSAGYRL-TTNNRMELMAAIVALETL-------TSA------CQVTLFSDSQYVRQGIT-QWIHGWKRRGWKTADKK---PVKNVDLWQRLDQAIGP--H-QV-EWIWIKGHAGHPENERCDELARSAA-----GAPS------LEDSGYNPE----------------------

>Proteobacteria gamma | Sodalis glossini | YP_454271

--------------------------------------MRKQVAIFTDGSCLG-NP-GP----GGYGAILR---Y-K-Q--------HEK-----TFSAGYRL-TTNNRMELMAAIVALEAL-------TDA------CEVVLSTDSQYVRQGIT-QWIHNWKKRGWKTAYKK---PVKNVDLWQRLDAAIQP--H-TL-RWDWVKGHSGHPENERCDELARTAA-----CHPA------LEDIGYRVEAQTSGGRAD-------------

>Proteobacteria gamma | Erwinia pyrifoli | YP_002649744

--------------------------------------MLKQVEIFTDGSCLG-NP-GP----GGYGAIMR---Y-G-K--------HEK-----IFSAGFHL-TTNNRMEMMAAIVALEAL-------TQP------CAVVLSTDSQYVRQGIT-SWIHNWKKRGWKTADKK---PVKNVDLWKRLDAALSH--H-DI-NWKWVKGHAGHVENERCDVLARTAA-----GCPT------FDDVGYQA-----------------------

>Proteobacteria gamma | Yersinia ruckeri | ZP_04616430

--------------------------------------MTKQVEIFTDGSCLG-NP-GP----GGYGAILR---Y-K-Q--------HEK-----TFSAGYRL-TTNNRMEMMAAIVALEAL-------TSP------CEITLSTDSQYVRQGIT-QWIHNWKKRGWKTSDRK---PVRNVDLWQRLDAAILG--H-NV-QWEWVKGHAGHPENERCDVLARDAA-----NAPT------LEDTGYNPD----------------------

>Proteobacteria gamma | Yersinia frederi | ZP_04633911

---------------------------------MSLPEMTKQVEIFTDGSCLG-NP-GP----GGYGAILR---Y-K-Q--------HEK-----TFSAGYFL-TTNNRMELMAAIVALEAL-------TSP------CKVTLSTDSQYVRQGIT-QWIHNWKKRGWKTTDRK---PVRNVDLWQRLDLAIQT--H-TV-QWEWVKGHAGHPENERCDELARQGA-----NSPT------LEDSGYNPD----------------------

>Proteobacteria gamma | Yersinia aldovae | ZP_04620327

--------------------------------------MTKQVEIFTDGSCLG-NP-GP----GGYGAILR---Y-K-Q--------HEK-----MFSAGYYL-TTNNRMELMAAIVALEAL-------TSP------CEVTISTDSQYVRQGIT-QWIHNWKKRGWKTTDRK---PVRNMDLWQRLDLAIQT--H-TI-QWEWVKGHAGHPENERCDELARLAA-----NSPT------QDDVGYNPD----------------------

>Proteobacteria gamma | Serratia proteam | YP_001477145

--------------------------------------MLKQVEIFTDGSCLG-NP-GP----GGYGAILR---Y-K-Q--------TEK-----TFSAGFRL-TTNNRMEMMAAIVALEAL-------TTP------CEVTLSTDSQYVRQGIT-TWIHNWKKRGWKTADKK---PVKNVDLWQRLDLAIQR--H-TV-KWEWVKGHAGHPENERCDVLARDAA-----SNPT------QDDVGYKPES---------------------

>Proteobacteria gamma | Serratia odorife | EFA15383

----------------MLTALVYVRLGFYKTDRKSLPEMLKQVEIFTDGSCLG-NP-GP----GGYGAILR---Y-K-Q--------VEK-----TFSAGYRL-TTNNRMELMAAIVALEAL-------TAP------CEVTLSTDSQYVRQGIT-SWIHNWKKRGWKTADKK---PVKNVDLWQRLDLAIQT--H-TI-KWEWVKGHAGHPENERCDVLARDAA-----GNPT------QDDVGYKPEN---------------------

>Proteobacteria gamma | Pantoea sp. At-9 | ZP_05732207

--------------------------------------MRKQVEIFTDGSCLG-NP-GP----GGYGAILR---Y-R-Q--------HEK-----TFSAGYRL-TTNNRMELMAAIVALEAL-------TQP------CEVVISTDSQYVRQGIT-SWIHNWKKRGWKTADKK---PVKNVDLWQRLDLALSS--H-QI-VWEWVKGHAGHPENERCDELARSAA-----SQPT------QDDVGYQPES---------------------

>Proteobacteria gamma | Cronobacter turi | YP_003209201

MWFIPVVSVSRTICRVIAVLIALVYVRLGFLLTGSLPEMRKQVEIFTDGSCLG-NP-GP----GGYGAILR---Y-K-Q--------HER-----TFSAGYRL-TTNNRMELMAAIVSLEAL-------REH------CIVTLSTDSQYVRQGIT-QWIHNWKKRGWKTAEKK---PVKNVDLWQRLDAALSQ--H-EI-KWEWVKGHAGHPENERCDELARAAA-----MAPT------LEDTGYQPEATAS------------------

>Proteobacteria gamma | Enterobacter sp. | YP_001175485

--------------------------------------MTKQVEIFTDGSCLG-NP-GP----GGYGAILR---Y-R-G--------HEK-----TFNEGYHL-TTNNRMELMAAIVALEAL-------KED------CDVVISTDSQYVRQGIT-QWIHNWKKRGWKTADKK---PVKNVDLWKRLDAALSH--H-TI-KWEWVKGHAGHPENERCDELARAAA-----MNPI------QEDVGYQPGS---------------------

>Proteobacteria gamma | Klebsiella pneum | YP_001333913

--------------------------------------MLKQVEIFTDGSCLG-NP-GP----GGYGAIMR---Y-R-Q--------HEK-----TFSAGYRL-TTNNRMELMAAIVALEAL-------KEH------CEVVLSTDSQYVRQGIT-QWIHNWKKRGWKTAEKK---PVKNVDLWQRLDAALGQ--H-KI-KWEWVKGHAGHPENERCDELARAAA-----SHPT------LDDVGYLPES---------------------

>Proteobacteria gamma | Citrobacter sp. | ZP_04560679

--------------------------------------MLKQVEIFTDGSCLG-NP-GP----GGYGAILR---Y-R-G--------REK-----TFSEGYNL-TTNNRMELMAAIVALEAL-------KEQ------CEVILSTDSQYVRQGIT-QWIHNWKKRGWKTADKK---PVKNVDLWKRLDAALGP--H-QI-KWEWVKGHAGHPENERCDELARTAA-----MSPT------QDDIGYQTEA---------------------

>Proteobacteria gamma | Salmonella enter | ZP_03355493

--------------------------------------MLKQVEIFTDGSCLG-NP-GP----GGYGAILR---Y-R-G--------HEK-----TFSEGYTL-TTNNRMELMAAIVALEAL-------KEH------CEVTLSTDSQYVRQGIT-QWIHNWKKRGWKTAEKK---PVKNVDLWKRLDAALGQ--H-QI-KWVWVKGHAGHPENERCDELARAAA-----MNPT----Q--------------------------------

>Proteobacteria gamma | Escherichia coli | NP_285902

--------------------------------------MLKQVEIFTDGSCLG-NP-GP----GGYGAILR---Y-R-G--------REK-----TFSAGYTR-TTNNRMELMAAIVALEAL-------KEH------CEVILSTDSQYVRQGIT-QWIHNWKKRGWKTADKK---PVKNVDLWQRLDAALGQ--H-QI-KWEWVKGHAGHPENERCDELARAAA-----MNPT------LEDTGYQVEV---------------------

>Proteobacteria gamma | Pectobacterium w | YP_003258564

--------------------------------------MRKQVEIFTDGSCLG-NP-GP----GGYGALLR---Y-K-Q--------HEK-----ALSAGYRL-TTNNRMELMAAIAALETL-------TTD------CDVVLSTDSQYVRQGIT-SWIHNWKKRGWKTADKK---PVKNVDLWKRLDTAIQR--H-SV-RWEWVKGHAGHPENERCDELARAAA-----SAPT------LDDTGYQAE----------------------

>Proteobacteria gamma | Dickeya dadantii | YP_002988438

--------------------------------------MLKQVEIFTDGSCLG-NP-GP----GGYGALLR---Y-K-Q--------HEK-----TLSGGYRL-TTNNRMELMAAIAALETL-------TTE------CEVTLSTDSQYVRQGIT-QWIHNWKKRGWKTTEKK---PVKNADLWQRLDTAVQR--H-HL-HWKWIKGHSGHPENERCDVLAKQAA-----NNPT------QEDTGYQPD----------------------

>Proteobacteria gamma | Dickeya dadantii | ZP_05725899

--------------------------------------MLKQVEIFTDGSCLG-NP-GP----GGYGALLR---Y-K-Q--------HEK-----TLSAGYRL-TTNNRMELMAAIVALESL-------TSP------CEVTLSTDSQYVRQGIT-SWIHNWKKRGWKTAEKK---PVKNIDLWQRLDVAIQR--H-TL-HWMWVKGHAGHPENERCDELARQAA-----NMPT------LDDTGYQPE----------------------

>Proteobacteria gamma | Providencia rett | ZP_06126237

--------------------------------------MTKQVEIFTDGSCLG-NP-GP----GGYGVVLR---Y-Q-Q--------HEK-----TLSEGYFL-TTNNRMELLAAIKALESL-------TRP------CDIILTTDSQYVRQGIT-QWIHGWKRKQWRKADKS---PVVNVDLWKRLDDAIQR--H-TI-DWRWVKGHAGHPENEKCDELARAAA-----SAPT------KEDTGYQPAQN--------------------

>Proteobacteria gamma | Providencia rust | ZP_05973989

--------------------------------------MTKQVEIFTDGSCLG-NP-GP----GGYGIVLR---Y-Q-Q--------HEK-----TLSDGFFL-TTNNRMELLAAIIALESL-------TQP------CDVILTTDSQYVRQGIT-QWIHNWKRRQWKKADKS---PVVNVDLWKRLDQAITR--H-TI-DWRWVKGHAGHAENEKCDELARAAA-----NSPT------KEDTGYQPAQE--------------------

>Proteobacteria gamma | Photorhabdus lum | NP_928278

--------------------------------------MGKQVEIFTDGSCLG-NP-GP----GGYGVLLR---Y-Q-Q--------HEK-----TLSEGFYH-TTNNRMELMAAIIGLETL-------TRP------CKIVLTTDSQYVRQGIT-QWIHNWKKRGWRKADKS---PVSNVDLWQRLDQAISR--H-NI-DWQWVKGHAGHDENERCDELARAAA-----NSPT------ETDTGYLENRD--------------------

>Proteobacteria gamma | Proteus mirabili | YP_002150008

--------------------------------------MHKQVEIFTDGSCLG-NP-GP----GGYGAILR---Y-Q-Q--------HEK-----TLSEGFFM-TTNNRMELLAAIVALEAL-------KFP------CKITLTTDSQYVRQGIT-KWIHSWKKRQWRKADKS---PVLNVDLWKRLDKAIER--H-EI-EWHWVKGHAGHDENERCDELAKAAA-----QSPT------KEDTGYLESQQDKT-----------------

>Proteobacteria gamma | Grimontia hollis | ZP_06054290

---------------------------------------------------------------------MR---Y-K-Q--------HEK-----ELSEGFSL-TTNNRMELLAAIVGLASL-------KES------CNVTLTTDSQYVRQGIT-QWIHNWKKRDWKTADKK---PVKNADLWQRLDSETQR--H-TV-DWQWVKGHAGHPENERCDELARTAA-----ENPT------SPDTGYQPDA---------------------

>Proteobacteria gamma | Vibrio fischeri | YP_205319

----------------------------------MITEIMKQVEIFTDGSCLG-NP-GP----GGYGIVMR---Y-K-G--------TEK-----TFSEGFNK-TTNNRMEMLAAVVALRKL-------KEP------CSVILTTDSQYVRQGIT-QWIHGWKKRDWKKADKK---PVVNADLWKQLDAESER--H-KI-DWRWVKGHAGHRENEMCDELARTAA-----ENPT------QDDTGYPG-----------------------

>Proteobacteria gamma | Aliivibrio salmo | YP_002263754

----------------------------------MITEIMKQVEIFTDGSCLG-NP-GP----GGYGIVMR---Y-K-G--------TEK-----TFSGGFNQ-TTNNRMEMLAAVVALRNL-------KEP------CIVVLTTDSQYVRQGIT-QWIHGWKKRGWKKADKK---PVVNADLWKQLDAEAER--H-TV-DWRWVKGHAGHRENEMCDDLARTAA-----ENPT------QDDTGYPG-----------------------

>Proteobacteria gamma | Vibrio furnissii | ZP_05876735

--------------------------------------MKKQVEIFTDGSCLG-NP-GP----GGYGVVMR---Y-K-Q--------VEK-----TLAKGYRL-TTNNRMEMMAAVVALKTL-------KEP------CHVSLTTDSQYVRQGIT-QWIHNWKKRGWKTADKK---PVKNADLWQALDAETAR--H-QV-EWHWVKGHAGHRENEMCDELARSAA-----ENPT------EDDVGYQPEK---------------------

>Proteobacteria gamma | Vibrio cholerae | NP_231865

--------------------------------------MNKQVEIFTDGSCLG-NP-GP----GGYGIVMR---Y-K-Q--------VEK-----TLARGYRL-TTNNRMEMLAAVMALQAL-------KEP------CRVILTTDSQYVRQGIT-QWIHNWKLRGWKTADKK---PVKNADLWQALDKETAR--H-QV-EWRWVKGHAGHRENEMCDELARQAA-----ENPT------EDDIGYQPEPQ--------------------

>Proteobacteria gamma | Vibrio metschnik | ZP_05881164

--------------------------------------------------------------------------M-----------------------------------EMLAAVIALQSL-------KEP------CDVILTTDSQYVRQGIT-QWIHNWKQRGWKTADKK---PVKNADLWQALEKETAR--H-QV-DWRWVKGHAGHRENEMCDQLARSAA-----ENPT------EDDVGYQP-----------------------

>Proteobacteria gamma | Vibrio vulnificu | NP_760761

--------------------------------------MTKQVEIFTDGSCLG-NP-GP----GGYGVVLR---Y-K-Q--------VEK-----TLAQGYRL-TTNNRMEMMATIVALQAL-------KEP------CNVILTTDSQYVRQGIT-QWIHNWKKRGWKTADKK---PVKNADLWQALDKETTR--H-TI-DWRWVKGHAGHRENEMCDELARAAA-----ENPT------LDDTGYQPAE---------------------

>Proteobacteria gamma | Vibrio coralliil | ZP_05884158

--------------------------------------MTKQVEIFTDGSCLG-NP-GP----GGYGIVLR---Y-K-Q--------VEK-----TLAKGYTL-TTNNRMEMMATIVALQAL-------KEP------CDVILTTDSQYVRQGIT-QWIHNWKKRDWKTSDKK---PVKNADLWKALDAETGR--H-KI-DWRWVKGHAGHRENEMCDELARAAA-----ENPT------DEDTGYQPS----------------------

>Proteobacteria gamma | Vibrio orientali | ZP_05946276

--------------------------------------MTKQVEIFTDGSCLG-NP-GP----GGYGVVLR---Y-K-Q--------TEK-----TLAKGYTM-TTNNRMEMLATIVALQAL-------KES------CDVILTTDSQYVRQGIT-QWIHNWKKRGWKTADKK---PVKNADLWKALDQETER--H-TV-DWRWVKGHAGHRENEMCDELARGAA-----ENPT------EEDTGYIPN----------------------

>Proteobacteria gamma | Vibrio parahaemo | ZP_05120769

--------------------------------------MTKQVEIFTDGSCLG-NP-GP----GGYGIVLR---Y-K-Q--------TEK-----TLAKGYTL-TTNNRMEMLATIVALQAL-------KEP------CDVILTTDSQYVRQGIT-QWIHNWKKRGWKTADKK---PVKNADLWKALDAESER--H-NI-DWRWVKGHAGHRENEMCDELARTAA-----ENPT------EEDTGYIPN----------------------

>Proteobacteria gamma | Vibrio sp. MED22 | ZP_01065183

--------------------------------------MTKQVEIFTDGSCLG-NP-GP----GGYGIVLR---Y-K-K--------VEK-----TLAEGFTL-TTNNRMEMLAAVVALQAL-------KEP------CSVILTTDSQYVRQGIT-QWIHNWKKRDWKTADKK---PVKNADLWQRLDKETAR--H-SV-DWRWVKGHAGHRENEMCDDLARSAA-----ENPT------QEDTGYQPS----------------------

>Proteobacteria gamma | Vibrio harveyi 1 | ZP_06174988

--------------------------------------MTKHVEIFTDGSCLG-NP-GP----GGYGIVLR---Y-K-Q--------TEK-----TLAKGYTL-TTNNRMEMLAAVVALQTL-------KEP------CQVTLTTDSQYVRQGIT-QWIHNWKKRGWKTADKK---PVKNADLWQALDKETAR--H-QV-DWHWVKGHAGHRENEICDELARTAA-----ENPT------EEDTGYQAS----------------------

>Proteobacteria gamma | Glaciecola sp. H | ZP_03560606

---------------------------------------MQEVQIFTDGSCLG-NP-GP----GGYGAIMV---Y-G-K--------HRK-----EIAEGYFA-TTNNRMELLAPIKALSLL-------KKP------CRVILTTDSQYVKNGIN-QWIHNWRKNGWKTSNKQ---PVKNADLWMALDEAVKG--H-HI-DWRWVKGHSGHPENERCDELARHAA-----EAAA--KGSGQDDNGYQPA----------------------

>Proteobacteria gamma | Pseudoalteromona | YP_661935

---------------------------------------MKHIEIYTDGSCLG-NP-GP----GGYGAVLL---F-N-Q--------HSK-----ELSQGFVH-TTNNRMELLATIEALASL-------TET------CKVDLTTDSQYVKNGIN-QWIKNWRKNGWRTSDKK---PVKNVDLWKRLDEQVGR--H-DV-KWHWVKGHSGHPMNERCDVLARDAA-----SGKS-----LLPDEGFQG-----------------------

>Proteobacteria gamma | Alteromonas macl | ZP_04714008

-----------------------------------NAVAQKTIHIYTDGSCLG-NP-GP----GGYGAVLI---Y-K-Q--------HRK-----ELSDGFAH-TTNNRMELLAPIEALNSL-------NEP------CNVELTTDSQYVKNGIN-QWIHNWRKNGWRTADKK---PVKNADLWQRLDEAVKK--H-KI-NWHWVKGHSGHPENERCDDLARGAA----EANPT------KPDEGFVGK----------------------

>Proteobacteria gamma | Alteromonas macl | YP_002126679

-------------------------------------MAQKTIHIYTDGSCLG-NP-GP----GGYGAVLI---Y-K-Q--------HKK-----ELSDGFAH-TTNNRMELLAPIEALNSL-------TEP------CAVELTTDSQYVKNGIN-QWIHNWRKNGWRTSDKK---PVKNADLWQRLDEAVKK--H-QV-NWHWVKGHSGHPENERCDELARGAA----EAKPT------QIDEGFVGN----------------------

>Proteobacteria delta | Desulfovibrio ma | YP_002952549

-------------------------------MTEETKAPQQNVIIFTDGACLG-NP-GP----GGYGAVLL---R-G-D--------ERR-----EFSGGRKL-TTNNRMELLACIVALEEL-------VEP------SVVSITTDSRYVHDAIEKRWLASWQKKGWVNSEKK---PVKNQDLWLRLLPLLSR--H-KV-KFSWVRGHTGHPENERCDVLARQAA-----NSRG-----LEADAGYPG-----------------------

>Proteobacteria delta | Lawsonia intrace | YP_595131

----------------------------------MRSNYLKSVEVFTDGSCLG-NP-GA----GGWAAILR---Y-G-D--------YEQ-----EISGGFSY-TTNNRMEMIAAIYALEKL-------KES------CLVMLYTDSQYLRNAVEKQWLVFWEKNNWKTASKK---PVKNQDLWKRLQRQLER--H-NV-IFTWVRGHSGHFENERCDNLARMEA-----SRSN-----LPKDCGFINEG---------------------

>Proteobacteria delta | Desulfovibrio de | YP_002478635

---------------------------------------MQNVTIHTDGSCLG-NP-GP----GGWAAILR---LDEGD--------HRK-----EFSGGYAL-TTNNRMEMLAVIEALALL-------KSP------CTVDLYTDSRYVCDSVSKGWLWGWVKKNWIKSDKK---PVLNVDLWQRMLPLLRQ--H-KV-NFHWLKGHAGHPENERCDVLARAQA-----SRRD-----LPPDTGYKP-----------------------

>Proteobacteria delta | Desulfovibrio de | YP_389430

---------------------------------------MKQVDIFTDGSCLG-NP-GP----GGWAAVLR---Y-A-G--------TQK-----ELGGGFSG-TTNNRMEILAVIEGLEAL-------QEP------CTVNLYTDSQYVRNAVEKKWLDSWQRNGWKTAARK---PVKNKDLWLRLLPLLAR--H-TV-KFHWVRGHSGHPENELCDTIARGHA-----SRGG-----LPPDTQAAG-----------------------

>Proteobacteria delta | Desulfovibrio vu | YP_009911

-------------------------------------MSQFDVTVFTDGSCLG-NP-GP----GGWAAIMR---C-N-G--------CEK-----ELSGGFAL-TTNNRMEILAVLEALEAL-------RDP------CKVTLFTDSQYVRNAVEKKWLAGWQRNGWKTADKK---PVKNRDLWERLVPLLAK--H-SV-SFRWVRGHSGHPENERCDVLARAQA-----SRRG-----LPEDPGFTA-----------------------

>Proteobacteria delta | Desulfovibrio vu | YP_002437076

-------------------------------------MTMKNVQAFTDGSCLG-NP-GP----GGWAAVLR---C-N-G--------SER-----ELSGGFAL-TTNNRMEILAVIEALALL-------KEP------CGVDLYTDSQYVRNAVEKKWLAGWRRNGWKTSDKK---PVKNRDLWERLQPLLDL--H-QV-RFHWVRGHSGHPENERCDVLARTQA-----SSRG-----LPPDTGYRE-----------------------

>Lentisphaerae | Victivallis vade | ZP_01924103

-------------------------MQGPHSPKKETCQIVKSVQIYTDGACKG-NP-GP----GGYGAVLL---Y-K-T--------YRR-----ELSGGFRH-TTNNRMEIFAAIAAVELL-------NEP------CEITLYSDSSYLVNAVTKRWLYNWKRSGWVKRDGQ---PVNNIDLWKRFLAAVEP--H-KL-HMVWVKGHADNVENSRCDALAVAAA-----ARRN----ALPPDTGFR------------------------

>Chloroflexi | Chloroflexus agg | YP_002463237

-----------------------------------AAVSPDTVVMYTDGSALG-NP-GP----GGYGVVLR---Y-N-Q--------HYK-----ELSGGFRR-TTNNRMELMACIAGLRAL-------KRP------MRVVIYSDSKYVVDAVQEGWVQRWQAKNWMRTSTE---PAQNADLWAELVQLCTI--H-QV-QFVWVPGHSGVPDNERCHQLATAAA-----QQPNLPPDIGFEQADEQKP----------------------

>Lentisphaerae | Lentisphaera ara | ZP_01873307

--------------------------------------MKKEVLLATDGACKG-NP-GP----GGYGTILI---F-N-Q--------YRK-----EFAEGFRL-TTNNRMEMLAVIKGLEAL-------KES------CKVKVLSDSKYIVDNVKGGHPWKWQARGWVLASKK---PAKNSDLWEDLLNLLAK--H-EV-EFEWVKGHSGHELNDRADELATGAA-----EQGT-----LLEDYGFEK-----------------------

>Cyanobacteria | Synechocystis sp | NP_442483

-----------------------------------MASTPNSVTLYTDGACSM-NP-GP----GGYGAVIL---Y-G-D---G----RRE-----ELSAGYKM-TTNNRMEIMGAIAALSHL-------QEP------SQVLLYTDSRYMVDAMSKGWAKKWKANGWQRNAKE---KAKNPDLWETMLTLCEK--H-QV-TFQWVKAHAGNKENERCDRLAVAAY----QNNPN------LVDEGFGKF----------------------

>Firmicutes | Desulfotomaculum | YP_003190872

---------------------------------------MSQVEIYTDGACSG-NP-GP----GGYGVVLK---Y-G-D--------KIK-----ELSAAYRK-TTNNRMEILAAIIGLEAL-------RRP------CTVTLYSDSQYLVNAMTKGWVKRWKANNWMRNKQE---AAKNIDLWERMLPLLEQ--H-QV-DWVWVKGHADNYYNNRCDFLAVRAI-----KEQA-----LLEDEGFKK-----------------------

>Cyanobacteria | Trichodesmium er | YP_721337

-----------------------------------MTEKRTEITIYTDGACSG-NP-GP----GGYGIIIL---S-E-K--------KRQ-----ELSGGYKL-TTNNRMELMAVIVGLEQL-------EIP------SIVNLYTDSKYIVDAVTKGWAKRWRANSWKRNKKD---KAMNPDLWGKLLDLCSK--H-QV-EFSWVRGHSGNIENERCDKLAVKAS-----QKLD-----LPSDLGYQ------------------------

>Cyanobacteria | Lyngbya sp. PCC | ZP_01620565

-----------------------------------NSSKLQEVILYTDGACQG-NP-GP----GGYGIVLI---R-G-D--------HRE-----ELSGGFQF-TTNNRMEMMAAIVGLEVL-------DKK------SKVKLYSDSKYVVDAIEKGWAERWQANGWKRNKKE---LAMNPDLWEQLLKLCSQ--H-QV-KFVWVKGHAGNRENECCDRLAVQGC-----QQQN-----LLQDVGYENPEMQQISLF--------------

>Proteobacteria delta | Syntrophus acidi | YP_462765

-------------------MKATSKAKTHPPGATAAKDPQKQVIIYTDGACLG-NP-GP----GGYGVVLL---Y-G-E--------HRK-----ELSGGYRL-TTNNRMEILAAIKGLEAL-------KSA------CSVTLYSDSQYLVNAINKGWAQRWKANGWKRNARE---KALNPDLWERLLELCSR--H-DI-TFVWVRGHANNKENERCDVLSKEAA-----GRAD-----LKADPGYP------------------------

>Cyanobacteria | Arthrospira maxi | ZP_03275282

-----------------------------------MNMGITKVTIYTDGACSG-NP-GK----GGYGAVLM---C-G-S--------HRK-----EISGGFRL-TTNNRMEMMAAIAALRAL-------KFP------CSVTLYSDSKYLVDAMTLGWAKRWQKNGWRRNQKE---WAKNPDLWAQLLGLCEE--H-QV-RFVWVKGHAGDRENEICDRLAVEAT-----HRDS-----LPPDAGYENPPQPQDIDSMS------------

>Cyanobacteria | Cyanothece sp. P | YP_002374450

-----------------------------------MNDSPKKVLIYTDGACSG-NP-GS----GGYGTVLI---Y-N-N--------HRK-----ELSGGFRL-TTNNRMEMMAAIVGLETL-------TIK------CAVTLYTDSRYLVDAITKGWAKKWKANGWKRNAKE---NAKNPDLWEKLLDLCSQ--H-EV-DFVWVKGHAGHQENEYCDRLAVRAS-----QQTNLPSDEVYENKGIET-----------------------

>Cyanobacteria | Crocosphaera wat | ZP_00515066

---------------------------------------MNKVQIYTDGACSG-NP-GK----GGYGIILA---Y-N-E--------HRK-----ELSGGYRL-TTNNRMEMMAAIIALEAL-------NKP------CDVILYTDSRYVVDAITKGWAKKWQANDWQRNKKE---QAKNPDLWQRLLDLCEQ--H-QV-EFVWVKGHAGHPENEQCDRLAVAAC-----QEVELSIDAVYEEQK--------------------------

>Cyanobacteria | Cyanothece sp. C | ZP_01729710

---------------------------------------MKKVQIYTDGACSG-NP-GK----GGYGIILV---Y-N-E--------HRK-----ELSGGYRL-TTNNRMEMMAAIIGLEAL-------KTP------CEVTLYTDSRYLVDAITKGWAKKWQANGWKRNNKE---AAKNPDLWQKLLDLCKK--H-EV-KFVWVKGHAGHPENEQCDRLAVTAT-----QQLT-----LAIDEVYEL-----------------------

>Cyanobacteria | Cyanothece sp. A | YP_001805804

---------------------------------------MKKVQIYTDGACSG-NP-GK----GGYGIILV---H-N-E--------HRK-----ELSGGYRL-TTNNRMEMMAAIIGLEAL-------KMP------CDVTLYTDSRYLVDAITKGWAKKWQGNGWKRNKKE---TAKNPDLWQKLLDLCEE--H-EV-EFVWVKGHAGHPENEQCDRLAVTAA-----QQSELAIDEVYEEY---------------------------

>Firmicutes | Desulfotomaculum | YP_001113791

-------------------------------MNTNQNTNLKEITMYTDGACSG-NP-GP----GGYGVVML---Y-K-G--------HRK-----ELSAGFRD-TTNNRMELLATIVGLETL-------KEK------CNVNLYTDSQYVVNAIEKGWAKKWRANGWMRNKKE---PALNPDLWERLLKLCEF--H-NV-KFNWVKGHAGHPENERCDQLAVAAA-----KQPN-----LPLDVR--------------------------

>Firmicutes | Heliobacterium m | YP_001681348

----------------------------------MTQAKRKEVTIYTDGACLG-NP-GP----GGYGAVLI---Y-G-E--------HRK-----ELSEGFRD-TTNNRMEMLAAIKALEAL-------KEP------CQVVLYSDSRYLVDAVTQGWARRWKANGWMRNKKD---PALNVDLWERLLQLLER--H-QV-EFRWVKGHAGNPENERCDKLATAAA-----ARPD------LPLDGRC------------------------

>Proteobacteria epsilon | Heliobacillus mo | AAN87534

---------------------------------MIHNDKKRSGPLYRWVHCSG-NP-GP----GGYGVVLI---Y-G-E--------HRK-----EMSGGYQD-TTNNRMEMLAAIRGLEAL-------KEP------CRVTLYSDSRYLVDAVKQGWARRWKANNWMRNKKD---PALNVDLWKKLLDLLDK--H-DV-DFQWVKGHAGHPENERCDVLATSAA-----AKGD-----LPPDIRG-------------------------

>Bacteroidetes | Blattabacterium | YP_003284123

--------------------------------------MNQKIHIYTDGSSKG-NP-GP----GGYGIFIE-TTI-G-N---SY---NRK-----IISEGFRY-TTNNRMELLAVIVGLEKI-------EKR-----KQNIVVFTDSKYIVNTIQNNWIHQWKKNNFFQKK--------NVDLWKRFLKIYNK--N-II-DFQWIKSHNNHYINDYCDRLSVEAS-----KRKILKIDYIYEKQNKSL-----------------------

>Bacteroidetes | Chryseobacterium | ZP_03854256

----------------------------------------MRIEIYTDGACSG-NP-GK----GGYGILMR---V-P-E---KN---YQK-----TFSRGFRK-TTNNRMELLAVITALEKL-------KST-----ENEIHIYTDSKYVSDAINQNWIAGWIKRGWK--------NVKNPDLWKKFVELYNK--H-NP-KMHWIKGHAGHFENELCDKLAVAAA-----NSSDLEIDTYFENLDNNSLF---------------------

>Bacteroidetes | Flavobacteriacea | YP_003096853

--------------------------------------MSLRIEIYTDGACSG-NP-GK----GGYGIVMK---V-P-E---KN---YEK-----HFSKGFRL-TTNNRMELLAVIVALEKL-------KSP-----DNDIHIYTDSKYVSDAINKKWLLGWIKKGYK--------NVKNPDLWRRMVPLLAT--H-KT-TFHWIKGHAGHPENEICDQLAVKAA-----QSGKLETDQYFEDQKNGGLF---------------------

>Bacteroidetes | Pedobacter sp. B | ZP_01884505

-----------------------------------------MIEIYTDGAASG-NP-GP----GGYGVILR---S-G-N--------HYK-----ELSGGFRM-TTNNRMELLAVIVGLNAL-------KTP-----GQEVMIFSDSKYVVDSVEKKWVFGWVKKGFK--------DKKNKDLWLRFLEVYKL--H-QV-RFTWIKGHNAHPENERCDVLAVAAS-----KNKAA-L---AIDAPFEAEKNSQRLL---------------

>Bacteroidetes | Pedobacter hepar | YP_003093229

-----------------------------------------MIEIYTDGAASG-NP-GP----GGYGVILR---S-G-N--------HYK-----ELSAGFRL-TTNNRMELMAVIVGLNAL-------KTP-----GQEVTVFSDSKYVIDSVEKKWVFGWVKTGFK--------GKKNKDLWMQFLNSYKL--H-HV-KFVWIKGHNNHPENERCDQLAVAAS-----KNRAA-L---AIDGPFEAEKNSASLL---------------

>Bacteroidetes | Sphingobacterium | ZP_04781167

-----------------------------------------MIELYTDGASSG-NP-GP----GGYGTILRTR-YSG-ENEAFKGKLIEK-----TFSEGFRR-TTNNRMELMAVIIGLEAL-------KSP-----QQQVTIYSDSKYVIDAIDKKWVYGWIQKGFQ--------GKKNKDLWIRLMKSYKL--H-QV-RLVWVKGHAGHPDNERCDQLAVAAS-----KDKAN-W---KIDAVFEQEEKALG-----------------

>Bacteroidetes | Flavobacteria ba | ZP_03702482

-------------------------------------MKSKPVYLYTDGSSLG-NP-GP----GGYGLRLE---W-A-E---MS---YVK-----EFSQGFVR-TTNNRMELLAVIVGLELL-------KKQ-----PLEVVVFSDSKYVIDSVDKKWVFGWEKKAFK--------DKKNSDLWKRFLKIYRK--H-NV-NFQWIKGHNQHPQNERCDELAVIAA-----KGKNLIPDVFFEQIEKENSKA--------------------

>Bacteroidetes | Flavobacteria ba | ZP_03700731

-------------------------------------MHKADVHVYTDGAASG-NP-GP----GGYGIVME---W-V-G---TP---YKK-----EFSQGFTH-TTNNRMELLAVIEALRKL-------KKA-----PLKVLVFTDSKYVVDAVEKKWLQRWVKTNFK--DKK------NVDLWKAFLKEYPK--H-EV-RFQWIKGHNNHPQNERCDVLAVAAS-----KGKD-----LYIDSGFVKTT---------------------

>Bacteroidetes | Gramella forseti | YP_863387

-------------------------------------MQTPKVHIYTDGAARG-NP-GP----GGFGVVME---W-V-G---KP---YKK-----EYAQGFKL-TTNNRMELMAVIVAISKL-------KNP-----GTPAKVFTDSKYVADAVNKGWVFNWEKKNFV--NRK------NTDLWKAFLKVFRR--H-EV-QFQWIKGHNDHPQNERCDALAVMAS-----KGKD-----LLEDTGYKA-----------------------

>Bacteroidetes | Kordia algicida | ZP_02161748

---------------------------------------MVDVHIYTDGSSRG-NP-GP----GGYGIVME---W-V-G---KP---YHK-----EFSEGYRK-TTNNRMELLAVIVALEKL-------KFM-----HTEAKVFTDSKYVVDSVEKKWVFGWEKKGFS--GKK------NADLWMRFLKIYRK--H-IV-HFQWIKGHNNHPQNERCDFLAVEAS-----KKEKLKIDTFYESESNRLF----------------------

>Bacteroidetes | Flavobacteria ba | ZP_01733613

--------------------------------------MSHEVHIYTDGAAKG-NP-GP----AGYGVVME---M-V-G---TP---YKK-----EFYEGFRL-STNNRMELLAVIVGLEKL-------KNP-----KTKVLVVSDSKYVVDSVEKRWVFQWEKINFK--AKK------NPDLWMRFLKIYRQ--H-QV-DFQWVKGHNSHPQNERCDELAVMAS-----QQEKLSIDEFYEREEEKLL----------------------

>Bacteroidetes | Flavobacterium p | YP_001295134

--------------------------------------MNYQVHIYTDGAAKG-NP-GP----GGYGVVME---L-V-G---TA---FKK-----EFYEGFRH-TTNNRMELLAVIVGLEKL-------KNP-----NMKVLVVSDSKYVVDSVEKKWVLGWEKKGFK--DRK------NSDLWKRLLIIYRK--H-QV-DFKWIKGHNSHPQNERCDQLAVFAS-----NQKTLSVDAFYEKEEAKLL----------------------

>Bacteroidetes | Flavobacterium j | YP_001193487

--------------------------------------MSHEVHIYTDGAAKG-NP-GN----GGYGVVME---L-V-G---TP---YKK-----EFYEGFRL-TTNNRMELLAVIVGLEKL-------KNP-----NMKVLVISDSKYVVDSVEKKWVFGWEKKGYT--------GKKNPDLWKRFLIAYRK--H-KV-DFKWIKGHNNHPQNERCDQLAVMAS-----MQPKLSVDVYYETIGSKE-----------------------

>Bacteroidetes | Cytophaga hutchi | YP_679299

-----------------------------------------MITLYTDGSSRG-NP-GP----GGFGVVLL---Y-K-Q--------HRK-----EISGGFRM-TTNNRMELLAVITGLEAL-------KDP-----GHDVLIYSDSKYVIDSVEKGWLMGWVKKNFK--DKK------NEDLWRRYLYVSSK--H-KI-RFQWVRGHAGNIENERCDVLATQAA-----DGPNKQIDFGYETENGMLNKNHLS-----------------

>Bacteroidetes | Algoriphagus sp. | ZP_01719037

-----------------------------------------MISIYTDGAAKG-NP-GP----GGYGAVLL---F-N-N---KGSI-LRK-----ELSEGYRL-TTNNRMELLAVIRALQAL-------KVT-----GIPVQIYSDSKYVVDAIEKGWLWGWQKKGFK--DKK------NPDLWLRYIPLHLK--Y-KP-KFIWVKGHAGNPENERCDQLAVEAA-----EGRN-----LPADVGYEDSQK--------------------

>Bacteroidetes | Chitinophaga pin | YP_003123318

---------------------------------------MSEVIIYTDGSSRG-NP-GP----GGYGVVLM---W-N-S--------VRK-----ELSQGYRL-TTNNRMELMAVIVALEAL-------KRD-----GLQVKIFTDSQYVVNSVEKGWLWGWVKTGFKDK--K------NKDLWQRFIPAFKK--H-QV-KFNWVKGHSTNPLNNRCDELATQAA-----DSGN-----WLDDVGFEGE----------------------

>Thermoprotei | Metallosphaera s | YP_001192308

----------------------------------------MKALGRFDGLCEPKNP-GG---IATFGYVIY---I-NGN--------VIEGMGLASE-PWSVN-STNNVAEYTGLICLLKKM-------LTLG----VTEARVEGDSQLVIRQLKGEYSVKSK---------------RIIPLYEKAKELLAK--FSSV-EIEWIPR--EENK--EADRITRIAFKKVLNGELK-------------------------------------

>Methanomicrobia | Methanoculleus m | YP_001046256

---------------------------------------TDAVTLYTDGASRG-NP-GD----AAWAYVI----VRDGS--------VVA-----GRSGYIGT-ATNNVAEYHAVINGLDAA-------REFT----GGRLEVRSDSELVVRQLTGRYRITKE---------------HLAGLAEEVRRRMRH--FAEV-RFESVPR--EHPCIQVADRLCNETLDAERRGRR--------------------------------------

>environmental samples | uncultured archa | AAU83668

---------------------------------------MKKLIIYTDGACRG-NP-GP----AGIGIVIC---NESGK--------KIK-----EDKEFIGD-ATNNIAEYRALIKALELA-------SDFS----VTRVECFSDSELMVRQLNGAYRVKDE---------------KLGELFLQVKEKERL--FEEV-TYSHVPR--KNNLIKRADSLANLGIDDKEPKETT-------------------------------------

>Halobacteria | Haloquadratum wa | YP_657009

---------------------------------------GGRAHVYFDGACRG-NP-GP----AAIGWVLV---TNEG---------IIA-----DGGEEIGK-TTNNRAEYAALERAIEMA-------RQYG----FTEIDIRGDSQLIIRQVTGEYDTNEP---------------TLREYRVRVRELLQT--FDRW-SIEHVPR--DVNS--HADKLANEAFDHG-------------------------------------------
